# Supplementary material for: Inhibition of topoisomerase 2 catalytic activity impacts the integrity of heterochromatin and repetitive DNA and leads to interlinks between clustered repeats
Source: Nat Commun. 2024 Jul 8;15:5727. doi: 10.1038/s41467-024-49816-7 (PMC11231352; doi:10.1038/s41467-024-49816-7)

Figure S1: ICRF-193 induces damage at heterochromatin

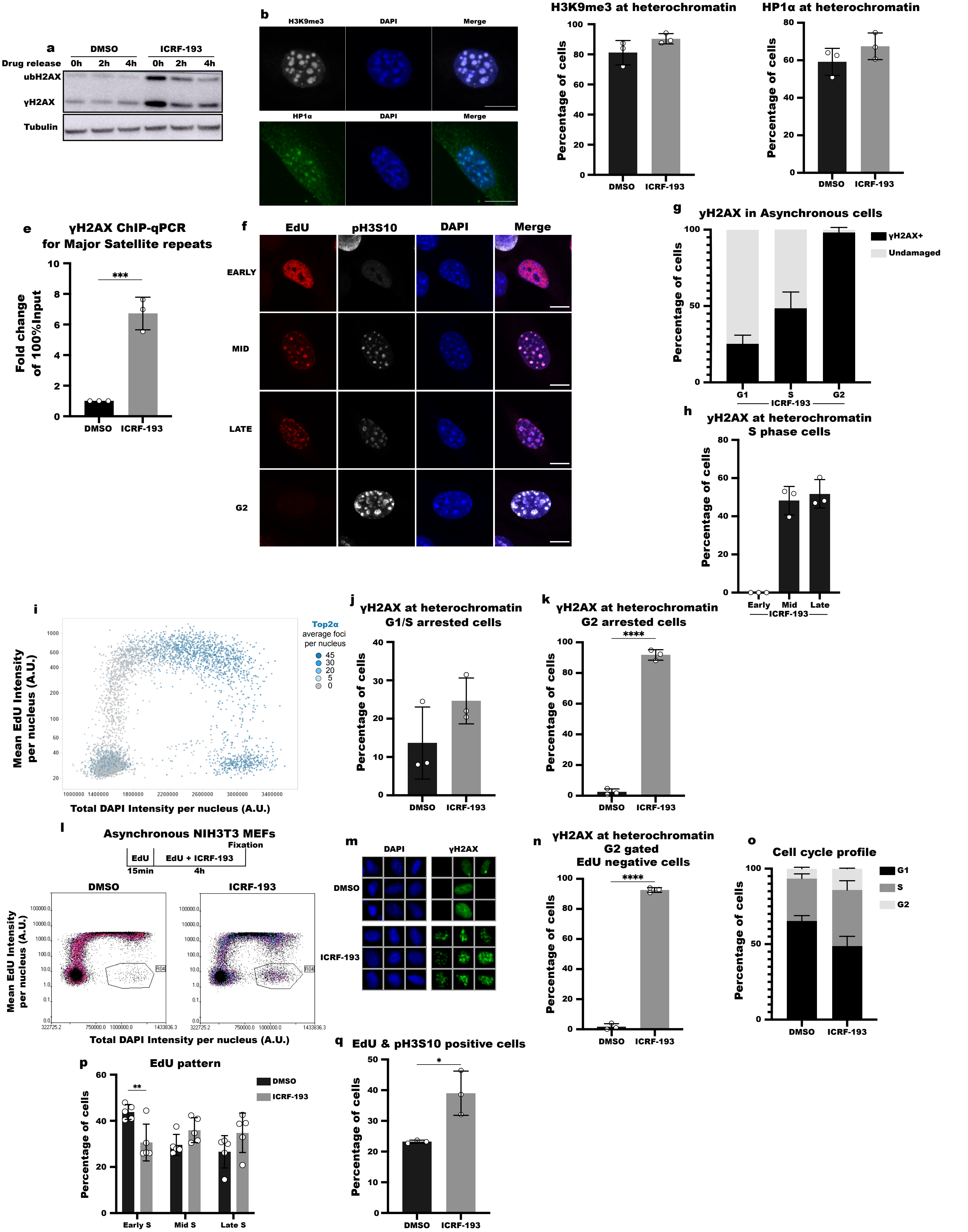

Figure S2: ICRF-193 induces damage mainly by targeting Top2α

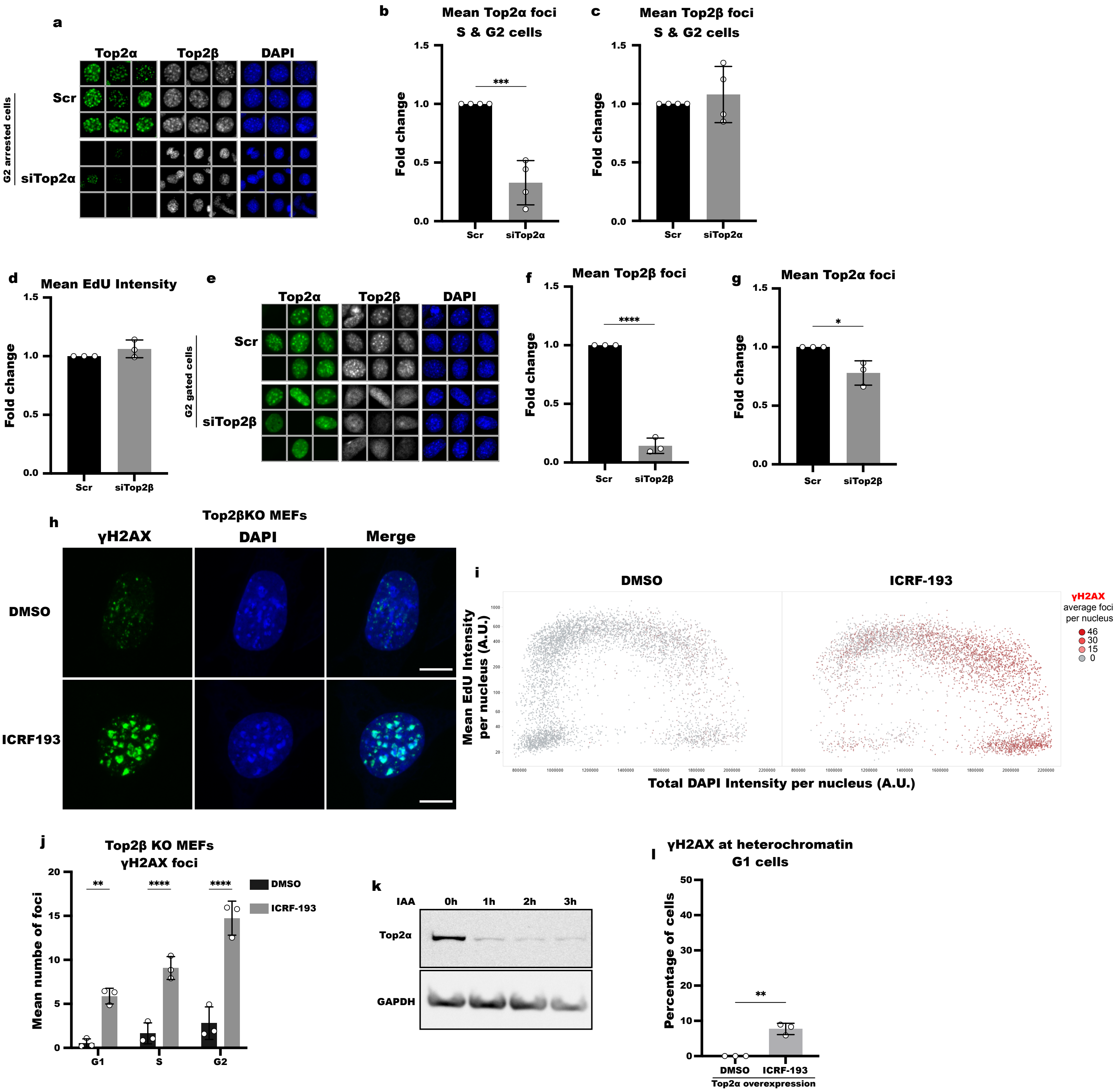

Figure S3: ICRF-193 induces DSBs in repetitive sequences

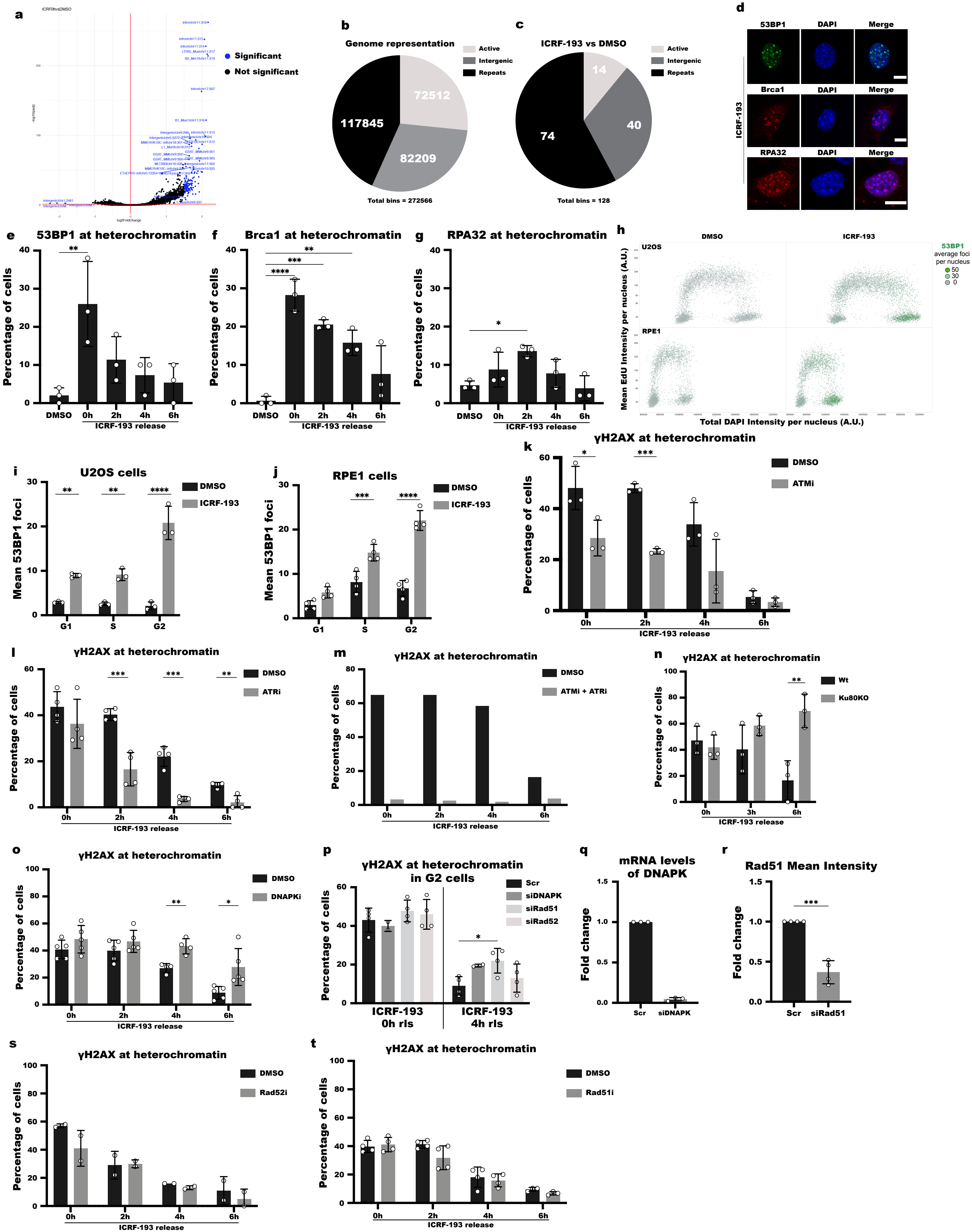

Figure S4: ICRF-193 induces genomic instability at heterochromatin

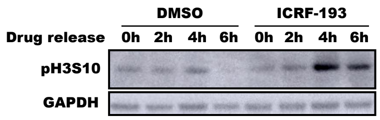

Figure S5: ICRF-193 affects the localization of Topoisomerase 2 α & β

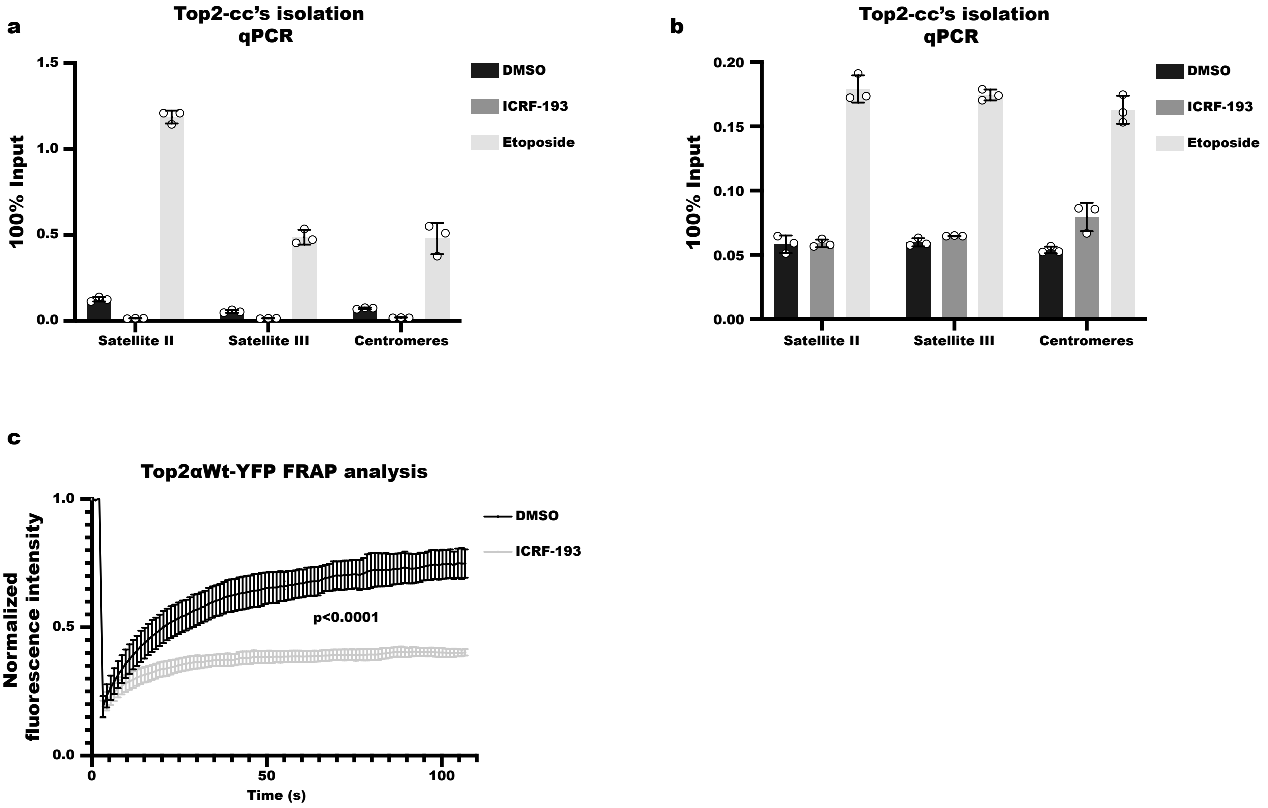

Figure S6: Sumoylation is necessary for DDR activation

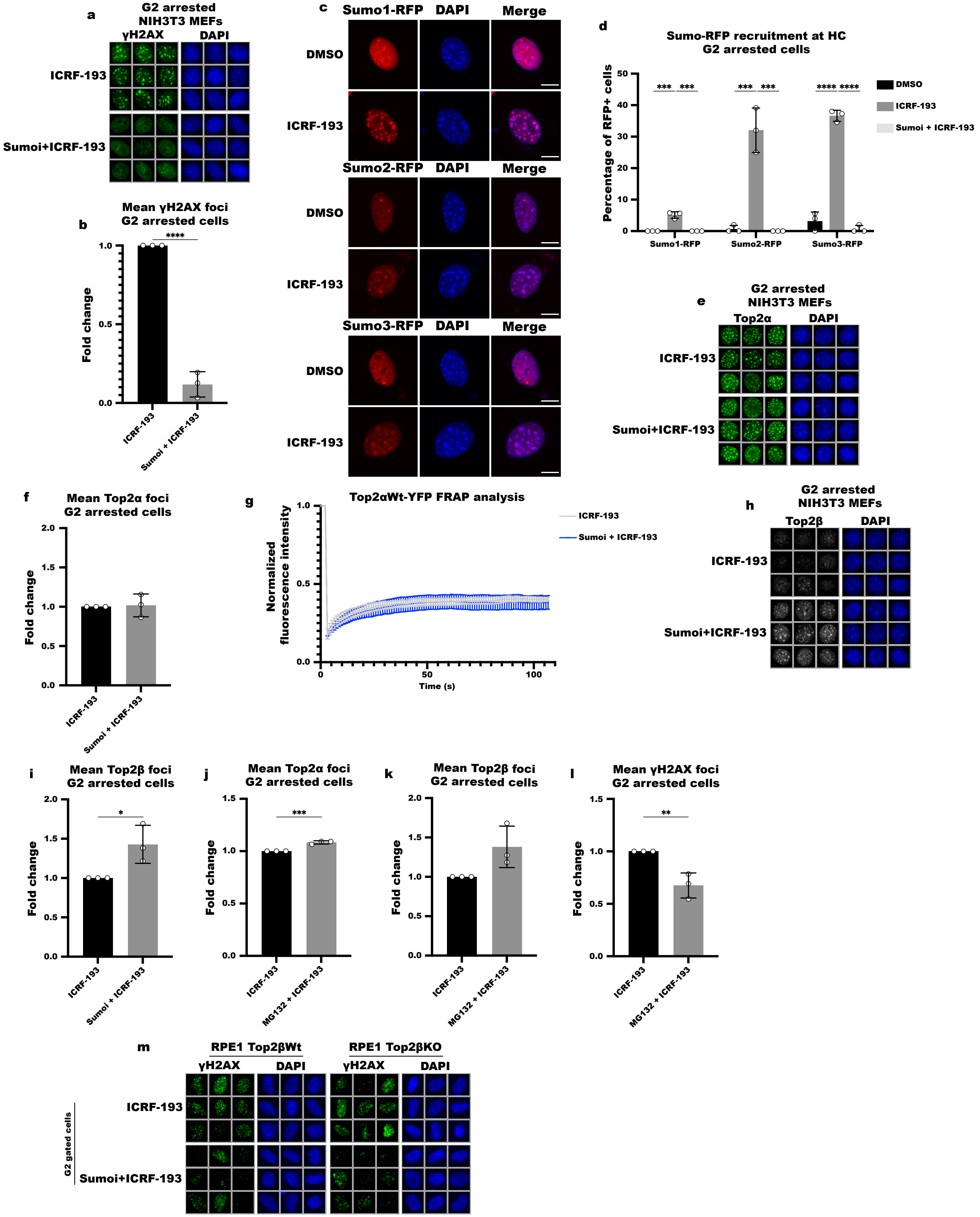

Figure S7: Ercc1-XPF endonuclease safeguards genomic stability by resolving inter-chromosomal DNA bridges

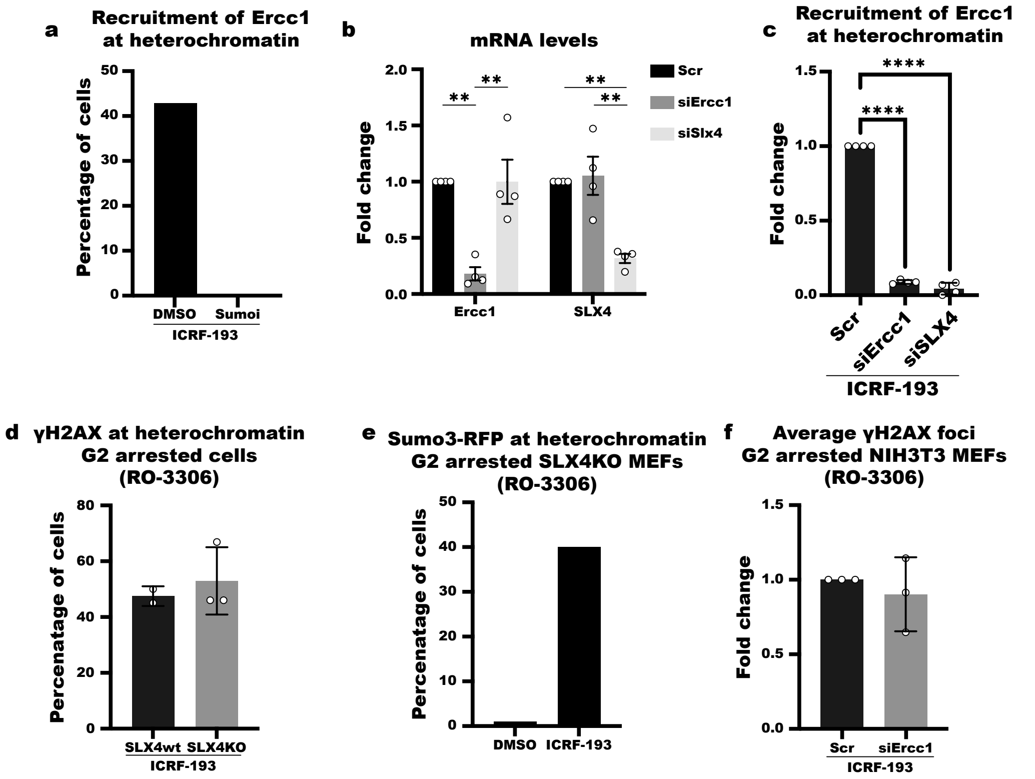

Figure S1: ICRF-193 induces damage at heterochromatin on a cell cycle basis

a) Western blot analysis of γH2AX, ubiquitinated H2AX (ubH2AX) and Tubulin after treatment with ICRF-193 and release in drug free medium for the indicated time.
b) Representative confocal microscopy images of H3K9me3 (white), HP1α (green) and DAPI (blue).
c) Quantification of cells with H3K9me3 at heterochromatin.
d) Quantification of cells with HP1α at heterochromatin.
e) ChIP-qPCR for γH2AX levels at heterochromatin over input.
f) Representative confocal microscopy images of EdU (red), DAPI (blue) and pH3S10 (white) for early, mid and late S (EdU positive) or G2 phase cells (EdU negative and pH3S10 positive cells).
g) Quantification of cells with γH2AX at heterochromatin in G1, S or G2.
h) Quantification of cells with γH2AX at heterochromatin in S phase cells.
i) QIBC plot of Top2α in untreated (DMSO) cells. At least 1000 cells were analyzed per condition. A.U.: Arbitrary Units
j) Quantification of cells synchronized in G1/S border, by double thymidine block, with γH2AX at heterochromatin.
k) Quantification of cells synchronized in G2, by RO-3306, with γH2AX at heterochromatin.
l) Visual representation of the order of drug treatment applied in cells, to select the EdU negative and G2 positive cell population using QIBC.
m) Representative wide field images of γH2AX (green), and DAPI (blue). The cells depicted are shown from the EdU negative G2 population as gated in figure S1l.
n) Quantification of cells with γH2AX at heterochromatin, from figure S1l.
o) Analysis of the cell cycle profiles of cells, using QIBC. At least 1500 cells were analyzed per condition.
p) Quantification of S phase cells based on their EdU patterns; n=5 biologically independent experiments.
q) Quantification of cells simultaneously positive for EdU and pH3S10.
Graphs represent data and average values from experiments performed in NIH3T3 cells, and error bars represent the standard deviation from at least 50 cells per condition, and 3 independent replicates unless stated otherwise. For confocal images maximum projection of z-stacks is shown; scale bars = 10μm. Statistical significance presented by student t-test in S1c, S1d, S1e, S1j, S1k, S1n, and S1q and two-way ANOVA in S1p: (*) $p<0.05$, (**) $p<0.01$, (***) $p<0.001$, (****) $p<0.0001$

Figure S2: ICRF-193 induces damage by mainly targeting Top2α

a) Representative wide field images of Top2α (green), Top2β (white) and DAPI (blue) in G2-sunchronized NIH3T3 cells, with RO-3306, after transfection with non-targeting (Scr) or Top2α siRNA.
b) Fold change quantification of Top2α foci in comparison to the control (Scr). At least 320 cells were analyzed per condition. n=4 biologically independent experiments.

c) Fold change quantification of Top2β foci in siTop2α over Scr. n=4 biologically independent experiments.
d) Fold change quantification of EdU levels in comparison to Scr.
e) Representative wide field images of Top2α (green), Top2β (white) and DAPI (blue) in G2-seleted NIH3T3 cells, after transfection with Scr or siTop2β.
f) Fold change quantification of Top2β foci in siTop2β over Scr.
g) Fold change quantification of Top2α foci in siTop2β over Scr.
h) Representative confocal microscopy images of γH2AX (green) and DAPI (blue) in Top2βKO MEFs. Maximum projection of z-stacks shown. Scale bars = 10μm.
i) QIBC plots of γH2AX foci in Top2βKO MEFs. A.U.: Arbitrary Units.
j) Quantification of the γH2AX foci presented in fig. S2h.
k) Western blot analysis of Top2α levels in HCT116-OsTIR1/Top2α-AID cells after treatment with IAA. GAPDH was used as a protein-loading control.
l) Quantification of G1 NIH3T3 cells with γH2AX at heterochromatin overexpressing human Top2α-YFP and treated with DMSO or ICRF-193. Only G1 cells were selected for quantification, by choosing nuclei negative for EdU and pH3S10. The positive cells where counted based on the colocalization of the γH2AX foci with the Top2α-YFP foci forming the heterochromatin domains. At least 23-56 cells were counted per replicate. Graph bars represent average values, and the error bars in the graphs represent the standard deviation from at least 1000 cells per condition, and 3 independent replicates unless stated otherwise. Statistical significance presented by student t-test in S2b, S2c, S2d, S2f, S2g and S2l, and two-way ANOVA in S2j: (*) $p<0.05$, (**) $p<0.01$, (***) $p<0.001$, (****) $p<0.0001$

Figure S3: ICRF-193 induces DSBs in repetitive sequences

a) Scatter plot showing the distribution of non-significant (black) and significant (blue) γH2AX enriched bins in ICRF-193 over DMSO treatment.
b-c) Pie charts representing the overall distribution of total number of bins in the three main categories (b) or bins with a significant γH2AX enrichment after treatment with ICRF-193 (c) as shown in fig. S3A.
d) Representative confocal microscopy images of 53BP1 (green), Brca1 (red), RPA32 (red) and DAPI (blue). Maximum projection of z-stacks is shown. Scale bars = 10μm.
e-g) Quantification of cells with 53BP1 (e), Brca1 (f) and RPA32 (g) at heterochromatin.
h) QIBC plots of 53BP1 foci in U2OS (Upper panel) or RPE1 (Lower panel) cells treated with DMSO or ICRF-193. At least 1000 cells were counted per condition. A.U.: Arbitrary Units
i-j) Quantification of the mean number of γH2AX foci in U2OS cells (i) or RPE1 (j) cells. n=4 biologically independent experiments for j.
k-m) Quantification of γH2AX induction in cells treated with ICRF-193 and pre-treated with ATMi (k), ATRi (l) or ATMi+ATRi (m). n=4 (l) and n=1 (m) biologically independent experiments.
n) Quantification of γH2AX induction in Wt or Ku80KO MEFs treated with ICRF-193 and released.

o) Quantification of γH2AX induction in cells pre-treated with DNAPKi, next ICRF-193 and released. n=4 for the 4h and n=5 for the rest of the time points.
p) Quantification of γH2AX induction in G2-selected cells, transfected with the indicated siRNAs, treated with ICRF-193 and subsequently released. n=2 for siDNAPK and n=4 biologically independent experiments for the other conditions.
q) Quantification of DNAPK mRNA levels by qRT-PCR after transfection with siDNAPK. n=1 biological experiment, and the error bars represent the standard deviation from 3 technical replicates.
r) Fold change of Rad51 mean intensity, after transfection of siRad51. n=4 biologically independent experiments.
s-t) Quantification of γH2AX induction in cells pre-treated with Rad52i (s) or Rad51i (t), next ICRF-193 and released. n=2 (s) and n=4 (t) biologically independent experiments.
Graphs represent average values; error bars in graphs represent the standard deviation from at least 50 cells per condition, and 3 independent replicates unless stated otherwise. Experiments were done in NIH3T3 cells, unless stated otherwise. Statistical significance presented by one-way ANOVA for S3e, S3f, S3g and S3p, two-way ANOVA for S3i and S3j and student *t*-test for S3k, S3l, S3n, S3o, S3r, and S3t: (*) $p<0.05$, (**) $p<0.01$, (***) $p<0.001$, (****) $p<0.0001$

Figure S4: ICRF-193 induces genomic instability at heterochromatin

Western blot analysis of pH3S10, and GAPDH of cells treated with DMSO or ICRF-193 and released in drug free medium for the indicated time.

Figure S5: ICRF-193 affects the localization of Topoisomerase 2 α & β

a-b) Quantification of Top2-cc's at Satellite II, Satellite III and centromeric repeats in U2OS cells treated with DMSO, ICRF-193 or etoposide, with (a) representing the first and (b) the second biological replicate. The cells were synchronized in G2 phase, with RO-3306. Graphs represent average values, and the error bars represent technical replicates from 1 biological experiment.
c) FRAP analysis of Top2α Wt mobility in NIH3T3 cells transfected with Top2αWt and treated with DMSO or ICRF-193. The data from the ICRF-193 curve are also presented in Fig. S6g for comparison with Sumoi, since the experiments for these conditions were done simultaneously.
Graph curves for FRAP results represent average values, and the error bars represent the standard error of the mean from at least 20 cells per condition, and 3 biological replicates. Statistical significance is presented by one-way ANOVA test in S5c.

Figure S6: Sumoylation is necessary for ICRF-193 dependent DDR activation
a) Representative wide field images of γH2AX (green) and DAPI (blue) in G2-arrested NIH3T3 cells, treated with ICRF-193 and pre-treated with ML-792 sumo inhibitor (Sumoi).
b) Quantification of the γH2AX foci, in G2 arrested NIH3T3 cells treated with ICRF-193 and pre-treated with Sumoi.
c) Representative confocal microscopy images of RFP-Sumo1, RFP-Sumo2 and RFP-Sumo3 (red) and DAPI (blue) in G2-arrested NIH3T3 cells, transiently transfected with Sumo-1, 2 or 3-RFP and treated with ICRF-193. Maximum projection of z-stacks shown. Scale bars = 10μm.
d) Quantification of cells with Sumo1, 2 or 3 at heterochromatin, shown in Fig. S6c. At least 50 cells were counted per condition.
e) Representative wide field images of Top2α (green) and DAPI (blue) in G2-arrested NIH3T3 cells, pre-treated with Sumoi prior to the addition of ICRF-193.
f) Quantification of the mean number of Top2α foci, in G2-arrested NIH3T3 cells treated with ICRF-193 and pre-treated with Sumoi.
g) FRAP analysis in NIH3T3 cells transiently transfected with Top2αWt-YFP and treated with ICRF-193, after pre-treatment with Sumoi. The data from the ICRF-193 curve are also presented in Figure S5c. At least 20 cells were analyzed per condition.
h) Representative wide field images of Top2β (white) and DAPI (blue) in G2-arrested NIH3T3 MEFs, pre-treated with Sumoi prior to addition of ICRF-193.
i) Quantification of the Top2β foci, in G2 arrested NIH3T3 cells pre-treated with Sumoi, prior to addition of ICRF-193.
j-l) Quantification of the Top2α (j), Top2β (k) or γH2AX (l) foci in G2-arrested NIH3T3 cells pre-treated with MG132, prior to addition of ICRF-193.
m) Representative wide field images of γH2AX (green) and DAPI (blue) in G2-gated RPE1 Top2βWt and Top2βKO cells, pre-treated with DMSO or Sumoi prior to addition of ICRF-193.
Graphs represent average values, and the error bars in the graphs represent the standard deviation from at least 450 cells per condition, and 3 independent replicates unless stated otherwise. Statistical significance presented by student *t*-test in S6b, S6f, S6i, S6j, S6k, S6l and one-way ANOVA in S6d and S6g: (*) $p<0.05$, (**) $p<0.01$, (***) $p<0.001$, (****) $p<0.0001$

Figure S7: Ercc1-XPF endonuclease safeguards genomic stability by resolving inter-chromosomal DNA bridges

a) Quantification of NIH3T3 cells with Ercc1 at heterochromatin as shown in Fig. 7a, in the presence of ICRF-193 with or without pre-treatment with Sumoi. Data presented from a single biological replicate.
b) Quantification of Erccc1 or SLX4 mRNA levels by qRT-PCR analysis after transfection of the indicated siRNAs in NIH3T3 cells. Graph bars represent mean

values, and the error bars represent the standard error of the mean (SEM) from n=4 biologically independent experiments.
c) Quantification of asynchronous NIH3T3 cells with Ercc1 at heterochromatin, after scramble siRNA (Scr), siErcc1 or siSLX4. Graph bars represent mean values, and the error bars represent the standard deviation from n=4 biologically independent experiments.
d) Quantification of G2-arrested SLX4 wt or KO MEFs with γH2AX at heterochromatin, after treatment with ICRF-193. Graph bars represent mean values, and the error bars represent the standard deviation from n=2 biologically independent experiments for wt and n=3 biologically independent experiments for the SLX4KO condition.
e) Quantification of G2-arrested SLX4KO MEFs transiently transfected with SUMO3-RFP at heterochromatin, with or without treatment with ICRF-193. Graph bars represent values from 1 biological replicate.
f) Quantification of γH2AX foci, by the ScanR software, in G2-arrested NIH3T3 cells treated with ICRF-193, after transient depletion of Ercc1. Graph bars represent the fold change of mean values and the error bars represent standard deviation from n=3 biologically independent experiments.
Statistical significance presented by student t-test in S7f and one-way ANOVA in S7b & S7c: (*) $p<0.05$, (**) $p<0.01$, (***) $p<0.001$, (****) $p<0.0001$

Table S1: Repetitive sequences from ChIP-seq analysis

Normalized values of γH2AX ChIP-seq reads by DESeq2 analysis

2 Biological replicates	Average Input values			Average γH2AX ChIP values		
**Repeat family name**	**DMSO**	**ICRF-193 0h release**	**ICRF-193 3h release**	**DMSO**	**ICRF-193 0h release**	**ICRF-193 3h release**
(A)n	20942.8937	21043.4664	20599.4211	19680.8514	21017.2281	20422.6509
(AAATG)n	172.01108	177.88007	180.057365	146.782527	146.124639	166.206668
(AACTG)n	171.34841	174.859044	168.705795	151.150894	131.054688	162.269801
(AAGTG)n	25.5914321	25.30651	28.2404287	27.5130127	33.127386	38.5002162
(AATAG)n	225.51591	251.676903	248.079715	207.049737	218.92443	224.605535
(AATTG)n	68.8778291	63.8380584	56.5136994	77.8217388	60.1545982	72.1221618
(ACATG)n	112.806974	112.98127	113.093082	116.375608	114.734082	134.770517
(ACCG)n	33.4878993	34.1231348	42.4263268	46.8326161	48.8427185	54.3170921
(ACCTG)n	66.4903301	61.2254928	64.0571419	90.0691838	76.8799418	94.3611471
(ACGTG)n	9.12953656	7.67366524	10.6804759	11.591762	10.8269278	15.161747
(ACTAG)n	75.9218427	96.4910811	75.5472074	77.9535359	91.5451558	83.0572272
(ACTCG)n	7.89646722	4.08176697	11.4172535	9.89258565	8.64525512	17.2685011
(ACTG)n	595.332393	598.375686	611.915503	653.306044	690.666989	631.823875
(ACTTG)n	9.95577698	14.8573606	6.1426993	6.19934066	6.58512559	8.12402874
(AGATG)n	65.6840116	59.5924622	81.4129152	59.5261896	66.4164225	58.790528
(AGCTG)n	235.760033	226.698355	239.135529	290.53177	341.170991	309.041933
(AGGGGG)n	1097.36883	1144.50603	1155.02974	1428.93579	1433.10036	1485.9682
(AGGTG)n	316.476271	322.779964	317.469966	378.556147	434.89782	421.711785
(AGTAG)n	97.4271598	93.3890516	74.3363871	97.9018023	102.25054	102.83073
(AGTTG)n	53.7276426	47.1843439	52.1072902	43.007574	30.0973528	43.6024343
(ATAAG)n	68.0778019	58.8576592	82.4781115	56.1278368	53.8927738	53.7673502
(ATAGG)n	92.8692068	105.14418	97.2365779	113.272147	109.784906	123.246189
(ATATG)n	380.783041	450.292555	370.379718	349.847917	360.079229	386.750188
(ATCTG)n	34.642329	34.7758209	40.8870879	39.6945591	28.7239331	27.32803
(ATG)n	5794.49653	5854.77243	5799.62162	5435.44716	5471.41698	5370.13664
(ATGGTG)n	3294.33776	3238.82118	3229.70544	3393.78933	3391.94547	3379.05997
(ATGTG)n	367.33147	406.291245	340.778516	352.544616	359.794814	388.32696
(ATTAG)n	69.6254299	72.164612	84.9183374	94.5574893	75.1631672	97.4093046
(ATTG)n	2310.0265	2307.70524	2320.78923	2151.91234	2335.48315	2182.47168
(ATTTG)n	260.144609	243.922132	220.69101	229.523539	249.627056	228.466901
(C)n	686.693292	712.827877	711.1161	843.473073	940.150007	945.240444
(CA)n	287137.849	286907.892	284875.833	282208.39	287569.994	287556.265
(CAA)n	15247.2734	15597.1931	15202.2176	14142.8947	13777.5718	13801.7594
(CAAA)n	36057.2389	36617.4073	36507.5339	33718.3403	34985.8537	33770.9801
(CAAAA)n	28507.8514	28790.5954	28753.2599	25920.461	26971.606	25940.6171
(CAAAAA)n	11869.2615	11965.6326	11821.9754	10720.7077	10898.4172	10542.2722
(CAAAC)n	4386.96042	4404.87951	4360.61055	4022.07941	4106.71052	4215.92006

(CAAAG)n	729.848623	708.908017	727.120393	678.476056	678.506749	627.959461
(CAAAT)n	335.240211	308.657912	308.704247	323.526332	323.215656	328.693829
(CAACC)n	221.075393	278.861985	228.36582	235.765548	265.806677	244.227544
(CAACG)n	13.7336247	11.7554322	14.1858981	16.8135077	6.58512559	13.1735533
(CAACT)n	59.1841843	66.1229656	62.8862917	51.8836861	67.668299	45.590628
(CAAG)n	1711.18363	1711.86101	1667.93241	1704.93115	1878.72728	1836.99981
(CAAGA)n	316.461593	315.18963	275.322795	362.098664	338.689734	337.828668
(CAAGC)n	130.751488	127.02252	118.400479	105.970995	123.116807	126.712355
(CAAGG)n	258.596981	241.881553	236.523966	252.478069	261.889397	308.011856
(CAAGT)n	2.67584444	9.14306897	9.81233083	9.08566637	2.86838257	9.14447234
(CAAT)n	2259.09214	2216.60059	2265.24003	2227.82535	2360.08622	2251.52239
(CAATA)n	261.594199	293.880949	264.435976	257.909369	256.09186	237.282039
(CAATC)n	44.5718928	45.2248691	53.942106	53.0708354	50.6609826	57.3323161
(CAATG)n	34.7209687	29.5514991	33.7120342	37.956504	38.3390919	33.5033852
(CAATT)n	64.3261674	65.1434305	70.6010719	66.6177874	64.3964004	70.9172909
(CACAA)n	443.613483	388.332259	436.222413	440.925994	443.159613	461.61752
(CACAC)n	910.706662	913.074131	882.144374	1073.71012	1051.79389	1038.83449
(CACAG)n	1377.93143	1388.67127	1409.61342	1776.32835	2181.94081	2182.50461
(CACAT)n	304.723317	270.044753	282.160138	254.491577	276.169317	300.035781
(CACCAT)n	3405.49382	3397.19054	3469.31717	3521.78751	3770.94933	3693.54663
(CACCC)n	1790.31135	1770.31446	1736.48303	2370.27937	2547.79874	2482.39903
(CACCG)n	29.6051988	25.7961764	22.2290969	24.3317948	21.1287965	40.0111216
(CACCT)n	275.996785	263.513946	283.92927	294.818589	334.667912	330.882671
(CACG)n	2098.65576	2105.66476	2011.20754	2402.78562	2433.97379	2481.99724
(CACGC)n	34.0520076	31.674196	23.8011777	41.3083977	46.984347	45.6433215
(CACGT)n	7.79161434	17.959289	17.757004	12.2280056	11.1502291	15.2012671
(CACTA)n	193.942624	160.981724	187.962068	173.527499	191.775674	173.481507
(CACTC)n	201.294905	210.941451	233.232016	279.366697	267.88747	285.815438
(CACTG)n	438.137019	440.00683	433.028988	619.377044	620.04704	689.304145
(CACTT)n	11.7267413	12.897987	7.97751507	17.1937378	10.8269278	8.13720211
(CAG)n	5523.15521	5497.38554	5469.34232	6762.92214	6847.10652	7031.50867
(CAGA)n	14975.3282	14796.778	14942.8771	15238.3694	15353.6258	15286.311
(CAGAA)n	365.868773	324.90256	340.328023	345.976904	353.129473	337.337714
(CAGAC)n	217.783014	245.309622	225.49865	288.17312	598.755983	455.201997
(CAGAG)n	746.93282	734.215033	787.008147	988.882125	976.389472	1033.22864
(CAGAGA)n	12282.0084	12059.754	12253.8122	12581.181	12444.6296	12842.7881
(CAGAT)n	27.6245287	26.6124893	25.5374679	25.4724849	39.0458554	27.4465904
(CAGC)n	585.376616	596.172794	619.186918	778.24013	815.558901	782.799389
(CAGCC)n	721.308884	680.337267	709.290576	900.826937	933.162586	945.566239
(CAGCG)n	30.7596285	32.6535287	32.613996	37.8711662	52.5594615	38.5792565
(CAGCT)n	306.389428	312.657158	290.611696	423.12352	383.833321	365.120225
(CAGG)n	2605.95476	2649.34363	2655.10592	3389.63149	3555.535	3636.70733

(CAGGA)n	389.767881	353.311099	412.280575	450.686782	481.499315	519.97028
(CAGGC)n	263.391377	240.002879	256.024388	318.16093	418.273966	398.42601
(CAGGG)n	476.963501	464.415113	482.808835	660.060569	752.295886	688.66573
(CAGGT)n	49.8187288	38.6947704	43.2616301	27.1948909	58.0944687	34.9945305
(CAGT)n	507.97897	554.537395	549.331916	557.495507	595.93076	565.870482
(CAGTA)n	373.010825	364.08639	394.385075	425.683656	703.492664	601.733962
(CAGTC)n	49.4118999	53.388403	71.4035332	55.064904	47.186105	51.7001162
(CAGTG)n	544.739327	583.845477	599.843576	778.077035	827.538047	798.761173
(CAGTT)n	141.716998	167.34941	180.352941	177.794879	126.934429	170.890878
(CAT)n	5751.36793	5590.61217	5599.98606	5306.76151	5179.99662	5240.34319
(CATA)n	19840.8102	19750.2247	19601.4841	18447.1043	19513.7503	18861.5069
(CATAA)n	172.08972	169.144955	171.671491	165.202292	183.655478	185.058528
(CATAC)n	186.486014	174.206965	187.021111	174.543972	169.150694	184.521959
(CATAG)n	121.884084	151.512414	136.223166	125.825855	152.830087	152.045605
(CATAT)n	422.173699	388.494976	413.043066	355.589758	337.920978	328.548922
(CATATA)n	10088.5778	10091.0691	10240.5635	9304.465	9634.55507	9483.55275
(CATCC)n	310.927459	366.291001	299.851458	401.658612	397.791772	417.422777
(CATCG)n	13.0646636	7.34701866	10.5491084	9.89258565	15.3920313	2.04088721
(CATCT)n	90.7113355	85.5522654	69.4466426	55.0454647	70.6582248	67.9481737
(CATG)n	3048.41853	3137.75816	3050.39478	3399.11206	3404.60644	3491.56161
(CATGC)n	113.895347	148.574011	138.02514	165.838536	154.890217	179.824576
(CATGT)n	126.790148	142.042293	122.905413	151.445786	121.884374	121.715524
(CATTA)n	63.3358324	63.6742292	66.1946627	52.2250375	70.3349236	61.8255121
(CATTC)n	74.4591457	73.4698831	74.3363871	93.4945565	94.6152964	72.5006498
(CATTG)n	48.2448876	35.5923361	41.5581818	50.1845097	28.8855837	37.5192927
(CATTT)n	229.319447	171.75833	217.07777	171.851552	169.111807	171.315473
(CCA)n	8229.53172	8360.77781	8125.11004	9358.48701	9655.71176	9611.13916
(CCAA)n	4866.61969	5039.50194	4911.50742	4867.80306	5146.0397	5082.47133
(CCCA)n	1830.17011	1859.86203	1755.36162	2259.98646	2420.74521	2481.18403
(CCCCAA)n	211.696831	230.207731	249.823133	294.44215	297.318167	299.002164
(CCCCAG)n	1458.28593	1505.32727	1490.32953	2016.72304	2149.82404	2085.30608
(CCCCCA)n	2775.85935	2910.24734	2834.89342	3661.91417	3925.21239	3991.18584
(CCCCCG)n	263.535549	250.452737	243.337759	298.344949	320.083523	312.830847
(CCCCCT)n	1143.01022	1156.34439	1137.19993	1391.64634	1471.44189	1474.38813
(CCCCG)n	1411.53624	1413.08472	1339.6949	1287.58786	1196.16409	1207.79341
(CCCG)n	225.771227	238.208347	224.811135	217.217776	220.074817	187.132349
(CCCGA)n	7.94889365	7.02037208	4.43925101	3.14233925	8.28184649	4.05542768
(CCCGAA)n	21.0921969	35.9189827	22.9658745	47.1275083	43.2274966	54.6692334
(CCCGG)n	78.466621	86.0419317	62.7220824	52.0543618	68.7597459	53.7541768
(CCCTA)n	30.8644813	30.0413678	23.8997033	38.5927476	46.4592878	49.7119225
(CCCTAA)n	1848.29078	1963.37358	1858.32757	2101.09917	2202.4911	2198.35796
(CCCTAG)n	182.051788	198.206889	214.130659	260.473783	270.230794	251.317956

(CCCTG)n	637.936723	678.867357	689.060505	976.355925	1959.56094	1547.45169
(CCG)n	2293.50222	2375.7174	2341.93823	2292.65153	2233.45381	2193.72694
(CCGAA)n	16.3570427	13.0614115	12.7358921	25.984512	8.96855633	15.2144405
(CCGAG)n	44.6767457	42.6129106	41.492498	29.7630948	28.3605245	22.3180256
(CCGCG)n	161.36642	193.147003	169.494	152.508719	121.359315	137.022178
(CCGG)n	12.4743421	12.897987	7.9446732	17.7057649	17.0887522	10.1517426
(CCGGA)n	12.4743421	12.7349672	11.4500954	6.7502464	13.5336598	13.1867266
(CCGGG)n	80.6832101	87.8381844	73.3040327	58.6571621	64.558051	48.6783055
(CCGTA)n	5.19440956	2.93880746	1.8019739	3.69324499	8.12019588	11.1458394
(CCTA)n	788.894926	685.317261	705.92365	800.058249	827.559932	833.436495
(CCTAA)n	53.1373211	43.9190922	47.7994067	49.1680361	45.6109273	55.7160238
(CCTAG)n	73.8363197	84.2466908	68.1280041	79.1406852	77.0816998	119.714157
(CCTAT)n	81.4376262	68.899158	97.0909539	96.5050987	95.1804631	106.761011
(CCTCG)n	71.5012471	89.144366	72.26455	98.669843	90.0903004	84.0644974
(CCTG)n	2828.98408	2886.3272	2884.73182	3811.02833	3839.19918	3972.60708
(CCTTG)n	143.881161	155.431161	181.826497	193.785818	180.988854	184.614173
(CG)n	661.081414	662.377472	665.472799	637.280504	746.721382	753.7137
(CGA)n	227.870381	257.799958	234.564911	222.288285	261.948947	243.855643
(CGAA)n	77.5549256	85.2258212	70.8966487	73.3448042	82.7583038	75.4961138
(CGAAA)n	33.9733679	29.7147212	32.7453635	51.4181182	39.8340548	36.5383692
(CGAAG)n	5.86337067	9.63273531	7.9446732	5.94332712	11.6752883	11.1194927
(CGAAT)n	1.33792222	1.30618162	0.90098695	2.16474429	0	1.02044361
(CGAG)n	244.777901	236.902469	263.534989	274.57164	265.584254	296.112088
(CGAGA)n	19.3542611	21.796324	17.7241622	17.4265217	15.3920313	25.3266629
(CGAGG)n	70.8847124	80.0008923	62.8041871	76.7393667	106.088827	80.1276301
(CGAT)n	37.3443867	38.5309413	24.6364809	30.5700141	34.2990476	50.6269791
(CGATA)n	9.82471089	14.3675931	9.64812149	9.34167991	5.93852315	10.1253958
(CGATG)n	16.4094691	14.693835	14.9883595	25.4336062	25.8555505	14.2203436
(CGCGG)n	157.674027	190.207285	153.039213	133.445129	113.320555	106.991545
(CGCTG)n	30.8644813	34.9392453	29.1742576	35.9159763	45.7725779	34.4711353
(CGG)n	2237.93179	2323.71254	2248.04783	2193.29092	2097.49128	1982.58123
(CGGA)n	181.120695	182.859761	180.451467	205.206905	250.818772	258.395194
(CGGAA)n	10.5460984	9.9593819	10.1314568	13.7565063	12.84695	9.14447234
(CGGAG)n	40.1775104	49.3068384	41.492498	43.985169	47.3477556	23.2726023
(CGGG)n	198.087457	216.820382	237.457794	226.637213	256.616919	225.194797
(CGGGA)n	21.8203997	27.7554488	29.075732	24.3706734	32.4005687	20.2507917
(CGGGG)n	1446.15131	1493.25106	1440.60111	1258.73647	1293.1616	1217.44505
(CGGGGG)n	218.418946	219.595461	228.701367	292.129959	240.153495	269.307453
(CGGT)n	54.3441772	54.8580091	48.4705005	59.5261896	67.5880842	67.9086536
(CGGTG)n	41.8698351	31.3475494	30.4764752	50.2233883	66.1745571	42.5424705
(CGTAG)n	0.66896111	1.95927243	0	0.80691928	0	0
(CGTG)n	2407.28851	2456.52803	2348.52061	2804.2304	2896.84684	2907.44879

(CGTTG)n	16.4356823	20.572057	7.87898946	17.4497513	15.7153325	19.2962149
(CTA)n	1415.70257	1370.38423	1483.4736	1409.262	1376.44496	1383.28591
(CTAA)n	755.111866	701.316064	765.640066	649.686766	739.186406	658.223675
(CTAAA)n	190.316289	218.287863	176.86394	170.047598	155.315618	178.478338
(CTAAG)n	24.2797231	16.0002189	18.5266235	21.7792399	24.5222383	24.2798726
(CTAAT)n	139.743144	122.287156	125.509849	115.805263	120.915691	102.027648
(CTACT)n	71.8750475	74.776267	72.600097	64.7090566	67.2246756	73.8864081
(CTAG)n	71.5012471	71.8378643	100.422874	81.2665508	85.3635463	101.412039
(CTAGG)n	93.4196844	83.592993	87.8511914	90.1815415	134.933082	129.15149
(CTAGGG)n	251.179167	271.024187	265.534014	324.833907	350.100051	373.988056
(CTAGT)n	43.2339706	61.8783812	49.502855	45.0481018	56.316312	47.6578619
(CTATA)n	414.146166	374.209904	379.725134	354.860596	364.847311	406.773986
(CTATG)n	189.58127	165.06511	168.36312	180.572637	209.99476	199.644186
(CTATT)n	296.157889	325.718772	286.590096	275.999154	256.192739	268.155275
(CTCA)n	1351.46081	1453.57068	1463.11929	1603.36692	1631.64408	1573.64072
(CTCCG)n	76.7480831	93.8794262	84.1158761	98.669843	92.5952743	96.2703006
(CTCG)n	301.77171	334.944463	300.499003	351.885142	349.009214	369.731981
(CTCGG)n	95.3741413	112.98127	89.5546396	77.9146572	57.4478662	63.8795727
(CTCTG)n	726.5164	772.174696	757.894609	926.016389	956.452391	1002.2566
(CTG)n	5151.31821	5180.97376	5161.69334	6377.54508	6331.34705	6565.75363
(CTGGGG)n	1432.65465	1412.59444	1454.76129	1976.28318	2326.51032	2199.32217
(CTGTG)n	2065.63393	2117.33342	2147.2693	2689.94227	3381.31114	3036.72444
(CTTA)n	372.538987	337.63864	395.220378	356.059116	357.069249	341.709302
(CTTAA)n	13.7336247	13.5510778	15.0212013	8.62009849	8.28184649	7.10358513
(CTTAG)n	35.954038	18.9392288	26.6683479	30.9113655	33.8140958	19.2698682
(CTTAT)n	74.2819444	71.0210455	65.5564107	54.2191061	55.4278441	73.0503917
(CTTCG)n	5.27304922	5.5511707	3.50542219	5.47775923	6.10017376	8.11085536
(CTTG)n	1824.81108	1771.37308	1709.77471	1768.75553	1871.15219	1905.69887
(CTTTA)n	15.7667212	16.979754	7.97751507	13.5663913	12.645192	15.1749204
(CTTTG)n	720.26717	689.969091	710.172978	745.838655	731.085654	703.165761
(G)n	745.031314	712.664149	659.778429	883.078504	892.720815	954.47713
(GA)n	63861.5618	62453.8569	64153.175	69224.1979	66108.603	69381.631
(GAA)n	11435.7454	10803.3183	11363.6609	10020.616	9734.17543	9792.5876
(GAAA)n	22730.7143	22030.9712	22793.8742	19069.8967	19068.0207	19064.2559
(GAAAA)n	5750.12385	5619.83136	5725.4637	5214.74705	5304.3374	5235.9432
(GAATG)n	91.0982424	87.3483157	84.2144017	90.9420016	108.148956	84.1962312
(GACTG)n	77.2660562	76.0823475	96.2042235	115.692905	119.945788	122.617407
(GAGAA)n	5287.91526	4458.46715	4385.99362	6780.39958	5256.69696	5814.23765
(GAGTG)n	132.712761	148.083333	129.752048	176.19669	186.382264	151.933631
(GATTG)n	39.5415778	43.1024757	36.1194182	55.5380524	31.2690145	35.4652321
(GCATG)n	76.5908038	96.1643334	97.203736	117.477419	97.685437	102.353442
(GCCTAA)n	89.0127195	97.7971616	84.148718	106.397684	105.037487	145.916356

(GCCTG)n	226.401393	240.24832	249.921659	320.170648	318.00273	328.292041
(GCGTG)n	37.3705999	45.7149402	33.4492992	46.5301433	36.844129	52.7073865
(GCTG)n	540.555174	528.578907	566.751209	708.204551	774.514908	707.843384
(GCTTG)n	111.134571	119.349259	110.324438	144.485985	170.727093	148.938167
(GGA)n	13364.0766	13253.3235	13326.7901	15571.9174	15842.0566	16096.6948
(GGAA)n	13841.2472	13694.6285	13930.7551	14428.4973	13979.9891	14108.9576
(GGAAA)n	2254.61335	2287.54043	2305.12761	2145.36737	2204.6916	2172.81394
(GGAGA)n	2148.55313	2146.23091	2008.61736	2577.33668	2433.75259	2758.54965
(GGAGAA)n	1718.68061	1625.32739	1635.89594	1793.57282	1737.89639	1703.3683
(GGATG)n	431.978488	401.067227	446.238923	522.728289	494.79172	524.575449
(GGCTG)n	652.66645	641.153639	566.187933	818.889055	805.439957	861.406481
(GGGA)n	7097.5861	7009.4039	7035.61099	8257.97311	8603.42376	8626.32841
(GGGAA)n	1225.16976	1185.89285	1230.09362	1430.59988	1474.85539	1463.47941
(GGGAGA)n	3464.56529	3477.92371	3507.34918	4020.68515	3897.61451	4085.38841
(GGGGA)n	1517.66723	1472.67495	1449.47744	1760.12027	1795.66328	1837.49381
(GGGTG)n	1724.78567	1782.39553	1725.08436	2219.82682	2395.5156	2312.34417
(GGTTG)n	273.734061	264.248142	276.092415	301.192396	300.288039	286.496914
(GTATG)n	222.584225	229.961988	217.945915	218.362256	224.357947	261.496045
(GTCTG)n	174.817991	175.349318	148.173018	191.101467	176.382422	183.554209
(GTGTG)n	1036.58511	1001.40229	1049.7921	1166.26975	1228.13804	1188.14151
(GTTTG)n	4053.71294	4059.65246	4076.83619	3794.94961	3875.28249	3814.97234
(T)n	21555.0943	21337.8398	20855.1342	20190.2835	21053.7422	20327.3068
(TA)n	33237.8027	32813.6935	33005.3032	28643.2655	30104.6466	28739.4505
(TAA)n	6974.32477	6716.65539	6923.41736	6208.30099	6395.17166	6288.81261
(TAAA)n	12125.7866	11444.3069	11922.1576	10577.4288	10813.5605	10684.0404
(TAAAA)n	3938.87601	3766.00673	3874.18847	3500.05045	3514.88057	3613.95507
(TAAAAA)n	1116.40224	1102.87184	1072.1975	1011.44078	989.48134	1015.13729
(TAAAG)n	43.0242648	49.633485	32.5483123	37.2349226	36.9656722	36.4856757
(TAAG)n	406.276436	398.209322	414.877882	394.201945	350.302419	368.606151
(TAATG)n	94.9285166	105.634251	96.565484	83.516633	80.4751416	85.1771547
(TACGG)n	9.85092411	6.36748362	4.50493474	10.6995049	9.45350816	11.1326661
(TACTG)n	313.236319	326.207932	317.918295	412.516934	825.276159	619.196524
(TAG)n	1453.27711	1429.16129	1409.22644	1346.51999	1336.04452	1334.20936
(TAGA)n	26612.5215	26776.4181	26577.4243	24476.3824	24957.6042	23977.5798
(TAGG)n	736.347402	723.601953	739.457218	719.470122	780.251063	773.865691
(TAGGG)n	57.7676224	64.8170875	54.9416185	69.4187752	79.3034799	82.6458057
(TAGTG)n	327.455412	329.474499	335.970876	338.76867	336.223646	354.227727
(TATAA)n	1525.55898	1496.34358	1456.73027	1239.75394	1322.10918	1280.47799
(TATAG)n	376.421163	397.311702	354.750942	328.371638	366.826005	328.256061
(TATATG)n	10424.6956	10446.5094	10517.1857	9500.76022	9679.43979	9517.82933
(TATCG)n	5.86337067	5.87761493	4.47209288	7.72784136	8.12019588	12.1926298
(TATG)n	19067.3271	18921.9678	18980.3282	17948.8903	18502.6995	18180.8577

(TATTG)n	260.767959	253.472751	246.434822	239.167691	250.85949	268.63915
(TC)n	64627.4987	64114.6258	65006.1015	70133.6724	67498.0036	70470.4606
(TCC)n	12977.728	12925.3162	13268.1302	15222.3914	15369.9524	15677.8908
(TCCA)n	12310.0686	12104.1603	12266.0477	13739.6053	14433.1017	14380.4077
(TCCC)n	6880.29694	6770.62893	6837.58811	8143.72434	8237.50688	8413.79036
(TCCCC)n	1390.94996	1460.91871	1428.41703	1767.93296	1810.45126	1833.94861
(TCCCG)n	39.6071109	29.8781457	33.3836155	28.4441484	37.8340863	43.5365674
(TCCG)n	257.580433	226.779258	220.911611	276.356155	344.040595	308.23885
(TCCGG)n	10.3888191	5.71439281	13.1863856	18.8929142	16.8869942	15.2144405
(TCCTG)n	371.882608	394.781455	419.69265	461.832416	476.145402	473.293341
(TCG)n	248.805298	220.248349	210.625237	241.604098	224.821624	233.108051
(TCGGG)n	8.59164155	10.122604	9.77948897	14.3927499	28.2389813	13.1208598
(TCGTG)n	2.64963122	3.10202958	2.70296085	8.44942279	4.40345286	7.1167585
(TCTA)n	26800.6223	26792.2574	26941.9634	24496.5037	24516.2938	23895.1985
(TCTCC)n	2259.13722	2179.37421	2238.43102	2711.31279	2609.59051	2783.34633
(TCTCCC)n	3469.65432	3544.37524	3392.58053	4123.07905	3916.03719	4103.67736
(TCTCG)n	16.4356823	11.7554322	10.5491084	12.9495871	21.6137483	19.2435214
(TCTCTG)n	12276.1776	12261.6369	12174.0163	12716.3992	12612.8925	12888.211
(TCTG)n	15133.7078	15143.6371	15017.891	16192.7507	16352.7575	16367.3205
(TCTTG)n	294.955752	274.455595	251.95569	280.11481	227.772053	253.898951
(TG)n	288689.227	289608.601	286511.047	283617.22	286762.417	289187.201
(TGAA)n	6681.63961	6716.50056	6498.98645	6507.59321	6792.15274	6684.2467
(TGAG)n	1482.22435	1456.91684	1438.43851	1568.41289	1633.17976	1620.19251
(TGG)n	8509.87256	8474.41024	8512.23082	9694.62537	10014.4084	10097.4322
(TGGA)n	12572.9662	12534.2906	12635.7762	14016.4981	14436.8409	14562.2165
(TGGG)n	1876.60995	1837.00709	1835.76293	2328.85103	2406.86697	2635.23454
(TGGGGG)n	2660.33826	2601.18238	2565.73191	3293.62656	3476.60225	3481.88411
(TTA)n	6998.95837	6794.53237	6699.35943	6071.87331	6397.69608	6138.8079
(TTAA)n	3500.29756	3492.37585	3424.13429	3165.81985	3211.26292	3204.37713
(TTAAA)n	249.368883	230.860417	255.548181	212.481037	219.651247	203.103765
(TTAAG)n	23.7418281	27.9186709	25.6359935	28.8708377	24.8455395	20.2507917
(TTACG)n	11.1364199	9.30629109	7.14221185	6.83558424	6.90842681	5.06269791
(TTAG)n	664.741302	683.928659	737.014828	649.481002	739.772848	672.476952
(TTAGG)n	47.2477372	40.3273963	45.8660653	48.423225	32.6023267	44.557011
(TTAGGC)n	74.0722387	77.3893385	85.7136705	93.7429895	92.7569249	87.1258283
(TTAGGG)n	3094.18417	3113.92045	3000.80485	6064.12517	5569.43845	6115.25207
(TTATA)n	1505.28569	1447.85254	1537.80548	1248.60682	1284.03139	1196.87456
(TTATG)n	140.228088	148.08394	145.871288	111.960781	121.86371	124.724161
(TTC)n	11306.6637	10845.5228	11117.2347	9812.4796	9754.22856	9797.12995
(TTCA)n	6544.11725	6415.67772	6308.55844	6415.36735	6469.06473	6287.58189
(TTCC)n	14050.555	13790.7158	14026.611	14539.2838	14466.0492	14597.5616
(TTCCC)n	1249.26494	1240.83207	1278.92754	1428.37254	1397.75119	1481.58393

(TTCCG)n	5.91579711	3.59189827	7.07652812	5.39242139	5.73676514	7.1167585
(TTCG)n	87.0844758	92.8990817	95.3453711	71.8125132	110.12826	80.5127049
(TTCGG)n	5.27304922	5.22452412	11.4829373	9.8072478	9.45350816	7.10358513
(TTCGGG)n	41.1222344	54.0414939	35.5140081	49.5482661	50.3777888	29.3820906
(TTCTC)n	5039.19699	4308.42657	4361.15296	6676.15099	5240.03117	5601.996
(TTCTCC)n	1618.87224	1551.61288	1618.36667	1618.03133	1600.13137	1616.87784
(TTCTG)n	313.446025	294.208	305.370162	302.21645	265.766569	268.810404
(TTG)n	15536.3034	15677.929	15590.2815	14272.7774	14424.1994	14040.41
(TTGG)n	5142.80049	5170.77005	5140.35874	5181.50172	5906.51778	5633.5849
(TTGGGG)n	279.610538	257.962371	235.228876	320.341324	333.91921	334.717691
(TTGTG)n	431.086714	461.719722	469.146242	446.035382	505.819795	483.027076
(TTTA)n	12315.7595	12177.7812	12085.9451	10967.3709	11496.5485	11058.1765
(TTTAA)n	244.935705	247.02396	225.228787	199.768024	229.973169	195.628279
(TTTAG)n	154.971446	177.71705	154.913999	123.106415	129.984516	133.671033
(TTTC)n	22844.5693	22511.7086	22751.1465	19126.225	19456.9235	19011.9042
(TTTCC)n	2375.58573	2292.60173	2395.42832	2252.05237	2255.3532	2362.21771
(TTTCG)n	28.3721294	19.4286927	31.6801672	18.8929142	18.9070163	15.2012671
(TTTG)n	36631.4896	36605.3164	36361.3642	33659.5266	34780.9648	34158.3287
(TTTTA)n	4083.02507	3936.61937	3976.35756	3479.93909	3888.7335	3636.10234
(TTTTC)n	5888.59356	5728.97408	5739.2998	5074.76881	5386.97111	5130.4423
(TTTTG)n	29099.4531	29276.9819	29174.4596	26517.4112	27175.1566	26405.3952
(TTTTTA)n	1145.59537	1132.25901	1120.95861	1035.21029	1015.63831	1021.55635
(TTTTTG)n	12103.7644	12170.2922	12059.1376	10994.6019	11099.2404	10835.0965
4.5SRNA	2871.11291	2818.16477	2760.69553	2930.75412	3179.5059	3132.7353
5S	3197.97957	3266.66016	3173.23884	3341.23383	3497.16344	3493.16473
7SK	3799.7536	3722.33733	3710.34174	3996.30213	4118.68295	4104.82954
7SLRNA	1710.93461	1724.92303	1742.61643	1858.71615	1982.28924	1967.07649
A-rich	52368.8614	51935.8739	52040.3722	47036.1399	48011.1187	47253.2575
AmnSINE1	5442.03319	5427.83187	5560.88453	5610.77861	5628.86745	5615.45049
AmnSINE2	1559.39185	1541.24554	1607.08078	1673.02606	1618.93812	1671.88299
Arthur1	6402.18687	6335.92591	6317.05927	5947.76607	6163.33584	5896.32533
Arthur1A	669.843966	675.600992	679.107514	691.999779	748.638083	648.019239
Arthur1B	3657.49975	3697.92884	3659.10756	3600.02408	3570.99513	3438.60442
Arthur1C	832.075946	792.992194	788.460317	795.841118	828.427736	745.826792
AT_rich	328727.029	326208.432	327586.503	289587.131	297161.115	285932.494
B1_Mm	133894.724	132636.691	130318.174	142867.875	151691.166	149277.371
B1_Mur1	152674.901	153782.358	150326.528	160715.749	177245.482	171281.904
B1_Mur2	125623.153	125857.046	123047.28	131358.743	142533.155	138200.099
B1_Mur3	96256.5082	97036.8885	94666.24	103479.012	111840.788	108736.109
B1_Mur4	153240.207	154598.289	150959.585	164198.399	184958.676	176622.439
B1_Mus1	331810.627	331549.282	327140.301	359679.078	391468.319	383163.397
B1_Mus2	237376.1	237138.152	232035.318	257523.033	279977.621	271865.879

B1F	172680.055	172749.692	169761.511	181389.978	194485.284	187942.683
B1F1	75855.0981	76462.3288	74806.0417	80704.0085	85861.2124	82811.202
B1F2	63123.3098	63102.6925	61946.4621	66647.8451	72005.9737	70084.324
B2_Mm1a	57353.4254	56654.8755	55523.5747	58724.4468	64411.0706	62154.9861
B2_Mm1t	85137.6035	84545.8241	82739.1168	87015.7458	95637.8216	92208.3115
B2_Mm2	371417.931	369900.545	362751.927	380164.29	415380.181	400469.964
B3	735917.978	736647.774	725063.657	764413.788	820226.593	798661.478
B3A	422290.077	422178.901	418350.482	439980.68	462059.089	455390.135
B4	318911.635	321027.284	316639.529	342992.955	352595.02	351959.353
B4A	571634.181	575313.028	571840.063	616642.968	636079.87	631647.485
BC1_Mm	18476.0346	18362.6305	18279.0061	18898.0027	20054.1151	19468.1561
BGLII	17294.6352	17174.0286	17265.0385	16767.9042	15663.7629	16324.7016
BGLII_A	1781.14983	1787.78196	1764.3058	1854.37859	1730.13838	1923.37879
BGLII_B	8475.96263	8453.26003	8643.84837	8909.4027	8776.01533	9091.03764
BGLII_B2	2499.46255	2637.42427	2469.63334	2555.23553	2577.98119	2730.63894
BGLII_C	438.970075	407.923871	471.072455	437.419074	450.713421	463.928461
BGLII_Mur	5115.29392	5132.56465	5298.02279	5377.81503	5527.10247	5509.14347
BGLII_Mus	4324.94783	4240.14084	4397.24181	4383.13275	4100.8686	4243.49131
BLACKJACK	5040.35612	5076.80699	5095.50644	4577.12617	4510.8656	4476.85014
C-rich	42467.4271	42614.066	42166.7734	49581.6944	49998.8746	51545.172
C573_MM	753.688489	761.562831	788.016952	706.178696	583.386447	684.133013
Chap1_Mam	832.240565	833.808042	922.158988	826.752483	870.120162	861.633475
Charlie1	6816.14902	6837.89222	6903.66999	6422.46983	6882.604	6560.81962
Charlie10	2040.75078	2090.31702	2067.07586	1928.56443	1959.10337	1933.55029
Charlie10a	325.559673	305.474171	307.51914	311.806563	321.982002	273.152106
Charlie10b	240.397149	238.125117	239.405392	233.597014	237.426709	250.959228
Charlie11	313.596489	288.656627	322.883016	296.358461	277.179328	273.952142
Charlie12	4783.5301	4816.31235	4681.95929	4284.75633	4583.76322	4317.17707
Charlie13a	1212.78716	1247.69144	1253.91121	1116.46168	1142.69306	1148.69684
Charlie13b	791.754263	798.540734	748.736951	750.695819	760.699276	727.021039
Charlie14a	351.584147	335.923796	394.385075	302.984491	330.567707	311.593042
Charlie15a	1041.67362	1006.95477	1017.24162	1037.58459	1034.46389	1050.55996
Charlie15b	1667.406	1669.41163	1683.77033	1503.56775	1545.63821	1493.85255
Charlie16a	1060.51725	1088.42355	1126.88071	1054.54506	1089.99322	1128.1233
Charlie17	859.143706	794.37857	792.019966	846.975715	819.09455	771.015134
Charlie17a	284.509787	334.372377	322.56389	313.164388	330.385392	358.13166
Charlie17b	826.691229	897.970521	903.862258	856.286096	834.388754	862.433511
Charlie18a	1226.93967	1234.79285	1189.08662	1244.80122	1225.39303	1177.19986
Charlie19a	1531.57125	1483.85648	1465.91365	1453.2325	1567.06903	1565.18736
Charlie1a	18654.7201	18560.2604	18439.8265	17207.624	18213.6371	17358.8179
Charlie1b	5196.26443	5209.29836	5185.13389	4920.4263	5266.97667	4949.53446
Charlie1b_Mars	17.1308567	24.3267726	36.0865763	22.7179562	30.9056058	20.2903118

Charlie20a	1078.56609	1048.83116	981.277123	996.850819	1029.59676	994.13611
Charlie21a	1094.20909	1064.91309	1135.78276	990.569443	986.833548	929.381001
Charlie22a	1042.99215	1041.32183	943.70057	1021.75247	1057.51244	920.052102
Charlie23a	790.062462	839.846957	841.384325	818.702242	838.385639	807.056455
Charlie24	2383.61222	2406.97241	2494.07493	2252.55254	2282.1361	2206.37307
Charlie25	2115.94547	2161.90894	2203.18408	1899.37975	1905.02767	1807.44037
Charlie26a	550.379885	489.068734	504.465363	532.550698	501.173256	465.001598
Charlie29a	1985.38324	2119.05018	1922.58673	1878.15899	1947.79393	1875.08861
Charlie2a	3144.48313	3161.67415	3073.6491	2877.4571	2941.75711	2800.17097
Charlie2b	5168.71382	5215.09245	5069.14576	4539.49206	4910.13351	4807.18003
Charlie4	749.721381	769.969579	754.624044	702.319053	728.299317	698.402511
Charlie4a	6503.44326	6487.5262	6357.29232	6184.12141	6437.77078	6258.52255
Charlie4z	1854.24484	1923.37465	1923.36781	1972.60033	2065.7592	2073.02478
Charlie5	7351.95069	7306.46899	7196.15151	6769.77705	7011.68732	6761.09967
Charlie6	1011.9641	968.666539	1036.27513	1020.91046	905.124752	883.168178
Charlie7	5360.33343	5465.79133	5478.18581	4943.13765	5032.96712	5055.15038
Charlie7a	449.5686	451.354308	443.855088	426.106555	495.335612	398.808037
Charlie8	4199.61566	4283.49129	4105.78056	4359.78234	4355.26557	4392.84443
Charlie9	1022.52382	1058.70923	1043.18962	985.21211	985.540954	976.587921
Cheshire	6116.18608	6159.51514	6105.54017	5725.12788	5890.88632	5755.07697
Cheshire_Mars	16.9211509	12.5715428	12.4824498	8.91499068	15.7153325	10.164916
Cheshire_Mars_	40.0464443	37.8780528	38.1512852	32.7812175	41.8941843	28.3879938
CR1_Mam	1520.81544	1511.69586	1455.52658	1495.99922	1540.06065	1482.0217
CT-rich	68415.6262	67636.6642	68772.3557	72674.8237	72088.575	73526.4018
CYRA11_Mm	3001.1451	3195.71911	3076.47414	3066.27816	3051.19744	3084.52465
DNA1_Mam	208.069971	200.655828	198.945249	195.981861	199.491133	203.712787
ERV3-16A3_I-int	7292.81579	7247.37095	7154.24849	7000.34078	6966.38749	6875.08184
ERV3-16A3_LTR	1752.90405	1744.67837	1776.52056	1823.94417	1774.45794	1817.6281
ERVB2_1-I_MM-int	925.738365	965.400984	956.159471	843.371598	770.718561	801.424255
ERVB2_1A-I_MM-int	2988.58583	2922.32537	2931.59307	2786.29418	2588.38394	2569.33738
ERVB3_1-I_MM-int	2011.28504	1963.04754	2025.32343	1712.62293	1672.30888	1721.21289
ERVB3_1-LTR_MM	1158.79686	1145.40335	1177.29881	1115.66993	1173.11371	1219.34713
ERVB4_1-I_MM-int	6757.13418	6681.24378	6768.84044	6866.27652	6373.93771	6743.0565
ERVB4_1-LTR_MM	93.8003001	96.6546068	84.2872137	81.7321187	78.5158911	91.7507579
ERVB4_1B-I_MM-int	12935.4246	12795.3633	12891.193	11631.3969	11488.4045	11525.7327
ERVB4_1B-LTR_MM	6112.50522	6041.96496	6270.58779	6499.02959	6180.23496	6699.08825
ERVB4_1C-LTR_Mm	4302.89256	4198.02175	4169.97836	4509.75415	4399.90354	4568.61004
ERVB4_2-I_MM-int	9105.64481	9140.9516	9219.64725	9757.79076	9634.00925	9878.64894
ERVB4_2-LTR_MM	820.22443	790.706376	790.853445	862.862365	897.084771	929.87805
ERVB4_3-I_MM-int	2921.58854	2856.94166	2925.99442	2931.95264	2755.69387	2965.27107
ERVB4_3-LTR_MM	1083.6352	1176.18063	1087.44859	990.864823	829.476023	929.506148

ERVB5_1-I_MM-int	4474.79197	4424.88343	4552.01885	4388.65696	4383.31857	4459.63384
ERVB5_1-LTR_MM	1263.35978	1218.71274	1260.88705	1287.08342	1206.94969	1207.58568
ERVB5_2-LTR_MM	2363.42962	2287.38297	2267.48537	2275.82947	2241.8171	2292.91976
ERVB7_1-LTR_MM	4246.36325	4212.63245	4299.08936	4687.76665	4457.62972	4694.9966
ERVB7_2-LTR_MM	2736.54687	2735.55192	2791.38547	2999.38969	2944.053	3175.34718
ERVB7_2B-LTR_MM	3133.41644	3084.3737	3177.85159	3418.3577	3401.09146	3531.54993
ERVB7_3-LTR_MM	2246.59735	2247.70624	2238.39818	2228.48152	2129.26163	2318.32192
ERVB7_4-LTR_MM	1295.0374	1226.38681	1233.14142	1367.01158	1296.57448	1272.71269
ERVL-B4-int	11656.1728	11724.9091	11651.6712	11170.1628	10851.9792	10919.8019
ERVL-E-int	7851.81405	7734.80392	7878.57605	7580.04698	7643.02008	7567.73104
ERVL-int	5855.84592	5969.63814	5910.79652	5520.44914	5428.20526	5498.21194
ETnERV-int	20029.4331	20110.6624	20240.7623	19819.8782	18744.8918	19829.4109
ETnERV2-int	19673.0467	19410.468	19642.7312	17335.6212	16609.3046	16501.104
ETnERV3-int	11896.0341	11761.3075	11789.5114	10273.7193	15572.0458	12527.2495
Eulor1	303.464034	294.045587	291.03864	274.152532	224.862953	235.59988
Eulor10	165.091841	155.593776	134.749611	139.093564	120.955799	153.019941
Eulor11	289.756623	264.003916	244.304429	270.641821	259.525409	251.920391
Eulor12	162.547063	166.043026	160.72828	140.110038	158.808718	175.35114
Eulor2A	250.588845	226.615428	275.08361	209.909043	233.99316	243.842469
Eulor2B	215.487261	211.186183	197.347454	203.41481	194.684164	228.568749
Eulor2C	100.227779	116.409946	95.8029928	106.358806	96.3521247	101.412039
Eulor3	146.727915	150.450964	153.61891	138.83755	135.377926	145.96905
Eulor4	153.11555	144.410329	142.562917	118.501473	115.299248	133.072137
Eulor5A	420.612964	401.637796	414.713673	391.459275	375.975655	367.931262
Eulor5B	163.268451	172.084066	176.110742	159.685655	149.234887	155.192562
Eulor6A	104.372612	95.8382939	100.202274	99.6474379	85.1617883	92.8173083
Eulor6B	335.089747	291.595637	289.579341	294.527487	298.874512	273.715022
Eulor6C	65.4019575	67.5923693	54.7117255	49.9749553	51.7311548	61.8386854
Eulor6D	253.4356	261.391148	244.435797	239.462583	234.355347	226.09668
Eulor6E	88.3699716	97.6337371	96.4341166	102.619101	95.7869581	85.190328
Eulor7	22.836948	19.9185614	17.7898459	12.1426678	7.91843787	15.267134
Eulor8	632.919514	624.661427	644.93073	559.869806	541.753571	550.372814
Eulor9A	314.495601	309.554926	296.083301	295.714637	281.461238	247.460129
Eulor9B	64.1164617	55.184251	53.7122129	48.9120225	60.3563562	52.7073865
Eulor9C	289.704196	310.53608	267.237462	256.353848	315.619909	262.111654
FAM	317.728739	300.412262	275.813259	319.076417	353.677028	380.933561
FordPrefect	2728.05956	2660.60757	2769.73607	2857.61789	3495.04193	3126.01375
FordPrefect_a	373.673494	337.882259	340.067452	353.813312	374.864765	396.556376
G-rich	43352.4931	43583.8774	43522.3812	51506.487	51815.894	52825.0455
GA-rich	67908.0221	66612.8146	67643.2046	71349.4827	71500.2333	72154.1263
GC_rich	17109.6543	17941.9403	17068.2642	17056.6889	16810.8704	16129.5606
GSAT_MM	2759019.42	2747142.67	2770792.3	2042989.43	10889452.3	6079181.55

HAL1	22242.5677	22490.6875	22145.1598	21202.9281	22353.1498	21578.3753
HAL1_SS	230.900104	245.309318	244.581421	204.807235	217.267816	206.33635
HAL1b	3918.47689	3895.2372	3882.89564	3418.12822	3399.58072	3462.08121
HAL1M8	4368.8581	4282.92163	4430.01506	4065.02916	4127.17083	4094.55009
HAL1ME	5215.62655	5263.74721	5074.67159	4636.20293	4825.93979	4689.7074
hAT-N1_Mam	1000.65781	989.075475	972.909834	910.567799	903.691782	905.091002
Helitron1Na_Mam	414.336473	398.04782	372.090295	366.715952	368.037774	373.96171
Helitron1Nb_Mam	547.520548	558.374632	545.136815	510.814127	539.553676	484.093626
Helitron2Na_Mam	395.978839	387.107689	389.183333	395.835223	364.341085	347.433716
Helitron3Na_Mam	1089.03355	1106.95503	1070.69327	1056.62495	1049.83525	1082.8914
HERV16-int	1905.72339	1872.68012	1956.57079	1951.63229	2007.45992	1841.23003
HERVL32-int	2.00688333	7.34701866	7.07652812	2.25008213	4.56510347	0
HERVL40-int	1298.3497	1356.9992	1345.45559	1286.5676	1233.12793	1322.12212
HERVL74-int	1089.44615	1126.05677	1137.95313	1076.80979	1040.68377	998.902407
HUERS-P3b-int	12.3694892	21.5511873	16.8231752	18.5126841	13.6953104	17.2158076
HY1	103.834717	122.614612	114.993582	131.831291	144.084564	133.829113
HY3	34.878248	36.4088514	32.5811541	35.8770976	22.1388075	28.4011671
HY4	9.82471089	20.8980965	13.2520693	8.44942279	7.27183543	6.06996815
HY5	1.33792222	6.69392785	6.20838304	2.33541998	5.41346392	2.01454047
IAP-d-int	11201.4014	11139.0177	11581.1538	12041.112	11351.7637	11762.0961
IAP1-MM_I-int	7603.79515	7716.11145	7730.30947	7716.93032	7290.75719	7861.7232
IAP1-MM_LTR	2506.48716	2452.28294	2526.78312	2656.85381	2495.52125	2709.64435
IAPA_MM-int	403.075279	406.863028	379.152566	408.125337	453.784782	437.456208
IAPEy-int	11756.9925	11719.2776	12307.9857	12027.8077	10774.7267	12004.181
IAPEY_LTR	4334.5167	4377.69635	4484.2171	4841.99068	4400.5076	4914.97062
IAPEY2_LTR	7889.75925	7846.40357	8131.05505	8571.53961	8088.08724	8623.08315
IAPEY3-int	22886.2179	22825.0828	23277.4117	23467.6206	22786.0561	23947.6911
IAPEY3_LTR	3804.26332	3821.03656	3905.54888	4199.30211	4060.10597	4230.95617
IAPEY3C_LTR	2162.37116	2176.11422	2212.55801	2312.09535	2317.66575	2453.06304
IAPEY4_I-int	9774.55506	9802.02026	10047.1149	10119.5723	12694.3484	11603.057
IAPEY4_LTR	707.844207	756.091956	706.04356	670.593188	741.74971	698.139043
IAPEY5_I-int	1764.22292	1790.14726	1886.23462	1851.32159	1666.02456	1804.30305
IAPEY5_LTR	1331.97391	1282.38768	1360.82163	1415.63678	1312.04551	1439.46301
IAPEz-int	206413.378	206486.752	208177.452	218621.469	262259.465	244484.484
IAPLTR1_Mm	7963.70147	7971.05259	8106.29496	8556.78888	10246.9363	9503.66679
IAPLTR1a_Mm	9223.66979	9260.2153	9454.36084	10166.6329	13198.4034	12021.0695
IAPLTR2_Mm	8367.68897	8448.04674	8406.17863	9547.60578	9248.55637	9681.55026
IAPLTR2a	3954.19292	4005.60884	3972.7223	4411.33653	4089.65821	4392.18272
IAPLTR2a2_Mm	9999.88171	9780.54795	10251.5389	10314.5026	10191.9181	10709.0039
IAPLTR2b	6153.9179	6091.03294	6118.36746	6608.5492	6173.52829	6602.10758
IAPLTR3	1197.99085	1170.87389	1234.75563	1348.44058	1265.76793	1424.44567
IAPLTR3-int	10268.5163	10120.9682	10431.1095	10434.0897	10907.6774	10869.1775

IAPLTR4	2677.92522	2629.83444	2740.68173	2771.68527	2592.21795	2767.63484
IAPLTR4_I	1686.58306	1697.57665	1737.17054	1879.514	1792.67519	1789.39818
ID	19524.3119	19592.0386	19057.4813	20577.1053	22122.3198	21660.9655
ID_B1	463797.551	466204.572	456606.072	501254.412	539486.815	528760.415
ID2	17075.4251	17334.0461	16773.0649	17874.1002	18993.3412	18492.8708
ID4	75382.3764	75273.9817	73812.0637	79252.871	85005.7856	82679.2597
ID4_	80997.9322	81548.7635	79905.7446	84411.962	92705.0404	89094.0587
IMPB_01	84494.0608	84504.2087	84666.0481	86708.9317	86566.2557	87643.5858
Kanga1	1910.2955	1878.88297	1812.7856	1730.21255	1744.11872	1772.88361
Kanga11a	1167.572	1174.71082	1180.01106	1093.02924	1119.68828	1134.02861
Kanga1a	1132.26123	1200.26125	1137.50047	1114.98343	1071.89324	1065.84331
Kanga1b	680.049292	732.256064	728.251273	699.29714	703.83663	659.629194
Kanga1c	1131.6447	1120.83346	1096.58983	968.832872	1011.47611	964.747433
Kanga1d	666.17831	664.580363	646.962597	628.376884	616.855967	627.781621
Kanga2_a	2320.79593	2291.29929	2228.88639	2095.67545	2167.38014	2129.64927
L1_Mm	52680.4841	53255.4621	54360.6749	47912.7996	43408.1839	45924.2904
L1_Mur1	84052.6518	83282.691	85134.824	73714.9413	66843.4189	70676.2258
L1_Mur2	214493.477	210534.802	216850.018	189301.13	172436.123	180670.011
L1_Mur3	253479.481	251861.342	252564.34	219817.846	210868.999	210389.274
L1_Mus1	433419.745	428634.221	436414.315	424445.191	404998.277	419714.743
L1_Mus2	311787.621	307698.79	314523.967	298134.641	281839.132	295327.559
L1_Mus3	643796.157	635634.268	650154.953	605063.605	562698.917	590669.101
L1_Mus4	184189.185	182208.855	186367.172	179795.732	168623.951	176806.503
L1_Rod	89882.5419	88895.6578	89337.8932	77232.8395	75372.5123	73924.3462
L1M	1572.81825	1566.22571	1617.98402	1514.30235	1490.93718	1472.13696
L1M1	3623.19767	3591.722	3551.72311	3338.04882	3305.48926	3165.14274
L1M2	120694.415	119924.399	120328.161	105622.677	103698.887	101414.98
L1M2a	1415.92538	1410.38477	1394.33445	1360.62971	1350.46726	1391.88064
L1M2a1	51.9828914	48.8171721	45.8003816	35.9624355	41.6109905	46.7032852
L1M2b	62.2668577	75.5928835	65.3922014	89.9644066	95.4636569	76.0853758
L1M2c	2362.9085	2284.92989	2443.14346	2006.73559	2092.56338	2057.0336
L1M3	41145.8095	40799.1741	41269.421	36734.6737	37374.4049	35795.2243
L1M3a	725.53288	796.744683	757.317712	638.863045	625.441671	638.035211
L1M3b	1258.74259	1258.87398	1260.19241	1051.39135	1147.17917	1107.17786
L1M3c	2356.63673	2308.27783	2299.86515	2048.7229	1944.58098	1939.21543
L1M3d	1115.94456	1095.36312	1036.10379	962.9986	1005.27811	932.290838
L1M3de	1155.65547	1173.73078	1195.01368	1112.08146	1098.33706	1076.06751
L1M3e	4501.32131	4581.93699	4286.68748	4377.41841	4345.7689	4274.84232
L1M3f	610.830167	599.516925	578.356221	577.051685	554.178172	542.917087
L1M4	83025.2209	82796.9788	81939.9574	75385.6498	78029.8182	75144.3965
L1M4a1	4013.65548	3908.70525	3739.2133	3594.77813	3570.32908	3517.77699
L1M4a2	4472.20315	4588.95949	4439.14204	4066.87578	4122.94908	4064.87514

L1M4b	15466.4106	15320.5379	15225.2894	14037.2707	14194.8978	13897.2894
L1M4c	14561.1149	14310.1581	14346.9959	12794.5034	12791.6894	12436.6905
L1M5	94713.1027	94768.82	93355.276	85187.0192	88445.5821	84734.7771
L1M6	1986.70858	1949.00751	1901.05228	1757.62127	1751.41061	1640.57199
L1M6B	239.649549	267.595713	221.279999	213.183179	251.727293	213.86453
L1M7	1300.33719	1333.24351	1288.69774	1279.69693	1191.83963	1143.64378
L1M8	1386.07692	1412.83503	1392.75027	1258.38705	1348.48918	1244.36471
L1MA10	4622.44574	4678.26839	4626.22017	4201.72092	4049.70322	4080.67884
L1MA4	54236.9191	53988.1766	54155.8364	48876.3499	48857.4489	47963.5498
L1MA4A	19782.1241	20041.0913	19770.2971	17768.6719	17845.8242	17628.2226
L1MA5	32196.6004	31976.199	32164.3504	29340.856	29130.2649	28471.3742
L1MA5A	9641.86013	9638.25516	9783.13782	8850.20735	9303.85666	8730.36146
L1MA6	46924.1121	46192.6375	46303.6755	42062.4744	41522.8541	40894.7634
L1MA7	20771.1831	20777.5134	20950.3711	18827.9229	18792.6888	18261.0259
L1MA8	25647.2757	25653.6589	25196.4891	22935.009	23008.7211	22302.7931
L1MA9	29725.0812	29531.4235	29675.985	26512.7707	25925.502	25707.6661
L1MB1	16971.2308	16852.5579	16752.3679	15208.5143	15449.8005	15301.2604
L1MB2	20225.5116	20382.8159	19968.3807	19627.9072	20315.9603	19862.8129
L1MB3	37898.3001	38200.5514	37513.0607	36934.7752	39303.4462	37305.4866
L1MB4	17680.5025	17819.0282	17584.8895	16924.4095	18061.0653	16952.6524
L1MB5	26286.9037	26323.9711	25903.1783	25604.5882	26820.7667	25596.8953
L1MB7	49377.6234	49360.5314	48290.997	46438.5972	49060.0023	47049.1141
L1MB8	40865.2454	41145.7075	40453.9679	38796.5943	41196.6516	38687.7079
L1MC	31381.5563	31308.75	31082.659	27689.227	28238.4878	27405.1762
L1MC1	42200.3203	41669.6377	41767.8902	39018.2051	38765.6871	38234.8225
L1MC2	16263.0898	16223.0823	16331.8899	15242.2632	15137.8007	14834.1687
L1MC3	38985.6177	38643.0728	38736.8217	36179.2241	35839.9479	35788.066
L1MC4	35167.3996	35100.9071	34838.2623	33414.6039	35300.1761	33694.5609
L1MC4a	19483.8791	19246.4802	18985.3602	18270.3344	19119.5987	18376.526
L1MC5	21540.7232	21444.9588	20986.8561	19818.2936	20742.4328	19852.8795
L1MC5a	14617.7207	14787.6339	14526.3586	14044.0494	14688.9639	13907.3895
L1MCa	23387.1198	23274.7703	23474.5962	21262.9411	20955.6381	20715.6915
L1MCb	5076.42864	5311.66443	5199.78734	4700.78923	4532.69567	4478.51864
L1MCc	3244.62493	3240.37513	3293.81897	2993.91426	2953.34669	2875.64073
L1MD	32431.2553	32700.1232	32094.2407	28985.1974	30034.4185	28764.4923
L1Md_A	254286.176	251829.315	257524.926	251487.618	228527.615	244617.897
L1Md_F	95116.8224	95477.318	96797.0413	91177.4938	82487.9587	87205.2635
L1Md_F2	1292547.55	1276865.53	1308203.84	1235878.77	1139429.09	1203376.84
L1Md_F3	233058.427	230150.553	235363.729	222588.147	204356.672	217629.596
L1Md_Gf	18701.4268	19013.756	18876.7835	18894.9813	16891.041	17844.8678
L1Md_T	452617.194	444717.911	456943.02	437886.064	401730.376	426024.279
L1MD1	16351.3188	16453.0524	16302.6115	15161.4569	15723.1239	15160.3444

L1MD2	20751.2715	20786.8222	20793.4773	19450.0966	20278.8524	19414.1587
L1MD3	13276.8532	13605.0846	13106.8244	12420.0449	12643.4383	12318.2405
L1MDa	22878.6564	22754.1119	22864.8532	20885.3294	20928.5951	20373.3038
L1MDb	3501.9967	3316.86209	3392.63476	3135.15361	3189.89042	3095.50278
L1ME1	38403.0113	38053.1038	37727.0647	34949.9918	36165.7536	34586.875
L1ME2	18434.6775	18746.2241	18724.5182	16950.6561	17632.1127	16813.376
L1ME2z	9643.44026	9610.33892	9629.37914	9003.25414	9516.64142	8715.44342
L1ME3	9777.53708	10026.5096	9970.92295	9402.24356	9631.6818	9221.83128
L1ME3A	13497.3687	13689.6631	13084.9495	12623.9438	12793.6674	12532.2687
L1ME3B	4472.87474	4514.10455	4490.64888	4159.32587	4454.6119	4111.34484
L1ME3C	2003.78701	2022.4786	1938.30754	1849.73428	1896.36358	1848.10308
L1ME3Cz	5924.4417	5953.79892	5918.4227	5498.13794	5601.84817	5472.63853
L1ME3D	2062.32268	2129.41802	1990.40274	1904.06575	2067.87704	1977.07369
L1ME3E	2024.34865	1949.98603	2031.8531	1834.01929	1868.74993	1787.18958
L1ME3F	1931.14496	1981.33358	1958.05644	1804.60843	1964.80003	1826.42043
L1ME3G	6182.48872	6075.67539	6021.09867	5785.33347	5847.37502	5670.69935
L1ME4a	8774.94001	8690.81498	8491.88581	7846.3493	8342.05158	7848.2735
L1ME4b	10162.2096	10353.0421	10174.8636	9810.10103	10329.1089	9793.48595
L1ME4c	5456.70996	5462.11691	5405.46148	5046.20239	5292.12851	5125.22457
L1ME5	1066.2894	1151.11895	1122.16231	1068.85296	1111.78745	1051.57381
L1MEa	1129.64411	1115.60762	1117.6995	988.544564	1026.38441	1043.79534
L1MEb	2190.99389	2203.62342	2140.0679	1956.13625	1996.65426	1951.72422
L1MEc	37401.7045	37069.4857	36672.8885	33616.6579	34710.8032	33547.5628
L1MEd	18322.2895	18246.6199	18462.1141	16623.3721	17275.6967	16513.8246
L1MEf	20084.4143	20001.2576	19777.1803	17819.2963	18136.062	17312.605
L1MEg	20733.7301	20715.7071	20374.4907	18368.1964	18744.5012	18114.9733
L1MEg1	585.868376	578.212392	575.803213	585.959095	560.742023	493.992028
L1MEg2	739.325223	685.724204	679.313858	684.349694	668.710496	631.333413
L1MEh	1163.06018	1207.28132	1142.11322	1057.33089	1004.166	1002.30625
L1MEi	2819.47498	2890.56987	2905.49016	2687.59682	2686.77614	2577.44165
L1MEj	1135.84248	1132.6704	1119.39366	1045.70831	1047.29139	983.408279
L1P5	613.880336	630.866194	559.782499	581.827302	564.116022	567.289174
L1PB4	1171.02113	1127.20054	1169.10782	1085.61573	1011.71919	1017.49434
L1VL1	39111.2739	38616.7944	39603.6271	35153.3788	33056.4391	34448.8492
L1VL2	95931.2112	95076.3416	96354.6639	87310.9647	84409.4883	85392.658
L1VL4	129186.337	127444.764	130159.061	115398.678	109526.861	111389.816
L2	108598.444	108472.246	107574.179	109463.323	112152.374	111111.05
L2a	137265.249	137809.3	136138.759	138803.732	144312.323	141747.233
L2b	68284.7379	69120.5889	68251.6424	73502.3798	76734.3058	76484.4273
L2c	60975.0901	61234.0952	60196.7431	62967.6814	65335.077	64119.1536
L3	46547.5126	46479.4541	46728.089	45960.8679	47145.4183	46989.7404
L3b	9890.10656	9852.79357	9781.11894	9922.28615	10127.4361	10111.1586

L4_A_Mam	3877.22359	3995.16151	3975.60806	3803.90018	3783.8996	3701.84191
L4_B_Mam	2099.35723	2146.07154	2027.82221	2056.81202	2134.21386	2062.35623
L4_C_Mam	3438.4627	3384.12933	3384.56305	3254.16663	3325.66759	3286.0486
L5	713.235215	723.438933	745.630595	707.46353	719.491801	698.731845
LFSINE_Vert	5444.57325	5259.18022	5564.34998	5569.10233	5511.8502	5697.53692
Looper	1183.6019	1156.26257	1187.70942	1139.28014	1357.3763	1225.40746
LSU-rRNA_Hsa	6153.03609	6252.42768	6362.51201	7059.55264	10486.1874	8278.39509
LTR10_RN	3542.98525	3513.27576	3624.18191	3778.19589	3812.68186	3608.79407
LTR101_Mam	1090.27291	1118.05636	1162.89944	1093.8869	1013.21233	1065.71512
LTR102_Mam	1304.69854	1398.55097	1413.47081	1403.23052	1347.88329	1341.91184
LTR103_Mam	353.859978	387.351409	349.354313	359.76043	366.179402	340.734965
LTR103b_Mam	303.024177	297.473454	309.171161	285.146929	285.946126	317.768398
LTR104_Mam	1484.1332	1528.34978	1545.87159	1526.46873	1591.42901	1531.77314
LTR105_Mam	821.228396	804.25715	779.154871	821.845069	892.15748	796.878366
LTR106_Mam	1007.1178	1001.15664	971.905993	899.410306	866.808156	845.793792
LTR107_Mam	566.022355	528.579211	554.048159	552.076066	517.37293	490.420475
LTR108a_Mam	237.983437	216.412023	261.249679	230.376917	220.155642	237.719808
LTR108b_Mam	125.97649	129.960822	140.432524	137.68928	130.651172	147.90455
LTR108c_Mam	114.433242	108.899301	106.377815	113.279728	95.3822211	96.283474
LTR108d_Mam	190.939114	194.288546	161.915551	199.779883	164.425161	150.580806
LTR108e_Mam	220.669088	223.840551	204.989422	179.261272	178.342893	191.796798
LTR109A2	184.924756	168.0024	173.473465	165.392408	170.727093	166.285708
LTR16A	9614.93916	9845.85825	9760.05853	10223.4667	10115.9559	10362.8545
LTR16A1	3989.23526	4068.87532	4140.24299	4301.15647	4063.29765	4240.0682
LTR16A2	2585.71292	2645.42549	2572.38862	2608.19352	2519.58409	2603.90683
LTR16B	1813.56928	1730.88135	1866.24653	1776.11452	1731.77433	1766.42503
LTR16B1	2045.14464	2056.35448	2124.0286	2161.33227	2160.59203	2119.13526
LTR16B2	2656.17874	2621.26113	2608.97712	2676.57259	2669.82837	2664.68909
LTR16C	8379.62017	8404.77082	8580.49299	8961.86712	8666.20548	8702.86876
LTR16D	1177.47482	1271.77166	1159.49471	1267.85722	1196.02311	1224.66012
LTR16D1	537.13802	519.273122	548.339532	562.620544	609.504527	542.857807
LTR16D2	354.12211	366.454223	325.431061	360.784484	322.587276	335.48784
LTR16E1	3176.52773	3119.63828	3108.1165	3396.02328	3218.71157	3248.62811
LTR16E2	2659.78568	2769.59102	2693.37928	2662.0324	2619.85288	2698.44227
LTR23-int	26.9031411	37.5514062	40.6900367	34.9848405	30.7840626	34.497482
LTR27C	41.3057269	30.5308318	41.5253399	37.0642469	24.6838889	32.4170747
LTR28	50.0546477	55.0208265	53.9092641	55.8329446	71.8298865	60.8050685
LTR28B	386.692023	432.413663	408.446734	438.990243	387.671607	412.587073
LTR29	188.276377	176.655499	213.032621	188.339357	171.98019	218.887708
LTR31	1219.20783	1252.26156	1214.68977	1251.3111	1242.33958	1292.78918
LTR32	33.33062	27.5922267	40.0189428	26.9621069	37.3691882	32.4302481
LTR33	9222.41889	9266.01282	9277.40678	9833.71563	9811.12765	9819.97393

LTR33A	3345.64015	3303.96775	3444.92484	3477.73644	3565.94385	3704.14931
LTR33A_	3019.12946	3085.67675	3098.52477	3266.65309	3292.07287	3180.17276
LTR33B	1589.11658	1586.96281	1596.27823	1572.99839	1511.35555	1491.59735
LTR33C	1442.09927	1472.75464	1461.07533	1494.66083	1526.76947	1556.0069
LTR34	20.987344	27.2653777	18.6251491	19.0635899	28.5622825	17.2553277
LTR36	25.1059635	17.4694203	17.7241622	21.8645777	19.2704249	16.2480575
LTR37-int	1574.89172	1597.40973	1533.86102	1433.98588	1479.21995	1353.50253
LTR37A	2956.26494	2991.14332	2874.22268	2736.19121	2645.93146	2612.63732
LTR37B	2187.81999	2173.66295	2105.81397	1915.88223	1907.19173	1863.29472
LTR39	35.5472091	38.3681239	45.063604	38.0418419	45.6510347	44.6228779
LTR40a	4923.28531	4871.7472	4920.78715	4677.32646	4552.95849	4669.68007
LTR40A1	572.856139	628.661583	625.838666	571.32598	566.680546	566.574764
LTR40b	3716.41446	3779.97345	3804.57049	3655.58108	3730.4079	3649.52923
LTR40c	1622.43986	1645.82108	1565.10711	1710.77301	1731.02929	1685.39552
LTR41	3249.26152	3146.00573	3205.60868	3509.43529	3469.47079	3411.69086
LTR41B	1774.02194	1837.17183	1744.36915	1855.71319	1831.74415	1906.61746
LTR44	9.26060265	13.2248359	7.14221185	3.86392068	8.12019588	4.06860106
LTR45	139.198433	129.634681	101.807196	137.033597	144.306375	128.661029
LTR45C	102.365728	81.4704984	86.8188369	96.46622	117.279163	109.012672
LTR48	3401.85962	3389.68324	3349.42239	3507.86412	3551.33819	3478.07898
LTR48B	1917.78566	1974.47774	1987.33355	1940.10264	2022.20779	1967.03697
LTR50	3198.30776	3271.39543	3178.26708	3283.39214	3339.06149	3345.88896
LTR52	1382.29226	1329.24365	1312.18475	1321.03283	1268.71897	1318.31344
LTR52-int	390.889282	362.45427	342.981721	375.243132	348.200961	364.047088
LTR53	1487.71392	1567.04202	1529.3347	1479.18143	1520.34599	1524.51501
LTR53B	575.26356	636.090415	610.16992	636.446077	625.744308	588.181921
LTR55	1027.2983	1041.97401	1084.74563	1029.37981	983.98583	1001.90446
LTR58	41.3581533	38.5311436	35.5140081	41.649749	46.1359865	32.4170747
LTR59	26.9555675	35.3476042	29.1414157	34.1779212	28.0773307	32.4961149
LTR64	32.0975507	19.9185614	30.9762315	21.6550234	26.703911	28.3353003
LTR65	1010.40336	998.872545	951.089096	990.189701	998.647991	957.577981
LTR67B	3617.85175	3597.60376	3432.22179	3728.07349	3779.6523	3795.85652
LTR68	571.05267	520.416081	516.652236	481.431262	468.389835	470.390091
LTR69	522.125191	498.94438	511.607575	472.632908	457.359929	497.731294
LTR72_RN	5908.28859	5913.1467	5819.23419	5916.68073	5732.53701	5745.85247
LTR73	884.957426	927.195981	958.336962	993.188384	964.411547	980.577482
LTR75	569.714748	528.577794	519.486565	598.986439	590.232881	560.471863
LTR75_1	42.4863698	32.6533263	31.0090733	36.4280033	27.0272122	22.331199
LTR75B	427.650163	453.883948	432.764089	391.800627	446.393235	457.058457
LTR77	121.910297	107.267079	106.016554	116.526844	114.087479	109.549241
LTR78	3755.67923	3748.29804	3824.34727	3743.57514	3811.794	3601.15391
LTR78B	2412.11594	2445.99393	2416.84566	2336.85763	2421.26783	2407.35386

LTR79	2246.74781	2270.07485	2287.15	2243.14166	2242.60346	2280.03248
LTR80A	739.548035	741.233786	724.762271	716.735033	578.659082	647.834812
LTR80B	749.268417	694.54012	709.046427	651.12186	534.34136	660.132829
LTR81	388.450405	359.189018	355.635508	418.165369	372.764528	411.652256
LTR81A	1024.79389	1071.76903	1073.56044	1017.28788	1045.20938	1010.63751
LTR81B	1242.98216	1247.20036	1233.33134	1214.64603	1169.88192	1150.3851
LTR81C	618.247981	626.131438	595.967601	593.547558	662.023881	572.147684
LTR82A	2396.57203	2344.60477	2338.54406	2308.59552	2336.97934	2252.82301
LTR82B	1841.522	1866.31365	1819.89713	1862.17991	1814.65296	1828.68526
LTR83	959.639908	974.544154	942.555434	885.789387	891.673139	911.213664
LTR84a	812.151285	811.196113	764.117248	806.431567	829.862537	766.436311
LTR84b	1155.75351	1165.48655	1190.1261	1145.56531	1183.13361	1133.98909
LTR85a	1303.30085	1202.95513	1259.02372	1234.93565	1254.58003	1204.42909
LTR85b	718.043766	716.092825	669.468685	699.626633	668.889759	636.13923
LTR85c	710.867638	684.988895	693.579696	713.158424	691.757825	668.444332
LTR86A1	515.908466	546.702531	555.580269	548.060909	536.966046	542.857807
LTR86A2	378.565928	366.455741	374.112869	357.549226	336.162264	377.71769
LTR86B1	274.330674	255.024879	309.072635	286.846105	262.291692	259.586892
LTR86B2	220.150591	224.982195	228.372949	203.112337	201.289343	224.447454
LTR86C	488.972296	454.211404	538.731381	459.112976	474.752539	470.350571
LTR87	1352.69912	1322.79577	1284.4227	1333.43531	1438.7794	1359.76302
LTR88a	318.023899	333.719488	327.322267	342.306888	370.683734	354.797229
LTR88b	1027.08178	960.911263	1025.65538	1017.75675	974.511047	1027.85332
LTR88c	666.184077	633.968426	680.672466	679.309995	665.942382	593.896208
LTR89	829.314647	848.828423	858.216793	829.758747	791.077991	857.288233
LTR9	60.7716561	51.9192017	69.0946748	51.7789089	60.5581142	60.857762
LTR90A	527.373075	536.66083	537.356351	497.926648	551.611816	479.626777
LTR90B	492.074367	454.700463	469.303323	453.685466	449.725906	415.523257
LTR91	301.135777	296.983383	352.374236	312.69882	274.594139	267.180939
LTR9A1	39.6595373	49.6330803	46.9969453	49.3387117	52.9228701	53.7410035
LTR9D	26.8245015	26.4492672	40.5915111	42.7126819	47.5094062	46.6901118
LTRIS_Mm	5226.84161	5189.62281	5472.27454	5632.9898	5839.95182	6096.05062
LTRIS_Mus	9597.29924	9457.44205	9503.63787	10529.7889	11905.8988	11720.1029
LTRIS2	13292.0265	13209.5008	13364.0382	13863.7875	13262.7755	13769.4037
LTRIS3	1525.17784	1457.24611	1424.5268	1524.67994	1721.95986	1598.19065
LTRIS4	1107.52907	1145.24164	1080.78259	1215.13104	1106.35516	1155.32619
LTRIS4A	1464.33331	1457.81658	1580.4146	1569.67779	1504.44834	1615.6304
LTRIS4B	624.793419	531.27187	574.508124	596.22812	619.13974	672.015884
LTRIS5	659.671143	656.826504	631.908553	700.953648	673.112118	670.166011
LTRIS6	1017.33518	1110.38502	1075.23601	1103.00765	1057.65159	1115.00549
Lx	334383.985	331689.206	338586.664	337812.683	316216.059	334590.034
Lx10	68031.5236	67074.6422	68379.6041	62141.8987	59269.3354	60161.6262

Lx2	215296.71	213200.901	219403.725	224145.967	207577.677	225019.46
Lx2A	17006.7245	17084.6497	17526.2005	18064.51	16690.8851	18209.4388
Lx2A1	26786.7267	26254.5712	27114.6383	28831.939	26531.0952	28655.0795
Lx2B	77770.1529	76521.4233	78609.628	78600.5099	72555.8874	78574.0842
Lx2B2	131534.023	129781.748	133779.725	134639.37	122680.078	133040.819
Lx3_Mus	177781.049	175688.765	180648.123	179532.458	165526.04	178701.716
Lx3A	106922.057	105232.745	109015.869	109993.282	99207.0364	109030.942
Lx3B	66877.53	66094.3234	68101.6706	69992.8207	63555.7139	69272.9584
Lx3C	79366.0383	78324.696	80813.1798	82146.2069	74190.8155	80694.2338
Lx4A	126101.353	124398.178	127918.197	128881.728	119345.164	127430.808
Lx4B	64278.7073	63764.3998	65315.2194	66663.5754	61663.1298	65270.4868
Lx5	208471.388	206801.056	210310.306	212050.15	198976.596	210546.343
Lx5b	87819.943	86578.6791	88668.2769	85687.8143	80228.1887	84399.4897
Lx5c	166472.299	164816.013	168669.089	162749.426	164181.239	164507.173
Lx6	188567.041	186111.244	190213.024	174307.744	163230.664	169819.349
Lx7	343211.075	341048.084	345688.088	314906.547	301462.019	306302.484
Lx8	401223.677	399670.098	401358.032	393203.728	385711.556	389855.06
Lx8b	192527.798	190540.583	192986.278	179249.692	171301.239	175049.461
Lx9	362418.004	359213.288	364242.264	334051.467	320677.027	325863.857
MADE2	746.703717	676.011678	697.709114	656.327957	677.051283	641.797777
Mam_R4	837.51309	851.930351	842.768647	770.077043	767.384059	756.192355
MamGyp-int	277.623053	294.698881	281.259151	264.259946	304.610056	349.550103
MamGypLTR1a	832.804149	829.31741	832.219539	811.944415	817.277507	768.671259
MamGypLTR1b	794.548591	818.134672	770.764668	764.681319	790.553542	807.510444
MamGypLTR1c	775.961852	806.625591	723.359364	767.858259	818.932899	789.623287
MamGypLTR1d	678.035593	643.274717	676.313156	648.053488	650.750888	671.002027
MamGypLTR2b	544.837888	541.070862	532.443057	522.786119	565.105369	521.109284
MamGypLTR2c	540.811015	534.048163	599.221745	577.687928	532.846397	533.420474
MamGypLTR3	613.237064	581.232608	585.230734	600.08825	542.462166	590.80853
MamGypLTR3a	966.276569	987.116708	933.207853	1024.82133	977.299215	956.850398
MamRep1151	1542.10424	1642.63501	1565.51331	1422.4676	1395.09006	1395.84081
MamRep1161	41.4892194	37.9595627	31.7786928	38.6780855	40.0358128	46.6505917
MamRep137	1820.63322	1840.92543	1917.41999	1829.09243	1961.1635	1807.03859
MamRep1527	1848.28344	1806.55776	1896.79366	1792.03956	1806.25079	1845.01187
MamRep1879	491.221914	516.089178	500.033241	510.767668	526.563908	502.26401
MamRep1894	137.545952	145.960636	143.970788	162.525521	154.385822	161.302051
MamRep38	1095.32315	1103.36242	1125.37928	1188.47568	1259.56993	1192.74363
MamRep4096	993.330698	1059.44262	998.902759	950.413594	994.671771	979.280398
MamRep434	2900.16239	2841.42868	3015.7204	2821.36863	2869.62275	2707.50161
MamRep488	142.150041	160.981522	158.320896	141.661768	174.483943	178.228044
MamRep564	693.251313	680.009811	715.283323	689.090711	635.702822	642.785287
MamRep605	3871.47766	3803.15494	3901.11243	3702.01452	3632.827	3667.69962

MamSINE1	2091.49379	2172.44041	2028.12924	2199.04792	2200.12345	2209.39842
MamTip1	384.101633	398.536677	405.661669	394.535716	424.919863	415.753791
MamTip2	3720.57083	3601.35898	3675.93227	3733.92768	3738.61136	3672.03168
MamTip3	1020.45036	988.25987	1032.11504	1003.33698	1029.97656	967.611163
MARINER1_EC	5.19440956	6.85714996	8.84566015	8.91499068	3.55509241	7.10358513
MARNA	3224.4979	3323.80217	3243.54713	3100.53005	3188.39302	3106.66838
MER101B	102.365728	104.001625	102.004248	109.477915	88.9587461	81.121727
MER102a	2251.62819	2287.46246	2265.63197	2308.04792	2517.01956	2365.76242
MER102b	3207.87715	3142.73805	3067.8627	3318.00384	3828.19116	3644.4569
MER102c	1983.37006	1974.47805	1962.53503	2082.12189	2191.61796	2124.13868
MER103C	5394.27534	5481.06049	5400.33688	5280.22231	5337.86098	5331.70534
MER104	1356.13672	1404.59039	1282.47293	1265.05954	1269.3449	1177.75923
MER105	794.12184	812.909541	808.573278	819.346554	836.022872	856.192289
MER106A	622.760323	651.436936	634.510824	629.882155	663.92236	665.580601
MER106B	801.297444	800.909174	875.516175	796.729097	788.353036	767.190241
MER110	844.439668	839.03206	868.87372	766.989719	775.425261	772.193658
MER110-int	273.471405	254.126853	289.349448	256.594213	280.855963	252.907901
MER110A	1172.33389	1157.81177	1096.2707	1012.67487	1002.81202	1085.0539
MER112	1689.0298	1680.84082	1687.72624	1577.86692	1664.85596	1507.68428
MER113	3780.55085	3880.05613	3832.59961	3747.55472	3950.06281	3822.4843
MER113A	698.866182	718.540246	712.303371	670.817904	712.483106	676.765961
MER113B	317.709341	389.229273	351.02492	296.35088	329.415488	340.682272
MER115	3191.94581	3338.00866	3337.40189	3457.03958	3535.48309	3548.28186
MER117	3743.06753	3876.30161	3859.50498	3804.67629	3878.21165	3937.43825
MER119	2545.7404	2558.07819	2438.42721	2433.26932	2639.45027	2457.85569
MER121	3742.01166	3784.38278	3727.71177	3451.97616	3551.88513	3332.6926
MER123	210.562323	209.635674	230.789625	182.946936	200.966653	180.677306
MER124	1420.16144	1347.28658	1364.25424	1339.44783	1287.18236	1290.85722
MER125	490.054902	476.579916	480.007348	447.121544	405.246532	445.234682
MER126	701.082247	688.418886	652.678352	608.188253	635.603164	630.619004
MER127	723.473046	737.479475	663.734345	679.375894	681.496064	680.330927
MER129	378.874195	373.066945	381.935467	369.020562	348.40394	387.451424
MER130	574.338234	566.375551	557.440799	584.60127	597.26163	610.654487
MER131	1250.40679	1260.2639	1209.01182	1206.61193	1220.70516	1144.06533
MER132	87.7010105	79.1845794	112.389147	104.357157	76.8799418	77.0399526
MER133A	134.010839	120.001237	129.74492	134.632278	139.417971	139.767348
MER133B	84.3562049	88.1646286	89.1276954	64.1505704	60.9822944	89.1403687
MER134	246.679932	231.840155	243.304917	223.553192	240.617172	214.977187
MER135	1118.51293	1139.36382	1163.94605	1170.48261	1024.88761	1137.70554
MER136	140.352863	126.205701	151.784094	129.666546	106.330692	116.442052
MER2	49971.0516	50325.3699	49909.0761	48596.5396	50769.2957	48453.6353
MER20	31747.0843	32012.7074	31866.1691	33156.1082	34746.8237	34006.3369

MER20B	5095.9124	5018.35719	4991.79938	4939.04522	5009.10971	4867.94911
MER21-int	640.63878	659.764198	626.401942	623.197808	719.088285	621.467945
MER21B	11996.2032	12219.3609	12096.5995	12633.4785	12985.863	13026.399
MER21C	17661.2285	17863.1957	17659.2609	18056.4184	18950.4358	18246.2148
MER2B	4261.38237	4267.0821	4232.62547	4195.32767	4325.37181	4117.95747
MER3	14230.8662	14321.9196	14211.1476	14245.8168	14577.6943	14439.7115
MER31-int	984.432361	977.810013	934.158103	951.193005	901.610378	841.030542
MER31A	4854.04049	4755.16968	4817.91196	4988.78361	4819.91617	4855.69671
MER31B	3531.71305	3518.33605	3574.15422	3632.69294	3540.45354	3543.20599
MER33	13353.8986	13502.4779	13205.3239	12559.3613	13430.4046	12851.3058
MER34	3813.17948	3861.85221	3821.32735	3702.26295	3572.32173	3654.98713
MER34-int	500.967461	490.783376	524.531226	479.755804	519.475609	514.216472
MER34A	4089.08452	4184.95336	4129.71743	3966.30246	3917.29028	3965.23344
MER34A1	3132.04601	3270.66022	3268.19723	3061.55708	2985.26353	3012.7217
MER34B-int	482.722019	457.720679	479.467622	422.82056	445.402667	424.759943
MER44A	3829.09719	3945.7681	3868.02006	3514.27155	3826.47927	3686.26215
MER44B	9367.99867	9295.89026	9217.70398	9089.55379	9255.56333	8953.37351
MER44C	1326.65	1385.9775	1276.94277	1246.74076	1271.44819	1222.1617
MER44D	4712.72875	4780.6383	4575.19883	4491.58135	4515.99343	4406.43295
MER45A	3500.59063	3583.31424	3588.56938	3755.72539	3850.59922	3756.28415
MER45B	4504.61998	4432.55385	4436.10569	4376.86274	4529.24451	4289.42798
MER45C	1733.43026	1635.37041	1779.7775	1876.85239	1841.31798	1901.08662
MER45R	1845.49016	1805.57651	1839.66525	1833.2702	1812.77697	1780.02672
MER46C	4020.78338	4118.34487	4017.59815	3734.86494	3884.73661	3817.06287
MER47A	675.051482	723.928903	678.065867	700.883471	730.499212	700.979967
MER47B	2889.76046	3033.59352	2942.14434	2952.622	3001.00074	2958.92495
MER47C	48.8808202	43.265799	33.5314039	50.2233883	41.2074744	41.5747204
MER49	26.6672221	30.3680144	15.1197269	28.3199319	27.3906208	32.4302481
MER4B-int	248.286801	260.575442	255.327581	273.260275	239.001887	253.688177
MER4CL34	19.6232086	15.0206839	19.4932942	25.006917	19.7954842	12.1926298
MER50	41.3581533	49.3066361	43.3929975	45.0481018	33.0872786	29.4084374
MER50-int	89.734107	90.6131627	76.0398354	86.9149858	116.027287	85.1376345
MER50B	59.0793314	58.9395737	57.5296328	61.4349203	59.3062377	57.8359513
MER51-int	48.4021669	49.7967071	32.8110472	34.5192726	53.8927738	42.5292971
MER52-int	1418.59389	1369.65246	1431.467	1406.60943	1442.13152	1403.88226
MER53	7084.18433	7029.97778	7069.40296	6634.04345	6788.79757	6559.44399
MER54A	2083.99157	2100.52134	2142.26894	2109.69604	2078.27918	2194.05529
MER54B	914.425792	939.685811	869.920331	868.13057	903.509467	946.81063
MER57-int	998.381459	1050.95386	1023.80198	952.427102	981.498467	943.064776
MER57C2	2597.28972	2573.26017	2535.45248	2383.46883	2417.42832	2425.04391
MER57D	1849.58833	1900.76009	1885.25433	1646.94814	1636.86128	1624.1167
MER57E1	1216.57759	1203.77387	1191.61391	1150.12709	1157.45915	1165.31985

MER57E2	374.755576	388.250648	382.40951	350.674763	337.333926	351.278369
MER57E3	19.7280614	31.8368111	15.8893464	13.5004928	15.9170905	13.1867266
MER58A	19582.5314	20009.5947	19864.7867	19442.0275	20017.7445	19037.3393
MER58B	15060.6097	15176.4628	14839.3844	14773.9759	15145.7574	14899.6185
MER58C	1818.92674	1851.70133	1827.03934	1690.30892	1790.23465	1766.54054
MER58D	1251.20052	1284.58935	1203.94144	1255.34948	1266.92137	1201.38752
MER5A	38428.0271	38643.99	38918.0666	39847.9721	40270.5273	39945.7702
MER5A1	17097.0269	17298.3694	17066.0889	17657.3911	17882.6184	17845.1318
MER5B	21707.9409	21767.7446	21605.5525	21873.1947	22520.9003	22336.108
MER5C	1187.73363	1285.16093	1230.19214	1128.73994	1188.60906	1123.45885
MER5C1	386.567248	391.842243	364.816715	330.582841	405.648828	376.98352
MER63A	3338.56258	3470.90637	3405.97606	3373.72492	3479.22511	3450.42466
MER63B	3526.57578	3548.29885	3633.44306	3613.80333	3685.84892	3544.41086
MER63C	3077.78991	3154.08472	3166.72775	2776.78182	2758.22256	2687.9029
MER63D	5114.8268	5253.87287	5054.80711	5003.0673	5022.78252	4893.53167
MER65-int	238.954374	255.677868	227.57978	236.46769	234.194918	283.310436
MER65D	563.313482	571.028292	607.957423	562.310003	528.139697	562.539097
MER66-int	83.2542017	89.7976592	102.248397	95.531294	92.3734626	83.2021343
MER66A	17.7473913	18.9390264	14.0873725	17.7911027	24.5222383	17.2158076
MER66B	28.1099972	34.7762256	34.8100723	32.0131769	31.3905577	36.4988491
MER66C	81.7065737	77.0622872	74.0408103	78.2019689	74.2534246	74.9990653
MER67A	1167.80792	1148.26226	1135.27588	1155.83433	1221.23083	1245.38161
MER67B	1431.73719	1424.6743	1406.89187	1472.40844	1474.33216	1456.66869
MER67C	4012.91942	3912.95247	4064.41663	3619.45176	3406.20534	3573.70782
MER67D	2783.26353	2819.79558	2862.14146	2603.16189	2218.5048	2466.69413
MER68	4929.66613	4922.76666	4993.47152	5072.28508	5225.85063	5051.73641
MER68-int	506.476953	540.824815	520.828753	481.446911	537.835681	483.165396
MER68B	1508.10518	1552.92038	1524.22715	1533.85473	1602.43581	1689.19102
MER68C	272.664562	230.696588	277.523836	282.253023	262.656321	268.691844
MER70-int	459.780742	422.61892	458.139512	444.018084	421.768897	416.649087
MER70A	1357.83534	1372.26401	1337.36312	1290.26464	1354.91143	1233.41952
MER70B	1730.23067	1715.37302	1719.11083	1753.04005	1623.76941	1685.54701
MER72	41.7649822	32.0002355	26.5369804	37.3202605	35.6323599	36.5515426
MER72B	224.10564	236.493402	221.312841	200.059126	211.772916	222.531714
MER73	952.595371	927.196183	898.808304	934.014917	880.94709	873.773411
MER74A	5227.23953	5279.83237	5175.33085	5611.64055	5608.66967	5524.81544
MER74B	2296.78411	2366.56462	2270.66237	2402.12945	2602.38006	2438.4475
MER74C	759.007673	718.378643	723.509316	731.127783	681.030556	641.280969
MER76	1555.55633	1595.86105	1636.33931	1662.6333	1524.28332	1716.6903
MER76-int	123.405499	98.4504548	119.465675	123.125854	76.9200492	90.2266792
MER77	4209.10275	4227.97806	4279.22335	4313.47263	4287.67688	4245.80579
MER77B	3703.72937	3609.36041	3796.24814	3731.64154	3755.47708	3665.06642

MER81	2063.04249	2130.40019	2159.73966	2237.17131	2322.45084	2313.45682
MER83A-int	7.25371933	4.4082112	0.90098695	1.5285007	1.01001106	2.01454047
MER83B-int	42.5650094	35.9187804	50.3381582	30.5311354	37.8541401	40.5279301
MER87	230.362733	195.676035	194.571681	210.312503	217.87309	253.46423
MER88	370.58453	351.515353	399.53539	331.855328	371.916168	400.414203
MER89	4217.69753	4171.4045	4279.4111	4198.63078	3965.87719	4030.0615
MER89-int	685.885926	735.602117	705.700885	674.06881	607.30219	619.002463
MER90	1566.52079	1587.04462	1566.46789	1469.11866	1469.70567	1513.71473
MER90a	3565.96794	3394.33284	3521.03532	3437.95227	3451.14961	3529.12347
MER91A	1857.81351	1816.67915	1804.7424	2021.82008	2122.41581	2067.89926
MER91B	1008.73777	991.932267	1020.70491	1049.10667	1116.49476	1071.49222
MER91C	489.326175	494.864131	438.695481	517.362888	518.444323	499.640448
MER92-int	1943.78288	1942.3965	1930.90692	1791.39145	1871.76113	1748.71876
MER92A	209.38168	206.370119	214.736069	193.817117	205.491038	191.480637
MER92B	1576.05873	1572.43149	1585.78768	1475.86084	1418.01524	1508.8628
MER92C	377.038222	375.351447	380.706062	380.647413	365.936316	371.17702
MER92D	380.39561	380.7398	410.485729	363.348898	345.65588	339.622308
MER94	1993.41759	2096.27442	2108.7332	1994.05387	2078.31929	2079.2954
MER94B	393.040862	351.842505	360.975746	321.489594	350.485345	343.559175
MER95	290.832413	299.351419	291.301375	281.092893	252.576875	256.446521
MER96	538.902693	457.63836	497.520203	505.204571	511.131159	488.547302
MER96B	2000.72373	1866.96694	1951.06202	1886.89194	1867.0131	1926.76946
MER97a	405.567631	435.353078	397.055194	360.384815	397.56996	381.262895
MER97b	327.015555	330.943093	328.875763	292.579878	309.115609	300.167515
MER97c	497.347941	472.169074	476.689685	425.94725	412.517147	365.080705
MER97d	137.237685	132.900236	153.046342	140.156497	112.71406	115.579689
MER99	1189.33368	1181.56615	1112.7512	1078.49711	1116.41455	1115.89774
MERV1_I-int	20823.0522	20550.9899	20744.1318	22275.3986	21329.0923	22852.103
MERV1_LTR	4790.90545	4757.70165	4923.25092	4959.64869	4735.198	4838.03606
MERVK26-int	13651.6151	13484.1978	13799.3318	14229.696	13365.5481	14313.595
MERVL-int	97071.7076	96655.3537	97208.162	99513.7123	98170.0548	100575.736
MERVL_2A-int	45250.1779	45051.3398	45746.1371	44379.383	44312.8549	44193.747
MERX	348.193206	359.842311	347.552339	290.15912	301.499808	292.998063
MIR	188145.816	189679.146	186801.675	191562.713	198439.906	194899.833
MIR3	69931.7689	70346.8206	69333.4896	74762.6782	78293.0529	76601.2938
MIRb	208521.816	211307.15	208919.202	215899.092	223368.56	219140.432
MIRc	102934.516	103314.635	102923.036	108613.068	111937.906	110258.16
MLT-int	6205.35608	6150.12552	6085.92913	6081.5995	5919.74758	6009.05751
MLT1-int	1878.56546	1781.49477	1864.36029	1715.69607	1667.56025	1603.01976
MLT1A	29606.1372	29484.5129	29541.2539	29627.5814	30352.6935	30116.4668
MLT1A-int	850.847225	866.133811	855.661621	732.80373	690.948351	689.429292
MLT1A0	64358.5187	64223.0463	64268.7796	63943.1156	63508.3282	63709.9471

MLT1A0-int	1741.04415	1774.88407	1720.18316	1656.80184	1628.0726	1676.54086
MLT1A1	46011.3618	46070.6191	46125.5945	45528.4549	46737.0585	45821.569
MLT1A1-int	1162.64077	1164.58842	1173.67844	1168.41885	1083.26894	1116.92477
MLT1B	57451.2861	57538.4622	57597.4209	58413.3546	58918.9102	57915.5648
MLT1B-int	1145.29339	1101.48314	1090.49639	1049.56796	997.982556	1067.53206
MLT1C	50839.3322	51015.1	50206.5521	51822.9939	52137.204	51902.2147
MLT1C-int	825.136785	864.992167	844.070865	799.390219	802.06779	756.972631
MLT1D	54533.4031	54832.212	54777.2249	55206.4697	55674.633	55211.6909
MLT1D-int	1397.77011	1448.51069	1316.87961	1374.84799	1327.37921	1276.61053
MLT1E	3294.6801	3473.02897	3318.77674	3376.28408	3294.76138	3350.33959
MLT1E-int	55.7869523	58.4497051	57.5132119	60.4184467	56.1145539	49.6724024
MLT1E1	3677.10933	3653.35899	3840.87983	3707.75587	3816.13608	3705.27514
MLT1E1-int	82.4279612	64.3275223	81.2487059	74.4698452	54.417833	59.824145
MLT1E1A	7677.83384	7798.88489	7614.29996	7635.64812	7450.5352	7591.2732
MLT1E1A-int	215.356195	210.941451	202.811931	202.134742	189.190485	185.463363
MLT1E2	12004.0257	12205.2366	12284.8891	12000.5725	11875.5079	11925.2258
MLT1E2-int	275.944359	232.167105	269.546321	261.303932	196.62275	253.372016
MLT1E3	6902.31709	6768.50937	6643.06786	6610.90357	6619.33569	6608.25658
MLT1E3-int	85.6417007	79.0213573	97.4007872	91.5004879	69.3650199	67.8954802
MLT1F	11320.651	11607.5127	11262.0603	11839.0422	12213.2411	12230.4225
MLT1F-int	2943.64066	2851.55118	2875.09579	2728.44442	2704.32552	2626.14581
MLT1F1	10527.0524	10657.0482	10601.1352	10844.9762	11133.8579	11031.6885
MLT1F1-int	628.086323	645.886777	650.533702	605.755637	636.208438	634.634912
MLT1F2	16472.9344	16530.6901	16415.3898	16721.5874	16702.513	17055.8545
MLT1F2-int	599.936482	527.353831	596.762934	514.740156	524.059545	523.933494
MLT1G	6081.44467	6083.84419	6181.68999	6188.80508	6256.91924	6080.27425
MLT1G-int	277.308494	287.188033	252.920197	282.601954	271.280912	268.665497
MLT1G1	7129.84145	7169.73597	7338.71163	7430.9371	7412.48114	7446.98277
MLT1G1-int	456.756786	480.496437	461.283674	466.239174	443.704726	482.674934
MLT1G3	7484.60683	7433.01217	7376.09113	7790.80182	8052.96782	8202.45108
MLT1G3-int	547.272046	557.39449	540.075733	488.670306	533.796247	507.511135
MLT1H	15138.2931	15120.7045	15113.0173	15252.0615	15257.0902	15542.6799
MLT1H-int	2573.88971	2555.29967	2541.52301	2439.30508	2337.54206	2372.43837
MLT1H1	6553.18125	6542.70884	6509.89529	6476.19402	6648.84843	6580.89562
MLT1H1-int	397.985722	408.495654	408.570973	348.963728	379.389761	393.310618
MLT1H2	6018.4905	6142.6177	6086.69658	6039.15567	6054.71894	6086.55804
MLT1H2-int	474.550313	432.005911	483.644138	457.611495	464.934401	466.732911
MLT1I	10570.494	10619.828	10457.6778	10542.271	10589.4583	10622.4455
MLT1I-int	461.223517	471.518512	466.985172	434.85466	372.178087	406.589558
MLT1J	18184.7215	18425.077	18024.838	18711.1113	19520.0443	18644.6702
MLT1J-int	3542.86048	3544.6218	3547.10259	3383.86117	3370.0072	3355.10235
MLT1J1	6251.72305	6203.35606	6084.87602	6323.6578	6369.89095	6394.85617

MLT1J1-int	539.617789	478.782099	517.684591	450.217424	510.847965	477.467329
MLT1J2	6649.94836	6582.87271	6568.57159	6721.58428	6713.37126	6564.84821
MLT1J2-int	235.20274	246.207748	216.800778	227.545119	246.556916	211.863163
MLT1K	17287.2934	17375.0289	17245.1006	17954.6764	18441.5033	18154.6621
MLT1K-int	64.3261674	69.8784907	66.5887651	66.9980174	50.499332	70.0022343
MLT1L	9422.77694	9328.22129	9346.26659	9447.42688	9465.33383	9480.09828
MLT1L-int	88.186479	82.9400033	73.2711908	59.0141625	49.9742728	72.9976982
MLT1M	1409.54351	1430.63393	1437.89598	1520.59509	1561.41065	1547.02356
MLT1N2	3746.69439	3759.40403	3700.30385	3792.98782	3975.06512	3860.21741
MLT1N2-int	20.9349176	30.2045899	26.6026642	24.5413491	33.2489292	22.3443724
MLT1O	2998.48132	3117.92121	3115.91618	3348.64782	3424.78966	3382.64469
MLT1O-int	20.3708093	15.3471281	16.888859	15.4556827	15.3920313	13.1735533
MLT2B1	11187.3128	11198.93	11273.9029	10950.5101	10843.9933	10972.3117
MLT2B2	8670.12439	8835.80458	8784.13527	8304.4843	8338.23518	8290.56747
MLT2B3	10283.2586	10239.3304	10288.7821	10448.3265	10262.3436	10387.5894
MLT2B4	11953.2476	11921.0675	12173.361	11418.1365	11341.8533	11387.6475
MLT2B5	2302.22125	2300.19611	2252.73403	2486.95696	2333.32092	2221.12644
MLT2C1	5304.61778	5238.68411	5200.4578	5148.09661	5040.69836	4976.29653
MLT2C2	3851.34958	3959.32222	3865.26007	3694.43364	3706.81546	3711.08164
MLT2D	9566.79441	9674.50494	9619.02212	9350.42394	9377.08124	9244.84799
MLT2E	802.136791	749.561554	734.081974	711.016909	738.055462	774.652554
MLT2F	3765.04364	3705.76786	3801.91183	3774.47513	3833.34637	3883.96376
MLTR11A	18587.8959	18642.6321	18668.9461	19682.0231	19745.6498	20091.1243
MLTR11B	23823.6898	23906.7957	23719.7118	25425.431	24858.3646	25342.2212
MLTR12	3226.0125	3125.18763	3167.67303	3171.98459	2994.90118	3068.92209
MLTR13	1601.96472	1583.3703	1564.42889	1507.64172	1601.12377	1518.51091
MLTR14	47105.0786	47110.2388	47193.4299	49266.2214	49640.8338	49591.5157
MLTR18_MM	3791.91637	3930.01575	3774.11973	3855.50083	3845.74726	3716.78325
MLTR18A_MM	2817.55146	2752.28391	2879.64999	2742.55744	2662.73763	2755.45233
MLTR18B_MM	3989.13774	3964.87389	4020.85013	3989.11517	3936.05815	3890.97209
MLTR18C_MM	4943.0595	4876.39499	4849.8877	4889.63255	4842.94466	4758.38925
MLTR18D_MM	6621.48763	6648.42156	6552.7814	6812.86103	6676.83282	6647.37546
MLTR25A	19244.51	19262.4811	19393.6185	19116.3128	18216.9739	18842.2436
MLTR25C	6272.9741	6086.86187	6440.97628	5878.08749	5316.8634	5579.02714
MLTR31A_MM	3273.50612	3183.14676	3290.36561	3339.27484	3352.41405	3382.71665
MLTR31C_MM	2134.04674	2008.43766	2161.15682	2206.83029	2001.70188	2047.10227
MLTR31D_MM	2973.5059	2930.89635	3057.02018	3064.70699	2951.55214	3021.42536
MLTR31E_MM	1321.97357	1279.69158	1343.63936	1279.23185	1411.45016	1286.98622
MLTR31F_MM	3662.07553	3656.21507	3555.5777	3630.48174	4643.77195	4305.12885
MLTR31FA_MM	5228.61048	5131.74692	5287.94277	5229.37502	4894.12399	5205.7302
MLTR32C_MM	913.723278	949.564999	943.156515	891.135349	819.155321	864.151651
MLTR73	5236.86869	5294.44085	5252.85137	5306.36942	5481.91877	5525.51363

MMAR1	2575.61402	2597.42584	2653.21687	2395.14972	2471.12666	2575.12108
MMERGLN-int	20170.3679	20221.2705	20236.3295	22870.0428	25133.8289	24986.1927
MMERGLN_LTR	3657.81326	3646.50163	3693.3872	3688.97959	3625.2902	3732.03976
MMERVK10C-int	54322.1907	53565.6738	54369.6042	53139.0126	50360.503	51882.8065
MMERVK10D3_I-int	10120.3555	10175.7412	10435.4508	10730.4984	10401.1733	10767.1073
MMERVK10D3_LTR	1321.69833	1305.08081	1372.35599	1355.38094	1348.78998	1414.5574
MMERVK9C_I-int	25141.7896	25374.1511	25255.3482	22815.9842	21637.4331	22117.5532
MMERVK9E_I-int	27984.8984	27734.6022	28223.6784	26039.925	25309.6432	25760.9628
MMETn-int	23941.4705	23750.6184	23620.8873	21412.7674	21555.6666	20751.1862
MMSAT4	11558.3121	11834.7099	11780.0897	11180.6223	10675.6411	10833.7695
MMTV-int	675.727258	724.989948	662.678441	669.786269	624.895948	639.63833
MMVL30-int	12418.4382	12287.1143	12674.1118	12769.5544	12578.8566	13145.3983
MRLTR33	3569.31065	3593.27413	3611.48599	3672.28651	3643.16959	3469.10881
MT-int	3143.1473	3158.9824	3131.60071	3223.44586	3247.02955	3191.08806
MT2_Mm	18166.3916	17832.2643	18143.6304	17887.182	17226.7293	17615.1996
MT2A	64356.1511	64535.3733	64361.1748	65183.2759	64103.5157	64507.706
MT2B	39637.6996	39797.7979	38903.4628	41590.298	43459.5134	43646.1423
MT2B1	44299.4578	43818.4116	43972.1423	42768.9288	42384.7257	42216.5751
MT2B2	16731.2263	17074.6077	16672.6479	17617.4704	17833.2895	18104.667
MT2C_Mm	16438.4184	16422.8535	16597.4861	16927.5898	16564.0315	17009.0489
MTA_Mm	124371.208	124606.453	125384.198	134473.682	136191.536	138739.955
MTA_Mm-int	57279.3159	57548.6046	58357.7467	59159.0001	63563.7316	62811.5579
MTB	53918.6373	54439.4071	53841.3314	58213.2707	57707.7102	58732.85
MTB-int	8999.19297	8993.76923	9133.27611	9117.26645	9035.43741	9274.97034
MTB_Mm	39805.8044	40028.4193	39550.9004	42586.5074	42390.5499	43246.9334
MTB_Mm-int	5436.08436	5595.17916	5519.5612	5455.52392	5494.95048	5316.04045
MTC	187096.292	188588.292	186717.917	199072.522	204733.982	204852.515
MTC-int	34410.2079	34454.6245	33954.0055	34576.9752	33592.5703	34649.2229
MTD	350787.533	351881.91	351635.385	364417.305	370245.905	369609.394
MTD-int	25106.2838	25012.6071	25155.7863	24893.5467	24488.8094	24735.2096
MTE-int	42436.7771	42255.0503	42396.3822	41516.6908	40441.2155	40804.1234
MTE2a	105831.199	105563.066	105795.116	105760.043	106611.772	106511.144
MTE2a-int	4264.07289	4309.69522	4471.88244	4275.84941	4054.63174	4263.87078
MTE2b	93475.9064	93973.5083	93715.4297	95834.1147	95143.0639	95971.2067
MTE2b-int	7387.75689	7439.9485	7308.39503	7026.1205	6821.42117	6889.29305
MTEa	128148.286	128667.901	128386.741	132360.499	133172.732	133549.179
MTEa-int	15017.5382	14856.3743	14814.8229	14606.5828	14422.2432	14453.412
MTEb	65057.68	65590.4131	65219.5958	67413.4462	67544.2747	67322.0026
MTEb-int	6799.69761	6853.31874	6948.55793	6805.06962	6814.79899	6672.90326
MuLV-int	4222.29323	4358.6748	4269.54455	4540.10752	4294.8229	4475.98729
MurERV4-int	14237.0667	14132.7715	13755.1041	12749.25	12785.6287	12646.4008
MurERV4_19-int	11765.0258	11746.4507	11847.7679	10419.4058	10011.8178	9946.85548

MuRRS-int	9661.63694	9817.28628	9943.23091	10634.8268	10390.0425	10646.8307
MuRRS4-int	13177.5964	13226.8043	13394.3347	14369.4704	14042.4627	14364.1136
MurSAT1	907.912334	964.505083	1051.24924	1053.77751	966.369576	1065.90259
MurSatRep1	13843.3574	13887.219	13560.0206	13283.5108	12697.3846	12650.9269
MURVY-int	5625.2111	5468.49178	5837.93801	6702.96022	5582.5297	6954.4991
MURVY-LTR	6127.56314	6184.33745	6328.68006	7103.56776	6905.08494	7190.01944
MusHAL1	42460.7952	42158.2903	42196.1528	36093.3596	34467.1984	34807.0406
MYSERV-int	13637.4212	13552.4375	13628.5762	12257.7887	11917.5559	11917.4113
MYSERV16_I-int	27892.921	27849.3709	27679.3955	24695.1168	23783.8966	23477.6105
MYSERV6-int	35803.1469	35689.6438	35464.8499	31402.3995	30696.519	30544.1928
nhAT5a_ML	17.7211781	11.4287856	10.5491084	13.4151549	12.6852994	22.331199
ORR1A0	15781.16	15877.9514	15956.5247	16869.0854	16547.6811	16990.114
ORR1A0-int	10705.9681	10718.5236	10724	11051.5457	10734.0102	10923.4064
ORR1A1	28344.8293	28234.0428	28536.8876	30365.9264	29982.4004	30695.8134
ORR1A1-int	17715.7556	17810.14	17981.6885	18160.8634	17350.8027	18099.6722
ORR1A2	105435.237	105494.584	105105.399	112460.439	112949.498	113681.691
ORR1A2-int	36542.7107	36557.103	36757.5871	37006.5134	36201.4407	36835.9401
ORR1A3	30470.751	30710.3222	30259.6253	32109.3228	31929.8443	31772.5162
ORR1A3-int	15966.7023	15909.626	15968.1235	15447.2307	15118.2337	15472.2926
ORR1A4	55877.7842	55821.7048	55362.4801	58579.5328	59369.8485	59747.4949
ORR1A4-int	22425.7131	22426.7731	22438.2219	22247.4863	22016.8332	22280.2207
ORR1B1	112718.902	113074.36	112636.714	116904.42	117675.99	118033.283
ORR1B1-int	29716.7129	29689.2527	30068.2777	29160.8301	27977.9214	28784.4483
ORR1B2	56388.6566	56295.015	56668.4268	58801.8603	59259.7944	59401.9632
ORR1B2-int	10207.6046	10193.702	10191.5005	9850.99752	9799.24755	9604.69779
ORR1C1	51347.7657	50850.2926	51461.5458	52423.4681	52449.6128	52708.082
ORR1C1-int	9726.20899	9918.26719	9806.18453	9386.22804	9558.06521	9381.19881
ORR1C2	65210.7306	65196.1259	64348.6466	67350.5093	68172.7957	68241.3464
ORR1C2-int	10912.677	10906.0291	10834.6135	10415.1252	10280.4412	10380.5648
ORR1D-int	3912.36345	3916.95282	4013.2503	3851.42259	3614.54165	3764.03628
ORR1D1	127115.785	127151.628	127168.097	131094.208	133132.254	133062.156
ORR1D1-int	17565.5199	17887.1921	17675.7272	17533.5475	17493.78	17374.6049
ORR1D2	97059.2542	96859.6042	96604.3011	99152.1265	99349.5522	98708.2359
ORR1D2-int	12145.0345	12088.2542	11951.1614	11653.9532	11526.54	11518.4979
ORR1E	154592.552	155675.063	154884.47	160227.011	160129.755	160002.495
ORR1E-int	15785.4128	15940.4789	15879.6117	15422.6363	15117.9025	15081.6851
ORR1F	148007.909	147958.566	147841.637	151063.486	152781.479	151393.11
ORR1F-int	3748.30231	3721.44183	3650.03417	3511.8124	3546.41334	3496.32486
ORR1G	28474.8243	28630.7041	28576.3534	29021.5887	29066.4755	29159.3114
ORR1G-int	604.783828	608.662018	682.204577	608.161234	617.037671	572.822573
ORSL	1258.20522	1208.18137	1237.36007	1174.33895	1206.36447	1217.96491
ORSL-2a	463.617307	416.658884	403.49347	407.954661	419.022058	444.852654

ORSL-2b	1367.17563	1274.1396	1317.41934	1246.90764	1253.20661	1251.74798
PB1	44143.5941	44191.4051	43355.0281	46973.5239	51569.6417	49635.0777
PB1D10	234015.847	235614.15	231212.53	246022.33	264673.889	256645.135
PB1D11	60969.1083	61218.8279	60548.586	63989.6629	69530.1987	67859.8398
PB1D7	89411.5367	89966.3523	88164.7739	95441.6839	102901.791	100611.893
PB1D9	99630.8246	100103.306	97611.9981	106019.886	115716.602	112076.925
Plat_L3	3268.85904	3234.00542	3066.66893	3169.2462	3132.90013	3130.12187
polypurine	2995.7672	2925.75607	2985.67087	3099.50649	3277.53469	3176.04488
polypyrimidine	2912.66504	2765.09775	2883.7915	2914.89925	2925.51305	3025.11193
RCHARR1	50039.368	50202.432	49633.2998	46613.8005	49142.1213	47166.8188
Ricksha	992.701581	1015.19779	1019.46125	998.79794	1051.75623	999.047314
Ricksha_0	682.647021	652.416471	676.822204	591.176562	578.517485	586.578802
Ricksha_a	256.865336	286.698063	258.865845	254.196685	267.645605	229.158011
Ricksha_b	47.0642447	50.6130201	57.4146863	50.3087262	44.4392656	43.5102206
Ricksha_c	2105.65364	2053.74565	2127.66755	2021.25634	2172.24849	2114.675
RLTR1	318.135568	362.127825	386.196252	358.903261	336.1416	339.661828
RLTR10	15116.7458	14866.8997	15426.9602	17086.171	17631.368	18006.3655
RLTR10-int	18639.388	18653.4902	19013.0646	21298.0263	22273.7162	22578.2623
RLTR10A	6170.13024	6221.31879	6191.19312	7038.93597	7021.30605	7192.32026
RLTR10B	1720.45734	1777.24766	1721.70814	1828.57282	1799.9288	1957.09904
RLTR10B2	5870.86399	5863.59513	5895.21547	6239.26981	5897.40152	6069.60825
RLTR10C	15000.7969	14966.5753	15055.2628	16112.7848	15723.494	16289.2886
RLTR10D	3706.45816	3725.93257	3723.04416	4104.5199	3832.77693	3986.41954
RLTR10D2	1739.75918	1806.39262	1710.70357	1952.54069	1785.50484	1934.439
RLTR10E	1710.95453	1688.02462	1780.72558	1824.71697	1776.23243	1817.24302
RLTR10F	3275.83752	3305.51967	3204.2194	3572.01469	3398.06204	3527.54719
RLTR11A	23982.5691	24095.9366	23947.8505	24629.7045	24905.874	24884.0303
RLTR11A2	24457.5163	24516.6818	24664.9501	25301.4407	25822.0633	25824.7522
RLTR11B	8287.03249	8241.09553	8170.09153	8490.14921	8646.49388	8653.55459
RLTR11C_MM	7807.38319	7665.9886	7726.29284	8122.53391	8395.59428	8202.09944
RLTR11D	4375.67983	4470.18829	4374.25176	4657.17671	4776.18611	4747.16742
RLTR12A	6944.80346	7040.10433	6930.14282	7420.51575	7064.02986	7066.67947
RLTR12B	7690.96665	7582.39359	7494.64716	7479.6628	7526.64822	7647.51262
RLTR12B2	879.088812	839.193866	835.135972	804.604872	860.384681	842.245046
RLTR12BD_Mm	7120.61493	7075.0378	7001.78184	7033.50748	6951.2817	7087.37363
RLTR12C	2481.65539	2447.05811	2442.40668	2722.77703	2685.01743	2844.86885
RLTR12D	5556.11884	5574.36459	5667.03461	5622.89047	5691.09072	5770.78442
RLTR12E	5654.42939	5866.93804	5710.22839	5970.71497	6259.72502	6367.85393
RLTR12F	499.420881	463.10883	448.341438	431.141976	413.688809	413.469196
RLTR12G	2355.29776	2362.81	2399.93109	2455.62648	2448.74233	2566.61453
RLTR12H	1608.09022	1557.98036	1648.00288	1673.30909	1635.44226	1727.97092
RLTR13A	2248.78248	2298.64499	2294.13946	1980.60791	2023.72219	2082.01776

RLTR13A1	2410.37066	2545.91348	2398.3683	2256.40509	2209.75866	2182.88664
RLTR13A2	2575.99306	2577.83272	2651.07719	2307.1751	3430.88495	3017.86039
RLTR13A3	3514.94917	3513.76239	3552.80969	3112.80354	3146.53833	3124.67714
RLTR13B1	8927.07466	9105.11018	9163.45917	8818.83518	8593.73144	8556.86983
RLTR13B2	4922.87166	4914.27628	5010.17045	4881.47471	4480.88369	4662.76396
RLTR13B3	4381.84885	4471.32973	4455.45616	4326.5825	4273.84119	4334.81393
RLTR13B4	4092.37585	4140.22382	4054.71988	3992.77907	3683.381	3839.83488
RLTR13C1	5015.71361	5078.60264	4907.92485	4611.36057	4352.35769	4572.08987
RLTR13C2	11195.3094	11020.1567	11083.7491	10937.7222	10042.5927	10544.3582
RLTR13C3	3462.0289	3651.80635	3656.38818	3419.32295	3284.35924	3319.97963
RLTR13D	519.94163	517.313546	542.001946	499.451359	484.407805	470.297877
RLTR13D1	3351.11661	3257.02895	3265.15873	3220.12906	3029.70646	3273.46381
RLTR13D2	4698.39117	4641.69986	4685.66393	4353.88168	4218.75365	4250.29544
RLTR13D3	4995.02195	5037.62316	4944.05573	4639.03191	4570.74189	4589.28946
RLTR13D3A	2109.25481	2122.72491	2025.17067	1996.26935	2039.2151	2044.0374
RLTR13D3A1	6328.27663	6217.72234	6133.12656	5823.10793	5941.34188	5862.45359
RLTR13D4	2717.52604	2625.83347	2687.2087	2525.78579	2362.00414	2544.48447
RLTR13D5	9018.54513	8745.11578	8915.8535	8611.75512	8254.11923	8416.53652
RLTR13D6	12174.226	12048.2564	12193.6967	11462.6337	11111.5538	11338.4818
RLTR13E	7454.32533	7561.66306	7456.93211	7175.50632	7498.12422	7222.48616
RLTR13F	1289.54783	1309.15964	1318.59732	1248.15739	1293.46363	1338.12647
RLTR13G	6144.64419	6130.04829	6084.10641	5871.33394	5750.91897	5742.11979
RLTR14	16334.3672	16021.5378	15999.7215	16103.2571	16542.1322	16081.8116
RLTR14-int	13620.9509	13817.2578	13678.6268	14134.6141	14140.0363	14320.1889
RLTR14_RN	7550.80829	7512.76114	7453.82296	7559.40611	7328.93463	7435.4554
RLTR15	17982.1196	17701.64	18028.713	17549.5564	17640.3653	17749.168
RLTR16	11357.9226	11368.4945	11062.733	12495.996	12375.5107	12475.9295
RLTR16B_MM	5734.16263	5815.84153	5753.23658	5775.33427	5903.45853	6050.63479
RLTR16C_MM	3381.1407	3431.39792	3369.51397	3439.64105	3423.3907	3605.83104
RLTR17	19686.6183	19476.2833	19547.1603	20479.5534	20072.5397	20353.6149
RLTR17B_Mm	77138.0879	77742.1691	77639.6827	74124.594	76172.1668	74970.7764
RLTR17C_Mm	1920.23083	1854.88112	1822.93348	1749.98256	1810.77701	1797.14726
RLTR17D_Mm	11827.6418	11924.1668	11837.1816	10717.6341	10017.4525	10203.2422
RLTR18	5937.79942	6076.09012	6106.79809	6066.54067	6150.74959	6156.39256
RLTR18-int	3548.09421	3463.8861	3517.63556	3200.76153	3200.95739	3050.37272
RLTR18B	8242.49992	8146.97673	8114.90793	8355.28085	8532.77225	8438.82819
RLTR19	7110.27015	7168.18577	7123.79836	6763.28428	6929.87276	6990.49382
RLTR19-int	17889.2001	17762.4545	17542.8837	17035.075	16968.9924	16621.2463
RLTR19A	1101.84971	1056.58684	1056.4674	1082.57817	1158.10514	1090.63999
RLTR19A2	557.666633	539.355108	502.35852	553.092539	567.325928	543.493176
RLTR19B	2094.20318	2203.62403	2135.88209	2040.89407	2043.55718	1946.4244
RLTR19C	1899.06629	2025.98768	1991.61572	2023.30115	1840.20892	1878.65407

RLTR19D	408.026954	416.496067	453.831628	414.557462	421.647354	416.754474
RLTR1A2_MM	3752.282	3729.68546	3738.23021	3874.82044	3843.10374	3851.96519
RLTR1B	8478.03557	8499.79685	8320.99756	8834.8167	8596.11487	8570.92599
RLTR1B-int	9774.16186	9671.73654	9911.72424	10498.9775	10180.6968	10247.8752
RLTR1C	6153.73965	6329.07199	6317.68606	6349.3479	5798.94855	6036.30552
RLTR1D	3115.38699	3118.41391	3111.95034	3317.63926	3324.85873	3395.55124
RLTR1D2_MM	1855.04487	1834.23221	1852.2834	1986.34596	1995.38172	2036.28222
RLTR1E_MM	499.479075	498.130496	470.744037	485.733731	454.552318	475.094061
RLTR1F_Mm	548.825442	507.027821	520.39468	506.352842	500.567982	567.210133
RLTR20A	2121.71028	2151.78654	2156.69618	2206.71035	2287.38364	2364.23834
RLTR20A1	627.443575	646.620467	617.973933	664.548874	677.717329	704.8839
RLTR20A2	2643.3306	2555.1409	2548.86443	2701.18033	2774.66471	2792.62607
RLTR20A2B_MM	3508.72983	3455.07089	3489.89056	3722.4753	3693.74609	3834.99613
RLTR20A3_MM	6392.31446	6555.77004	6477.46842	6821.40716	6779.66858	6790.95148
RLTR20A4	28469.0459	28139.5618	28109.1465	27335.2394	27770.4175	27962.3264
RLTR20B1	4647.71475	4738.27367	4666.42191	4949.621	4927.50118	4986.20358
RLTR20B2	6584.6953	6683.68745	6524.34112	7109.5518	7016.51792	7262.75063
RLTR20B3	7429.45005	7312.10673	7534.56261	7735.72983	7569.79123	7908.46856
RLTR20B3A_MM	4998.52456	4951.90717	4972.70948	5238.0027	4968.54212	5270.96259
RLTR20B4_MM	3071.84003	3194.73887	3050.93667	3272.09906	3347.11603	3595.09358
RLTR20B5_MM	6911.34963	7043.6132	7016.93874	7253.71208	7116.10855	7101.82352
RLTR20C	1591.89675	1585.32765	1505.62555	1578.98866	1584.09457	1601.0184
RLTR20C1_MM	14293.2594	14112.1142	14026.9237	14219.7537	14189.4162	14389.4828
RLTR20C2_MM	12863.9511	12710.7902	12816.1027	13274.4518	13782.9804	13849.0018
RLTR20D	5241.89953	5300.40291	5284.29452	5771.73822	5463.45477	5652.09071
RLTR21	19212.6353	19153.574	19309.1012	18755.3566	21718.9711	20516.5657
RLTR22_Mur	21358.9959	20968.3655	21452.3718	18671.9855	17080.6424	18158.79
RLTR22_Mus	7056.63582	7032.5872	6932.30299	6506.99633	6176.32622	6435.8781
RLTR23	12477.2869	12571.9424	12592.2462	13314.2445	13062.0939	13260.3862
RLTR24	10421.6827	10326.6783	10337.2126	9946.28038	10118.992	10212.9294
RLTR24B_MM	3047.42243	3302.49571	3151.62877	3109.23879	3124.82554	2941.19234
RLTR25A	11827.6706	11998.8623	12112.4994	11592.7592	11141.955	11588.8447
RLTR25B	19169.1748	18667.6993	18990.8169	18500.8054	18314.3817	18614.7151
RLTR26	3213.32059	3261.02738	3252.84761	3721.16063	3431.75215	3840.23009
RLTR26_Mus	11068.7652	11109.7158	11294.2216	13184.7212	12133.4739	13398.707
RLTR26B_MM	4749.00941	4832.72589	4797.91891	5307.31107	5159.42871	5473.01652
RLTR26C_MM	4398.44181	4279.73708	4360.65485	4965.65179	4750.14092	4893.71256
RLTR26D_MM	2384.88566	2423.30292	2405.99385	2655.01098	2631.3653	2656.96685
RLTR27	5089.53105	5055.34146	5030.40269	5910.4051	5558.53313	5991.03508
RLTR28	19119.2313	19244.9298	18918.1319	20257.0708	20748.3786	20658.4226
RLTR28B	3248.16528	3101.51435	3037.03922	3150.24472	3094.78712	3182.20047
RLTR3_Mm	152.683032	157.063483	173.499178	146.685818	155.093196	149.580122

RLTR30	2716.59023	2643.71075	2689.44691	2804.83437	3098.54581	2875.42996
RLTR30B_MM	2339.73498	2446.31977	2420.75231	2469.26733	2401.75493	2514.00289
RLTR30C_MM	973.92401	989.564939	1010.14868	962.843086	871.613904	1008.56369
RLTR30D_MM	2317.87001	2288.68885	2278.02735	2353.70573	2301.62773	2336.22933
RLTR30D_RN	457.393243	441.149689	440.875136	437.228959	443.907705	489.580919
RLTR30D2_MM	6244.75873	6095.02692	6156.47877	6552.51049	6250.47511	6487.16326
RLTR30E_MM	2741.83355	2747.38826	2723.60014	2815.39828	2622.67993	2824.46047
RLTR31_Mm	1296.18501	1323.44755	1298.2052	1318.7704	1356.12381	1319.95608
RLTR31_Mur	3166.61756	3090.33182	3142.07485	3214.4157	3096.8047	3147.13703
RLTR31A_Mm	1648.78623	1592.7577	1659.42942	1647.96082	1706.68632	1679.01293
RLTR31B_Mm	2965.68912	3001.34835	2956.83216	3101.83812	3044.04471	3165.72847
RLTR31B2	9167.34966	8993.27086	9023.23642	8942.90024	8907.23411	8776.16286
RLTR31C_MM	4904.98796	4809.2938	4810.28857	4791.36237	4729.64294	4824.98765
RLTR31D_MM	23886.2927	23709.4744	23962.9752	23296.9107	23506.5956	23877.7837
RLTR31M	2325.48967	2341.83141	2340.14402	2299.44775	2316.5117	2386.92522
RLTR33	16442.8584	16587.3455	16350.216	17376.6367	21424.7425	19164.0401
RLTR34B_MM	4853.587	4952.80631	4819.61108	5332.53512	5354.79776	5440.31317
RLTR34C_MM	3606.38085	3550.17195	3670.94247	3754.78386	3625.30842	3808.54669
RLTR34D_MM	3929.87544	4025.366	4035.54291	4253.89386	4168.33148	4368.79106
RLTR35B_MM	3243.84849	3365.92349	3352.64866	3409.67501	3357.1572	3504.56745
RLTR4_Mm	1220.6181	1254.79211	1238.82433	1427.28735	1404.70278	1423.12578
RLTR4_MM-int	11648.2496	11606.3751	11599.8424	14185.1676	13670.5209	14398.6698
RLTR40	20888.3357	20780.3759	21090.8086	19218.3752	18443.5714	18715.1441
RLTR41	8529.73693	8638.73853	8688.3893	9456.90035	9324.43058	9624.55947
RLTR41A2	3516.48265	3637.27908	3693.20377	3977.63772	3917.19063	3943.98197
RLTR41B	701.646355	734.541578	739.407956	696.368146	691.190217	721.197825
RLTR41C	1119.93106	1101.89241	1131.46559	1173.33812	1097.88855	1150.48744
RLTR42	1316.83945	1251.03922	1230.92892	1340.34058	1306.77547	1338.54448
RLTR42-int	17085.3306	17258.6965	17030.1124	16482.0133	16615.3786	16560.6257
RLTR43A	2.59720478	7.02037208	2.60443524	6.83558424	7.59513665	0
RLTR43B	5.22062278	4.24498908	8.81281828	3.86392068	5.73676514	3.04815745
RLTR43C	1763.79669	1713.25174	1761.69424	1834.38814	1852.46699	1862.12583
RLTR44-int	6110.97018	6248.4173	6337.43649	6354.77444	6168.82464	6434.57138
RLTR44A	1622.83358	1624.84036	1654.94804	1788.24581	1686.24301	1745.25918
RLTR44B	1457.90636	1391.69564	1461.94131	1634.11188	1598.59813	1645.89462
RLTR44C	1918.84101	1938.31403	1929.48976	2013.87841	1886.66516	1995.91896
RLTR44D	813.358142	818.787864	814.375466	863.766481	876.118236	873.875751
RLTR44E	985.55953	1003.6068	1010.94184	1159.27535	1080.66003	1120.74966
RLTR45	11027.7279	11003.1062	11188.9632	12064.6393	11667.7538	12023.3647
RLTR45-int	10821.335	10667.4191	10883.2018	10160.47	9851.852	10061.9543
RLTR46	274.47537	302.862212	285.10012	321.016446	288.067027	349.45789
RLTR46A	399.357197	431.517459	383.688178	463.399795	427.484998	474.346718

RLTR46A2	366.589637	365.474992	354.319034	344.246429	347.192171	356.1764
RLTR46B	490.120435	436.660271	461.529988	494.462397	473.803299	521.023657
RLTR47	1642.26701	1643.45304	1636.73341	1496.71701	1335.09467	1481.73542
RLTR47_MM	122.395766	133.389295	125.7069	127.160451	103.926597	126.778222
RLTR48A	6098.45127	6173.72548	6321.54994	6074.46621	5788.89587	6045.59893
RLTR48B	863.452109	861.808122	816.299515	913.035016	832.346237	838.48602
RLTR48C	1458.93497	1457.1644	1503.84217	1460.62278	1552.38193	1466.05028
RLTR49	6775.05195	6774.22113	6766.13468	6791.16615	6737.47054	6771.28995
RLTR5_Mm	1445.54316	1461.65372	1517.13141	1722.15949	1460.95832	1697.62767
RLTR50A	624.392882	630.622171	557.424378	588.426312	541.7949	651.718986
RLTR50B	2823.27799	2819.79446	2839.3748	3114.27519	2826.1309	3059.5927
RLTR51A_Mm	10773.6558	10769.7002	10911.8127	11189.9765	11225.0864	11447.0821
RLTR51B_Mm	4519.99823	4498.67652	4569.52737	4996.97371	6667.75606	6024.02726
RLTR53_Mm	3556.2009	3656.87019	3657.34492	4015.02155	4091.96143	4176.33707
RLTR53B_Mm	794.12184	787.848066	798.017041	855.619531	825.782996	801.325455
RLTR6-int	36059.1079	36391.9581	37128.6875	42182.3415	40214.782	44819.3678
RLTR6_Mm	1728.17032	1705.65787	1746.82796	1803.74416	1810.57342	1918.46709
RLTR6B_Mm	4810.52919	4779.17406	4811.63573	5110.35822	4899.1145	5349.05238
RLTR6C_Mm	524.729735	570.538423	533.573937	582.720047	500.949613	564.619504
RLTR8	1936.87674	1912.02778	1856.8754	1886.29885	1828.83627	1898.55222
RLTR9A	4522.67407	4572.0658	4582.58888	5033.26992	4955.57179	5132.77202
RLTR9A2	1742.51366	1811.53735	1742.40016	1806.3669	1802.35234	1840.15336
RLTR9A3	5302.82741	5187.26459	5316.37808	5536.57859	5523.70781	5579.18522
RLTR9A3A	1459.33446	1513.90007	1493.10746	1556.68554	1419.04591	1497.50924
RLTR9A3B	2360.15874	2229.33758	2293.86744	2315.67196	2127.08179	2331.49901
RLTR9A4	207.538367	208.656443	225.416545	206.471811	174.928787	198.715956
RLTR9B	1679.38701	1629.24877	1703.53348	1857.13312	1749.48902	1857.74107
RLTR9B2	1153.82579	1139.60744	1143.99514	1259.96628	1141.56089	1328.85331
RLTR9C	1688.1957	1671.12647	1724.28686	1768.93	1576.88472	1813.19418
RLTR9D	5897.39858	5950.53478	6034.56358	6859.6088	6519.69467	6849.19482
RLTR9D2	1554.015	1564.92125	1536.99805	1790.24795	1849.29658	1959.97949
RLTR9E	9384.36777	9498.02177	9330.8392	10550.8745	9943.50466	10364.7237
RLTR9F	3057.06889	3230.16889	3232.16705	3566.62703	3470.78283	3623.17858
RLTRETN_Mm	8852.44146	8888.45828	8920.99172	10055.371	10028.9959	10019.4205
RMER10A	30930.1548	31063.7885	30403.6794	34431.728	36041.4304	36314.42
RMER10B	18458.3711	18477.3242	18199.1966	21230.952	21864.1756	22031.9454
RMER12	21496.0721	21509.8553	21486.4794	20386.7708	20077.1358	20096.8097
RMER12B	9093.6465	8943.14407	8873.0402	8595.04065	8258.98636	8492.54944
RMER12C	19240.8035	19372.5917	19201.7625	18731.4472	18388.6291	18317.738
RMER13A	12806.161	12746.557	13030.3542	14757.1455	14430.573	15080.6363
RMER13A1	11793.1992	11796.3363	11900.2101	13918.3726	13617.3587	14166.2889
RMER13A2	16115.0549	16033.9475	16506.0889	18174.8617	17613.6547	18465.0027

RMER13B	27785.9711	28181.8892	28189.7179	33944.0525	32683.8161	34675.0802
RMER15	114317.092	114033.247	114273.929	116397.64	115087.801	116699.318
RMER15-int	28240.6177	28147.5856	28106.5922	28707.78	28523.4463	29234.6636
RMER16	3854.74681	3813.93205	3934.0248	3788.82522	3956.03717	3868.64443
RMER16-int	30654.0165	31089.8234	30893.1642	27239.7402	26233.3803	26107.4424
RMER16_Mm	3638.3206	3665.11391	3721.80826	3640.37762	3640.70045	3556.6729
RMER16A2	1517.1954	1447.20391	1543.17143	1557.9035	1492.22916	1513.56019
RMER16A3	2186.50776	2122.80439	2174.38534	2102.37545	1964.79637	2042.42465
RMER16B	761.381541	785.317921	755.862742	751.887247	822.468548	715.075163
RMER16B2	2324.13917	2234.80825	2297.12654	2134.02881	2159.98431	2085.72763
RMER16B3	652.35923	642.703339	661.458328	646.268974	580.65783	563.036145
RMER16C	1358.25632	1267.03842	1316.14067	1248.00566	1213.59559	1246.69846
RMER17A	17509.9437	17374.3666	17906.6274	20775.5295	19124.7676	21021.0794
RMER17A-int	3216.92124	3201.18928	3214.74776	3185.24948	2988.83867	3145.44828
RMER17A2	20037.0664	19923.8766	20635.7149	24198.2598	23004.6717	25089.9543
RMER17B	37355.7952	37373.0273	37965.2577	46081.3317	44762.9007	47603.4245
RMER17B2	9053.65353	9122.49455	9270.33394	10892.7898	10569.1092	11226.1469
RMER17C	39143.9917	39356.2197	39231.4788	45832.8573	47941.7455	49097.8633
RMER17C-int	15709.2944	15767.2382	16024.5222	15564.2203	15012.002	15360.6498
RMER17C2	15740.9363	15603.2309	15817.645	17515.2957	17606.4283	17981.1057
RMER17D	8661.72988	8716.37017	8931.00607	10054.0312	9372.45894	10262.5055
RMER17D2	14039.8982	13864.682	14131.5351	16451.0529	15390.7482	16829.0491
RMER19A	24501.7857	24522.471	24737.8209	25057.0229	24785.8286	25185.9722
RMER19B	74784.0028	75392.458	75011.1576	77595.4282	76775.015	78268.9908
RMER19B2	26227.6681	26061.8427	25916.2407	24977.564	23706.2041	24031.1866
RMER19C	28044.3499	28030.607	27945.5656	26827.7923	25861.2563	26719.2273
RMER1A	50020.7314	50270.9272	50565.7936	60803.6326	60057.0198	62342.1898
RMER1B	80890.0108	81213.2563	82249.0352	94730.4047	93810.8618	96591.786
RMER1C	40985.3228	40696.7382	40862.6752	43152.111	41918.168	44083.069
RMER2	19763.6768	19937.0233	19745.3731	21236.3865	20766.3302	21199.3657
RMER20A	10989.6312	11118.5328	11080.3286	11707.8395	11283.8998	11932.0649
RMER20B	15361.9342	15312.8761	15627.709	16304.7177	15429.9521	16188.6867
RMER20C_Mm	13411.4942	13346.4828	13266.6483	15143.4939	14554.681	14989.1024
RMER21A	5236.08544	5338.11906	5085.56924	6276.40326	6350.94383	6480.86629
RMER21B	7888.50049	7951.45977	8060.67305	8901.28656	9103.39135	9234.56352
RMER30	15168.3067	15245.6111	15176.5497	15276.0856	15474.9681	15046.7651
RMER3D-int	22655.5233	22267.6567	22408.7441	19836.9254	19302.9874	19489.5337
RMER3D1	3.96134022	3.10202958	0	7.47182782	2.86838257	9.14447234
RMER3D2	12.3957025	10.2858261	6.17554117	9.9779235	23.795421	8.12402874
RMER3D3	24.8700446	22.3677026	20.1643881	28.8708377	23.997179	18.2230778
RMER3D4	3.96134022	6.85714996	6.20838304	4.58550211	4.40345286	9.14447234
RMER4A	18677.9865	18483.0336	18353.7537	18943.504	20762.7096	20102.1222

RMER4B	39668.5468	39469.3878	38887.5964	39972.3932	42032.399	41377.6452
RMER5	55137.9379	55526.5152	55160.0071	52248.9689	52828.9267	52665.8313
RMER6-int	5393.88214	5211.50429	5419.14266	4715.87557	4326.0974	4604.94826
RMER6A	43925.1678	43822.332	43790.9227	42870.6803	42916.8733	43248.6383
RMER6B	10344.5928	10090.8382	10155.6916	9831.14878	11213.2021	10629.6078
RMER6BA	5307.71513	5297.46188	5325.58067	5321.15475	5216.61649	5263.36245
RMER6C	46586.2138	45912.8938	46814.3236	42972.6347	39414.0787	41970.9263
RMER6D	18579.4657	18009.9699	18643.2343	16918.6724	15730.5653	16654.6048
RNERVK23-int	208.456878	232.1667	219.70079	217.931776	251.081912	240.448757
RNLTR23	21.6038787	21.8778339	19.4932942	32.4787448	17.573704	28.3353003
RodERV21-int	22202.8621	22241.6327	22747.7001	23404.2922	23625.408	24011.3468
RSINE1	483548.744	484870.253	477925.06	505027.472	535860.962	522925.523
SRV_MM-int	2127.21243	2113.91	2139.35683	2027.20395	1956.07212	2024.75131
SSU-rRNA_Hsa	4720.81343	4733.05855	4569.60668	6472.14047	11269.6271	8364.2375
SUBTEL_sa	87.7272237	90.7767895	66.4245558	92.8971915	90.0903004	91.2207761
SYNREP_MM	185154.106	185187.538	186757.029	180965.545	338365.794	271905.114
T-rich	51829.1933	51214.3216	51708.0294	46480.6777	47143.9642	46474.5556
Tigger1	8781.51376	8681.43103	8820.54377	8153.75435	8165.3428	8056.24541
Tigger10	1571.32305	1631.37045	1600.23198	1493.78422	1698.58862	1557.97229
Tigger11a	505.218719	492.007744	452.470855	460.11001	424.352865	422.439368
Tigger12	874.733226	916.66603	898.930379	834.24375	866.806935	873.250016
Tigger12A	502.555457	463.191048	487.31377	435.824674	504.304168	483.734898
Tigger12c	580.595327	588.497308	561.044747	531.491556	609.949371	535.316454
Tigger13a	4240.31114	4281.93886	4336.0162	3989.69456	4101.31222	4004.76826
Tigger14a	1179.48276	1099.52488	1094.29523	1084.99514	1093.79323	1030.52957
Tigger15a	4078.51797	4084.7114	4041.90341	4063.28071	4205.48374	4037.93523
Tigger16a	1680.11678	1643.86008	1574.92161	1605.3259	1695.64185	1560.35873
Tigger16b	982.555496	945.647567	948.458947	948.497283	1044.82836	951.498379
Tigger17	1515.27397	1538.22543	1552.78671	1555.12195	1608.61803	1510.58094
Tigger17a	7508.58091	7601.98813	7516.87066	7192.06663	7530.54423	7299.83264
Tigger17b	467.584939	408.170525	474.512194	403.116936	432.352739	406.773986
Tigger17c	3769.81497	3960.22085	3820.82543	3647.06148	3803.79474	3816.20355
Tigger18a	1646.41918	1713.41314	1645.18497	1526.13447	1564.34163	1449.71001
Tigger19a	3658.70556	3712.13655	3692.7254	3690.57827	3821.75496	3747.79785
Tigger1a_Art	167.689046	165.39034	143.168327	138.922888	140.468089	144.909086
Tigger1a_Mars	10.4674588	6.0410394	2.60443524	5.30708354	3.87839363	10.1385692
Tigger20a	1151.69413	1150.05882	1088.366	1095.07405	1138.2495	1029.24261
Tigger2b	1092.80406	1052.74738	998.672866	960.200915	1037.21195	984.527523
Tigger5	11000.6061	10881.2981	10935.7732	10589.9825	10545.6918	10650.0897
Tigger5b	11143.595	11164.3209	11264.9488	11119.9394	11116.6968	10874.5366
Tigger6a	2937.13402	2860.4478	2970.28128	2750.22745	2846.39128	2746.94982
Tigger6b	1320.85217	1316.75281	1243.73049	1195.83894	1168.7127	1186.82821

Tigger7	30358.5386	30353.3232	30363.922	29101.8031	29940.9117	29607.0906
Tigger8	811.954162	792.826746	795.27411	764.708339	719.551962	705.585136
Tigger9a	1180.33521	1158.38406	1099.91895	1117.74554	1140.02826	1116.60861
Tigger9b	2277.6988	2198.48051	2292.19683	2182.70328	2172.67328	2259.35051
tRNA-Ala-GCA	2057.34951	2085.33551	2068.76289	2203.11333	2268.016	2240.63697
tRNA-Ala-GCG	519.797458	500.08906	544.79414	506.640153	561.388626	526.675617
tRNA-Ala-GCY	32.7140853	40.6539417	36.1194182	42.0764383	36.1574191	37.5719862
tRNA-Ala-GCY_	7160.35939	7228.10467	7183.07231	7431.31354	8215.65799	7862.0718
tRNA-Arg-AGA	74.0460254	76.8173529	67.5225939	74.3456287	59.6295389	72.0431215
tRNA-Arg-AGG	22.0432121	25.6331566	26.6355061	23.4784163	14.8870258	13.1735533
tRNA-Arg-CGA	9.85092411	10.122604	8.84566015	9.00032853	11.8369389	11.1853596
tRNA-Arg-CGA_	4.65651455	9.79615978	9.68096336	2.25008213	5.57511453	5.08904466
tRNA-Arg-CGG	13.7860511	13.7147046	5.27455422	12.4840192	8.12019588	5.08904466
tRNA-Arg-CGY_	55.5573246	50.7762422	53.9092641	52.4345918	54.9428923	54.7746204
tRNA-Asn-AAC	91.3934032	95.3479193	91.1267205	121.643813	75.5065221	98.2058008
tRNA-Asp-GAY	86.3169531	96.4912835	88.268843	83.5981805	77.3849473	78.5376845
tRNA-Cys-TGY	281.833942	251.922545	266.474971	279.405575	245.809434	221.909518
tRNA-Gln-CAA	28.9100244	33.9596091	29.2070994	37.8711662	23.8355284	17.7523761
tRNA-Gln-CAA_	773.049041	790.544064	790.999069	180.98319	39.0659091	42.5819906
tRNA-Gln-CAG	69.5467902	80.1641144	57.4475282	68.992086	58.1345761	68.9686173
tRNA-Glu-GAA	15.1239733	12.2450986	20.7134071	23.7344298	15.8769831	12.166283
tRNA-Glu-GAG	15.6618683	20.0818847	13.2849112	8.72487566	14.0186116	11.1853596
tRNA-Glu-GAG_	101.132659	111.348644	102.379765	74.6869801	99.1803999	81.1875939
tRNA-Gly-GGA	201.976973	218.452198	202.713406	231.346931	221.226425	199.762747
tRNA-Gly-GGG	51.0780113	63.5111082	42.4591687	42.8601279	50.196695	69.9758875
tRNA-Gly-GGY	149.180423	125.389692	130.521668	103.131129	130.934366	92.6921613
tRNA-His-CAY	5.91579711	1.30618162	5.24171235	5.39242139	7.11018482	3.04815745
tRNA-His-CAY_	44.060211	57.47017	59.2166602	53.4121868	54.9027849	43.628781
tRNA-Ile-ATA	50.2187424	58.1232608	53.1068028	63.1340967	56.7611564	55.7555439
tRNA-Ile-ATT	95.0333694	99.4303945	86.4340272	108.694226	113.360662	105.892061
tRNA-Leu-CTA	5.91579711	7.67366524	8.91134388	3.86392068	8.4434971	8.09768199
tRNA-Leu-CTA_	13.11709	16.6533097	10.5491084	9.34167991	16.2403918	11.1326661
tRNA-Leu-CTG	46.6574158	57.7966142	56.3823319	68.2705046	51.5494505	68.4452221
tRNA-Leu-CTY	27.545889	25.4697321	13.317753	20.6774284	19.2704249	22.2916789
tRNA-Leu-TTA	39.2202039	38.2049017	37.6679498	55.2355797	45.0858681	33.5165585
tRNA-Leu-TTA(m)	797.321949	797.237486	795.095644	180.890271	50.1347024	40.5147567
tRNA-Leu-TTG	63.5261402	55.1844534	67.4897521	51.751889	68.9213965	48.730999
tRNA-Lys-AAA	54.9869251	62.9398308	51.1406195	58.6339325	81.2232335	62.8327823
tRNA-Lys-AAG	446.84662	411.597481	422.749743	440.437196	479.580172	466.36101
tRNA-Met	45.1622142	45.7147378	46.8655779	78.9311308	43.7926632	58.790528
tRNA-Met-i	46.44771	45.3882936	48.4376587	52.481051	55.9127959	57.862298
tRNA-Met_	102.903623	128.001954	118.925948	102.619101	96.9786735	113.565148

tRNA-Phe-TTY	99.5257894	108.164498	72.6657807	105.718772	107.401475	95.8588791
tRNA-Pro-CCA	29.6576252	26.7759138	32.9095728	16.0065885	28.5622825	29.3689172
tRNA-Pro-CCG	18.363926	19.265673	16.8560171	9.17100422	17.573704	11.1458394
tRNA-Pro-CCY	77.6335653	91.5931024	81.1830222	77.7828602	118.672637	108.489277
tRNA-SeC(e)-TGA	7.22750611	9.4695132	10.6147922	6.7502464	6.42347498	11.1063193
tRNA-Ser-AGY	51.7207592	46.5312531	38.8223791	32.8200962	38.1373339	31.4493246
tRNA-Ser-TCA	4.57787489	1.95927243	3.50542219	0.80691928	1.85837151	0
tRNA-Ser-TCA(m)	9.15574978	6.20405915	2.20320457	3.69324499	6.94853421	8.09768199
tRNA-Ser-TCA_	12.4743421	13.5512802	14.1858981	10.6995049	11.6752883	14.1939969
tRNA-Ser-TCG	10.5460984	12.4083207	9.7466471	18.3420084	13.856961	13.1999
tRNA-Ser-TCY	37.3181735	40.0005473	45.293497	62.1565018	32.6023267	31.4888447
tRNA-Thr-ACA	30.8120549	31.3475494	40.2395432	30.1044462	26.8655616	33.5165585
tRNA-Thr-ACG	9.87713733	7.02057443	14.1530562	10.4046127	3.55509241	4.06860106
tRNA-Thr-ACG_	45.909815	47.5109905	32.7782054	46.2352511	46.3176909	33.4506917
tRNA-Thr-ACY	44.6505324	39.0213158	45.2278133	46.7083996	49.2875629	31.4756713
tRNA-Thr-ACY_	31.4285896	33.1433974	38.2897809	44.8385475	59.831297	36.4725024
tRNA-Trp-TGG	38.039561	47.0211218	39.0194303	43.736736	35.5108167	39.5206598
tRNA-Tyr-TAC	52.5469997	64.3275223	43.2616301	51.2474425	47.3477556	38.63195
tRNA-Tyr-TAT	8.51300189	9.4695132	8.84566015	7.55716567	16.9271016	9.10495222
tRNA-Val-GTA	42.0009012	39.1842344	27.5693349	35.3650705	34.9456501	31.462498
tRNA-Val-GTG	72.8391693	97.3072929	89.9301567	82.3451327	82.7783575	75.0385855
tRNA-Val-GTY	60.4696801	45.8783646	49.6999062	33.6270155	51.1860418	39.5470066
U1	1393.06799	1374.9595	1396.37777	1401.41043	1410.84428	1394.30356
U13	52.5732129	47.3477684	54.8102511	35.9624355	42.5407867	40.5542768
U13_	80.1715283	83.2663464	68.8319398	65.1281653	89.9687572	76.9740857
U14	14.428799	14.3675931	14.1858981	16.0065885	10.4635192	8.13720211
U17	124.533715	111.348644	111.126899	88.7772573	107.380811	148.06263
U2	2130.00623	2213.82773	2154.19091	2103.13212	2044.87104	2017.4339
U3	251.310233	237.553941	214.883858	223.712008	211.066153	211.418808
U4	362.884138	375.67951	349.08445	376.445931	368.279639	343.914857
U5	213.946448	235.921618	230.937414	190.496033	237.003139	214.141171
U6	4132.85481	4075.73348	3964.48702	3996.4173	4253.90228	4047.69885
U7	114.636656	120.083253	118.236269	95.1240441	92.5746101	107.992229
U8	25.0273239	38.3679215	22.0648876	22.5472805	28.2389813	23.364816
UCON1	90.4817078	74.4503287	77.1050317	66.3617738	52.7612195	76.0985492
UCON10	300.460001	313.311362	307.369187	315.592726	313.539726	267.6714
UCON11	274.389915	235.595275	282.02877	266.483008	232.901713	219.085308
UCON12	110.367049	106.77711	120.596555	100.34579	117.521029	107.389793
UCON12A	110.000063	116.736795	128.344177	111.153862	122.692627	91.2998163
UCON13	254.41912	260.656244	264.25751	228.638862	253.990402	234.220709
UCON14	308.081229	296.330292	300.858099	300.377896	243.890291	295.012604
UCON15	169.53865	174.206358	194.604523	158.964073	191.634077	156.120792

UCON16	104.929905	131.91979	128.114284	103.131129	105.664036	113.538802
UCON17	152.827205	133.715942	148.344356	122.404273	116.673889	110.490644
UCON18	102.523008	98.4504548	104.9842	107.107407	91.5852632	88.3175257
UCON19	116.125566	136.654345	120.300978	115.444472	111.663941	121.636484
UCON2	390.496084	423.026773	410.347234	360.594369	435.888388	405.700849
UCON20	243.381261	235.921719	230.198472	217.846439	210.602476	186.987442
UCON21	135.053601	111.675291	106.621964	101.175939	134.651109	113.578322
UCON22	125.045397	136.737069	120.972072	144.548094	90.251951	115.434782
UCON23	100.332632	88.0012042	105.148409	118.035906	133.560883	101.319825
UCON24	107.455285	82.9401045	97.9405136	85.2082288	79.768378	67.921827
UCON25	105.369762	88.4912752	103.346435	95.0619358	76.5165331	82.1026505
UCON26	652.182553	597.07001	653.152394	567.919559	596.233397	637.907017
UCON27	389.71493	418.536546	437.409684	402.391564	382.984961	375.140234
UCON28a	525.431201	542.048677	503.179567	473.552185	482.771856	520.664929
UCON28b	318.561794	314.289986	302.545126	305.614803	301.701566	299.054858
UCON28c	211.454096	211.920177	211.296331	187.361762	188.462447	196.661896
UCON29	819.260308	841.889662	863.711948	884.354293	798.854221	844.486581
UCON31	503.735576	516.252703	477.632806	537.1016	478.205531	482.727628
UCON4	341.864289	367.270435	362.048071	355.070151	368.725705	343.704083
UCON5	293.684935	274.451649	239.471076	256.407888	239.607161	320.181186
UCON6	231.372466	252.411402	235.425927	269.311016	230.235088	249.316589
UCON7	480.820512	448.331765	451.339975	432.076902	414.498283	378.346472
UCON8	288.726444	343.189204	308.605721	301.960437	335.112146	302.965379
UCON9	159.609086	163.512375	166.870979	150.902461	181.292101	156.186659
URR1A	97647.8755	97728.0106	97345.1284	96303.3365	100352.049	97787.7069
URR1B	91390.1779	91801.5494	90658.6313	91080.9653	94727.5503	92120.8299
X1_LINE	306.7302	272.493186	283.126809	254.421888	276.371075	262.503315
X2_LINE	174.968979	187.10475	180.418625	167.196361	162.485353	146.963147
X3_LINE	980.128677	1004.5033	980.033461	1062.9177	936.718289	967.413563
X5A_LINE	276.61332	298.044428	311.719205	229.10443	226.901808	267.645054
X5B_LINE	248.175133	257.472704	228.593549	259.294214	208.257931	236.222076
X6A_LINE	624.58319	535.682104	580.78219	500.141642	510.60671	541.830777
X6B_LINE	1029.09443	1020.26102	1069.35821	1037.78278	1069.06679	1013.63297
X7A_LINE	628.269815	614.537912	605.550039	634.917576	643.398838	605.516289
X7B_LINE	650.069769	653.886988	664.60249	630.626966	627.885263	617.014269
X7C_LINE	621.468012	593.478314	617.729783	640.279188	644.531613	662.30191
X7D_LINE	34.0520076	35.5921338	32.7125216	29.7242161	31.7539663	22.3180256
X8_LINE	659.566815	675.764518	686.808037	628.105222	613.745719	605.980404
X9_LINE	183.26546	194.942244	175.709511	148.749575	182.92744	174.515124
YREP_Mm	5093.94588	5193.45914	5126.41202	5271.40121	5259.88776	5535.93188
Zaphod	4783.01999	4907.98636	4745.88226	4343.52255	4408.93836	4365.89538
Zaphod2	1011.92425	983.767519	943.005927	935.900418	884.502793	873.009848

Zaphod3	2766.61814	2735.54696	2766.35489	2459.42781	2603.89629	2348.1291
ZP3AR	12170.9504	12406.4664	12386.1723	16805.1535	17762.9958	18811.34
(CTACG)n	0.64274789	1.95927243	0	2.16474429	2.70673196	3.06133082
CENSAT_MC	1.28549578	0.65309081	0	0	0	1.02044361
(CACGA)n	0	0.81631292	0.90098695	1.44316286	0.84836045	0
	#DIV/0!	#DIV/0!	#DIV/0!	#DIV/0!	#DIV/0!	#DIV/0!

Table S2 | List of siRNAs

Target	Reference	Company	Type
Non-targeting	D-001810-10	Dharmacon	ON-TARGETplus SMARTpool
siTop2α	L-043916-01-0005	Dharmacon	ON-TARGETplus SMARTpool
siTop2β	L-042134-00-0005	Dharmacon	ON-TARGETplus SMARTpool
siDNAPK	L-040958-00-0005	Dharmacon	ON-TARGETplus SMARTpool
siRad51	L-062730-00-0005	Dharmacon	ON-TARGETplus SMARTpool
siRad52	L-043751-00-0005	Dharmacon	ON-TARGETplus SMARTpool
siErcc1	L-045170-01-0005	Dharmacon	ON-TARGETplus SMARTpool
siSLX4	L-057081-01-0005	Dharmacon	ON-TARGETplus SMARTpool

Table S3 | List of antibodies

Antibody/Host	Company/Reference	Dilution/Use
γH2AX - Mouse	Abcam/ab22551	IF & WB (1:1000)
γH2AX - Rabbit	Abcam/ab2893	IF (1:1000) & ChIP (5μg/IP)
pH3S10 - Rabbit	Merck/06-570	IF & WB (1:1000)
Top2α – Mouse	Santacruz/sc-365916	IF (1:200) & WB (1:2000)
Top2β - Rabbit	ThermoFisher/ PA5-54984	IF (1:200) & WB (1:2000)
53BP1 - Rabbit	Novus Biologicals/100-304	IF (1:1000)
Brca1 - Mouse	From Dr. Andre Nussenzweig	IF (1:200)
RPA32 - Mouse	Novus Biologicals/600-565	IF (1:250)
Rad51 - Rabbit	Calbiochem/ PC130	IF (1:100)
Ercc1 – Mouse	Santacruz/sc-17809	IF (1:200)
HP1α - Mouse	Euromedex/ 2HP1H5	IF (1:200)
H3K9me3 - Rabbit	Abcam/ ab8898	IF (1:200)
Lamin A – Rabbit	Abcam/ab26300	WB (1:5000)
GAPDH – Mouse	Millipore/MAB374	WB (1:10000)
Tubulin – Mouse	Merck/T6074	WB (1:30000)

H3 - Rabbit	Abcam/ab1791	WB (1:20000)
H4	Abcam/ab7311	WB (1:20000)
GFP - Mouse	Santacruz/sc-9996	WB (1:5000)
HRP GαR	ThermoFisher /PI-1000	WB (1:5000)
HRP HαM	ThermoFisher /PI-2000	WB (1:5000)
Alexa 488 DαR	ThermoFisher / A-21206	IF (1:500)
Alexa 488 DαM	ThermoFisher / A-21202	IF (1:500)
Alexa 568 DαR	ThermoFisher / A10042	IF (1:500)
Alexa 568 DαM	ThermoFisher / A10037	IF (1:500)
Alexa 647 GαR	ThermoFisher / A-21244	IF (1:500)
Alexa 647 DαM	ThermoFisher / A-31571	IF (1:500)

*IF (Immunofluorescence), WB (Western Blot), ChIP (Chromatin Immuno-precipitation)

Table S4 | List of plasmids

Top2αWt-YFP	CMVp-humanTop2αWt-YFP
Top2αY805F-GFP	Gift from Dr. Andy Porter
Major Satellite repeats	Gift from Dr. Maria-Elena Torres Padilla
pmRFP-Sumo1	Gift from Dr. Steve Jackson
pmRFP-Sumo2	Gift from Dr. Steve Jackson
pmRFP-Sumo3	Gift from Dr. Steve Jackson
SLX4WT-GFP	Gift from Dr. Alfred Vertegaal & Dr. Roman Gonzalez Prieto
SLX4ΔSIM-GFP	Gift from Dr. Alfred Vertegaal & Dr. Roman Gonzalez Prieto

Table S5 | Primers for mouse genome

Name	Sequence
Major Satellites FW	GACGACTTGAAAAATGACGAAATC
Major Satellites RV	CATATTCCAGGTCCTTCAGTGTGC

*Primers were used for ChIP-qPCR & Top2cc's isolation qPCR assay. FW (forward primer), RV (reverse primer).

Table S6 | Primers for human genome

Name	Sequence
Satellites II FW	GGAAATGAAAGGGGTCATCATCTAA
Satellites II RV	TTCCGTTTGGTGTTGATACCATTCG
Satellites III FW	CGTTTTACTCCAGTCCACTCCAT
Satellites III RV	AAATGGAATGGAATGGAAGGG
Centromere FW	ATGTTTGCATTCAACTCACAGAG
Centromere RV	CAACACAGTCCAAATATCCAGTTG

*Primers were used for ChIP-qPCR or Top2cc's isolation assays. FW (forward primer), RV (reverse primer).

Table S7 | Primers for qRT-PCR in mouse

Name	Sequence
DNAPK FW	ACAGAAGACAAGTGCAGAAATGGAA
DNAPK RV	CAATGAACTGCCAGCAAGGAAT
SLX4 FW	CCCCACCTGTGGCTCAAATG
SLX4 RV	AGCACGGACTCCAAACCTGTC
Ercc1 FW	CATTTGGATCCCTGGAACAGC
Ercc1 RV	GAGGCACTTTGAGGAAGGGT

*Primers were used for qRT-PCR for quantification of mRNA levels. FW (forward primer), RV (reverse primer).

Uncropped western blot membranes

Sections shown in figures are delimited using dashed rectangles

Shown in Fig. 5d

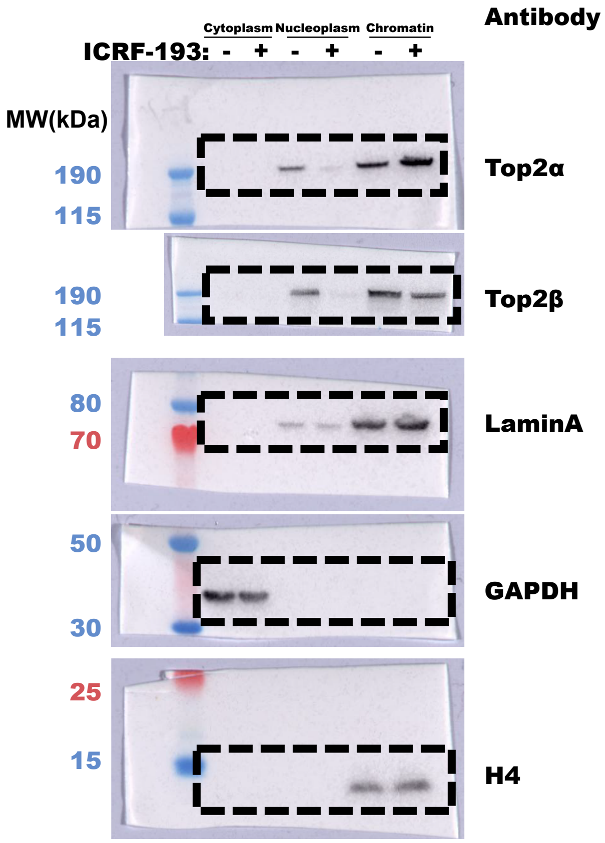

Shown in Fig. 5e

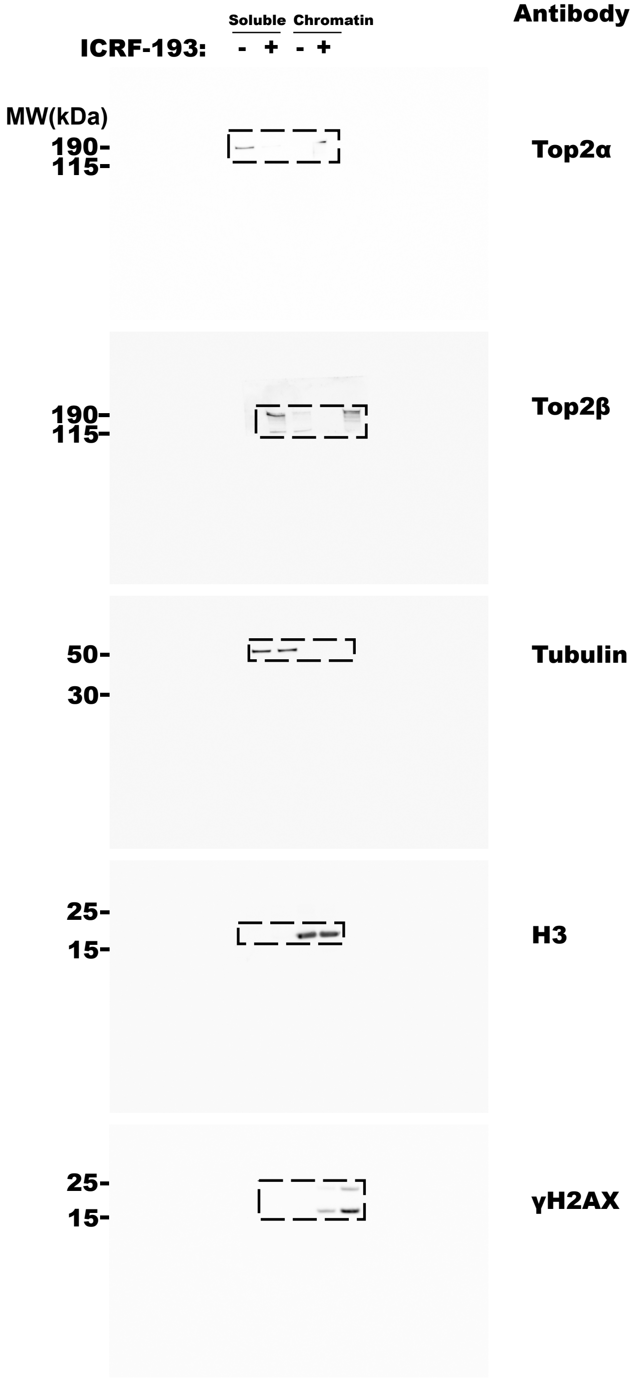

Shown in Fig. 6b

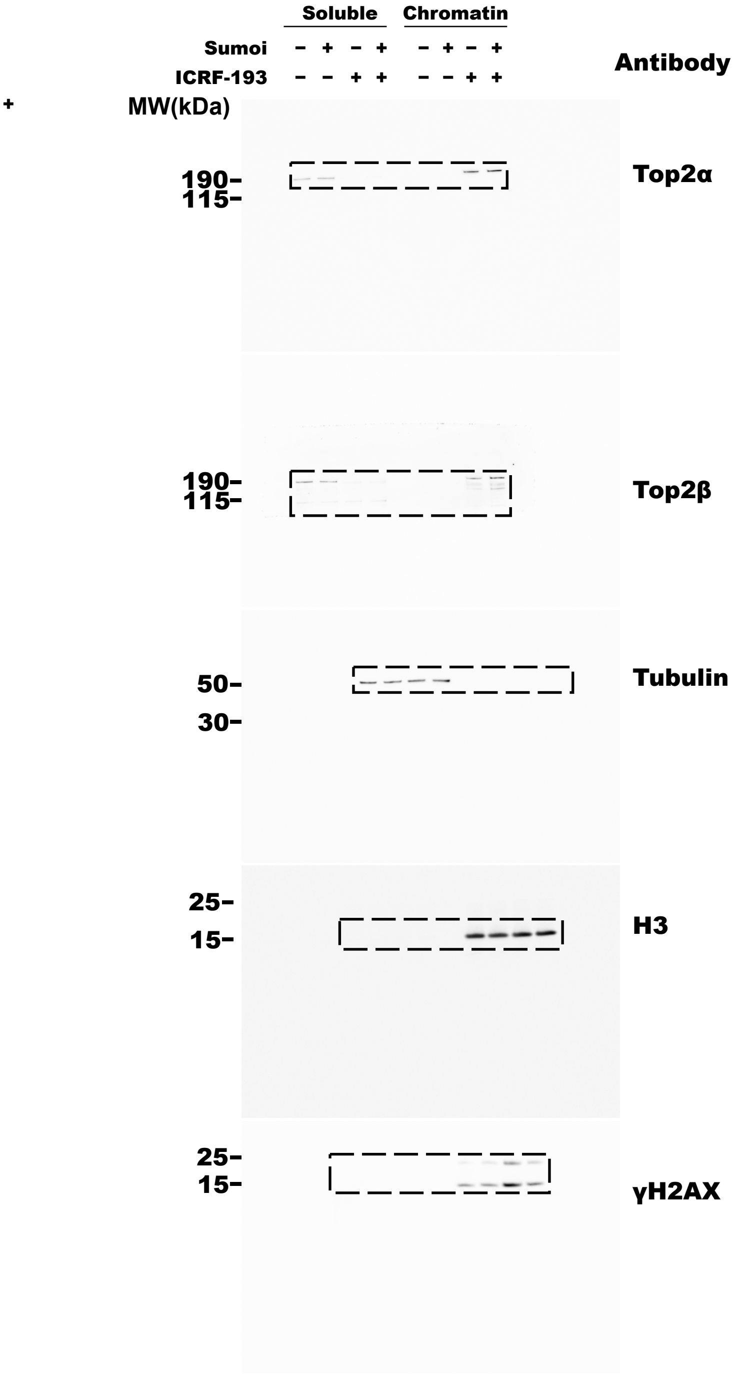

Shown in supplemental Fig. S1a

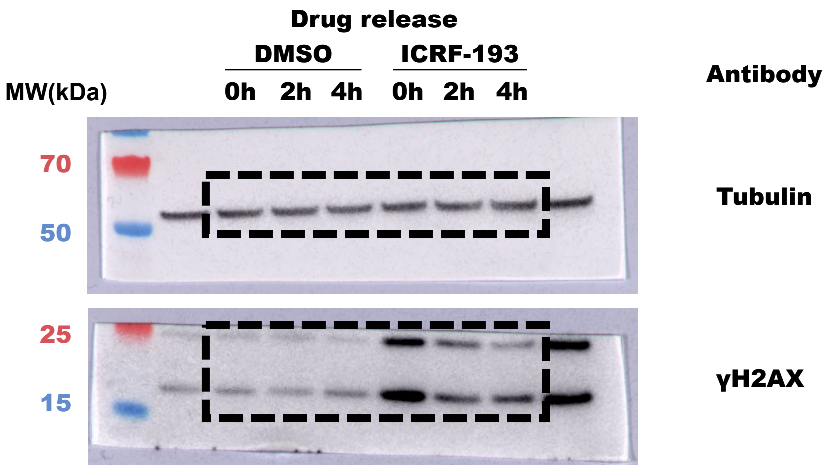

Shown in supplemental Fig. S2k

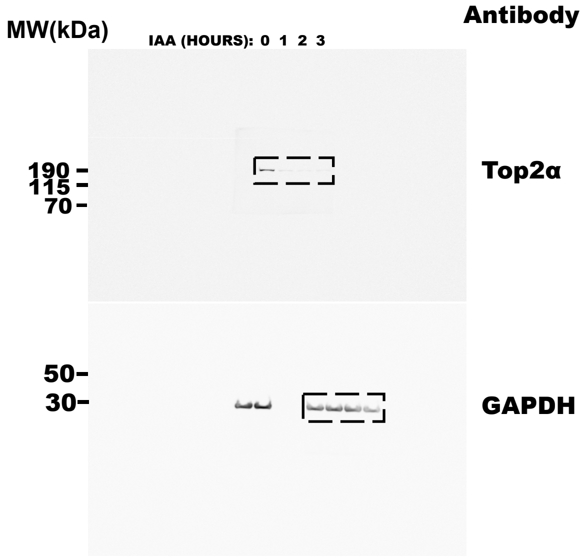

Shown in supplemental Fig. S4a

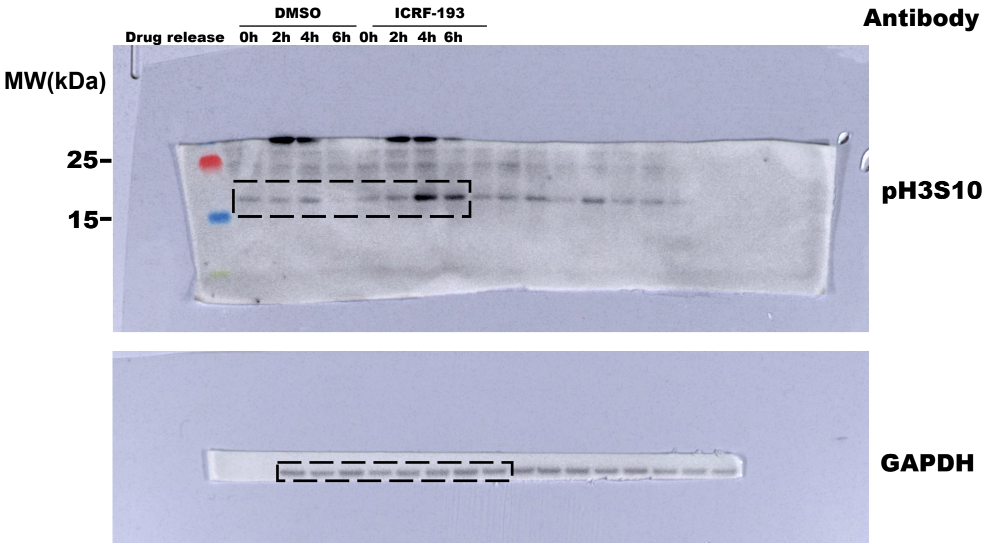

Supplement: Supplementary file 1 — Supplementary Information [file 41467_2024_49816_MOESM1_ESM.pdf]
