# Supplementary material for: Inhibition of topoisomerase 2 catalytic activity impacts the integrity of heterochromatin and repetitive DNA and leads to interlinks between clustered repeats
Source: Nat Commun. 2024 Jul 8;15:5727. doi: 10.1038/s41467-024-49816-7 (PMC11231352; doi:10.1038/s41467-024-49816-7)
Supplement: Supplementary file 3 — Description of Additional Supplementary Files [file 41467_2024_49816_MOESM3_ESM.pdf]

Description of Additional Supplementary Files

File Name: Supplementary Movie 1
Description: Fluorescence recovery after photobleaching (FRAP) performed on untreated (DMSO) NIH3T3 MEF cell over expressing Top2αWt-YFP.

File Name: Supplementary Movie 2
Description: FRAP performed on NIH3T3 MEF cell over expressing Top2αWt-YFP, after 4h treatment with ICRF-193.

File Name: Supplementary Movie 3
Description: FRAP performed on NIH3T3 MEF cell over expressing Top2αWt-YFP and treated with ICRF-193 after pre-treatment with ML-792 sumo inhibitor (Sumoi).
