# Supplementary material for: Inhibition of topoisomerase 2 catalytic activity impacts the integrity of heterochromatin and repetitive DNA and leads to interlinks between clustered repeats
Source: Nat Commun. 2024 Jul 8;15:5727. doi: 10.1038/s41467-024-49816-7 (PMC11231352; doi:10.1038/s41467-024-49816-7)
Supplement: Supplementary file 7 — Reporting Summary [file 41467_2024_49816_MOESM7_ESM.pdf]

nature portfolio

Corresponding author(s): Evi Soutoglou

Last updated by author(s): Apr 30, 2024

Reporting Summary

Nature Portfolio wishes to improve the reproducibility of the work that we publish. This form provides structure for consistency and transparency in reporting. For further information on Nature Portfolio policies, see our Editorial Policies and the Editorial Policy Checklist.

Statistics

For all statistical analyses, confirm that the following items are present in the figure legend, table legend, main text, or Methods section.

n/a	Confirmed	
☐	☒	The exact sample size (*n*) for each experimental group/condition, given as a discrete number and unit of measurement
☐	☒	A statement on whether measurements were taken from distinct samples or whether the same sample was measured repeatedly
☐	☒	The statistical test(s) used AND whether they are one- or two-sided<br>*Only common tests should be described solely by name; describe more complex techniques in the Methods section.*
☒	☐	A description of all covariates tested
☒	☐	A description of any assumptions or corrections, such as tests of normality and adjustment for multiple comparisons
☐	☒	A full description of the statistical parameters including central tendency (e.g. means) or other basic estimates (e.g. regression coefficient) AND variation (e.g. standard deviation) or associated estimates of uncertainty (e.g. confidence intervals)
☐	☒	For null hypothesis testing, the test statistic (e.g. *F*, *t*, *r*) with confidence intervals, effect sizes, degrees of freedom and *P* value noted<br>*Give P values as exact values whenever suitable.*
☒	☐	For Bayesian analysis, information on the choice of priors and Markov chain Monte Carlo settings
☒	☐	For hierarchical and complex designs, identification of the appropriate level for tests and full reporting of outcomes
☒	☐	Estimates of effect sizes (e.g. Cohen's *d*, Pearson's *r*), indicating how they were calculated

Our web collection on statistics for biologists contains articles on many of the points above.

Software and code

Policy information about availability of computer code

Data collection	Zeiss LSM 880<br>Zeiss black software<br>Leica software (LAS X)<br>Confocal laser scanning microscope (TCS SP8; Leica)<br>Olympus ScanR Image Acquisition Software<br>ImageQuant LAS 4000 for western blot<br>AriaMx Real-Time PCR instrument (G8830A) for qPCRs<br>Illumina Hiseq 4000 sequencer for ChIP-seq<br>Comet Assay IV software (Perceptive Instruments, UK)
Data analysis	ImageJ/Fiji software (version 2.1.0/1.53c)<br>Prism (version 8.4.2, GraphPad)<br>Olympus ScanR Image Analysis Software (Version 3.0.1)<br>Spotfire software (version 7.0.1; TIBCO 7.11)<br>Comet Assay IV software (Perceptive Instruments, UK)<br>Agilent AriaMx software (version 1.71)<br>DESEQ2 for statistical analysis<br>Bowtie2, MACS2, HOMER, deepTools

For manuscripts utilizing custom algorithms or software that are central to the research but not yet described in published literature, software must be made available to editors and reviewers. We strongly encourage code deposition in a community repository (e.g. GitHub). See the Nature Portfolio guidelines for submitting code & software for further information.

Data

Policy information about availability of data

All manuscripts must include a data availability statement. This statement should provide the following information, where applicable:

- Accession codes, unique identifiers, or web links for publicly available datasets
- A description of any restrictions on data availability
- For clinical datasets or third party data, please ensure that the statement adheres to our policy

Source data are provided with this paper.

Raw datasets (ChIP-seq) and processed datasets (ChIP-seq) obtained in NIH3T3 MEFs after treatment with ICRF-193, have been deposited in the Gene Expression Omnibus (GEO) under the accession number GSE256090.
(https://www.ncbi.nlm.nih.gov/geo/query/acc.cgi?acc=GSE256090)

Research involving human participants, their data, or biological material

Policy information about studies with human participants or human data. See also policy information about sex, gender (identity/presentation), and sexual orientation and race, ethnicity and racism.

Reporting on sex and gender	N/A
Reporting on race, ethnicity, or other socially relevant groupings	N/A
Population characteristics	N/A
Recruitment	N/A
Ethics oversight	N/A

Note that full information on the approval of the study protocol must also be provided in the manuscript.

Field-specific reporting

Please select the one below that is the best fit for your research. If you are not sure, read the appropriate sections before making your selection.

☒ Life sciences ☐ Behavioural & social sciences ☐ Ecological, evolutionary & environmental sciences

For a reference copy of the document with all sections, see nature.com/documents/nr-reporting-summary-flat.pdf

Life sciences study design

All studies must disclose on these points even when the disclosure is negative.

Sample size	No sample-size calculation was performed. Unless stated otherwise, at least 3 independent biological replicates were performed for the majority of the experiments, which is considered the minimum sample size necessary to draw conclusions based on previous publications. Sample size info is provided in methods-statistics and reproducibility section for each experiment.
Data exclusions	No data were excluded from the analysis
Replication	Biological replicates were performed to validate reproducibility (unless otherwise stated in the figure legend), which was verified by statistical analysis to validate the significance of the results
Randomization	No randomization methods were applicable for the study since cell samples that were compared derived from the same parent cell line, and thus differed only by the treatment or transfection applied.
Blinding	No blinding was performed since the majority of experiments were validated by several different methods and unbiased analysis (i.e. damage induction was validated by confocal microscopy, WB, ChiP-qPCR, ChIP-seq, QIBC etc.); Top2α importance by Confocal microscopy and QIBC; DSB induction by confocal microscopy and Comet assays; Top2α/β localization by confocal microscopy, Top2cc isolation and fractionation assays; Genomic instability by confocal microscopy exoFISH and comet assays; SLX4/Ercc1 importance by siRNAs, Knock-out/rescues, Comet assays etc.

Reporting for specific materials, systems and methods

We require information from authors about some types of materials, experimental systems and methods used in many studies. Here, indicate whether each material, system or method listed is relevant to your study. If you are not sure if a list item applies to your research, read the appropriate section before selecting a response.

Materials & experimental systems

n/a	Involved in the study
☐	☒ Antibodies
☐	☒ Eukaryotic cell lines
☒	☐ Palaeontology and archaeology
☒	☐ Animals and other organisms
☒	☐ Clinical data
☒	☐ Dual use research of concern
☒	☐ Plants

Methods

n/a	Involved in the study
☐	☒ ChIP-seq
☒	☐ Flow cytometry
☒	☐ MRI-based neuroimaging

Antibodies

Antibodies used

γH2AX - Mouse Abcam/ab22551 IF & WB (1:1000)
γH2AX - Rabbit Abcam/ab2893 IF (1:1000) & ChIP (5μg/IP)
pH3S10 - Rabbit Merck/06-570 IF & WB (1:1000)
Top2α – Mouse Santacruz/sc-365916 (1:200)
Top2β - Rabbit ThermoFisher/ PA5-54984 (1:250)
53BP1 - Rabbit Novus Biologicals/100-304
Brca1 - Mouse Gift from Dr. Andre Nussenzweig
RPA32 - Mouse Novus Biologicals/600-565
Rad51 - Rabbit Calbiochem/ PC130
Ercc1 – Mouse Santacruz/sc-17809 (1:150)
HP1α - Mouse Euromedex/ 2HP1H5
H3K9me3 - Rabbit Abcam/ ab8898
Lamin A – Rabbit Abcam/ab26300
GAPDH – Mouse Millipore/MAB374
Tubulin – Mouse Merck/T6074
H3 - Rabbit Abcam/ab1791
H4 Abcam/ab7311
GFP - Mouse Santacruz/sc-9996

Validation

γ-H2AX, 53BP1, RAD51, RPA32, α-Tubulin, HP1α, H3K9me3 and phH3S10 antibodies have been previously validated in our manuscript Tsouroula, K. et al. Temporal and Spatial Uncoupling of DNA Double Strand Break Repair Pathways within Mammalian Heterochromatin. Mol. Cell 63, 293–305 (2016). Brca1 antibody (Gift from Dr. Andre Nussenzweig) has been previously validated in our manuscript Mitrentsi, I. et al. Heterochromatic repeat clustering imposes a physical barrier on homologous recombination to prevent chromosomal translocations. Mol Cell 82, 2132-2147 e2136, doi:10.1016/j.molcel.2022.03.033 (2022).

Lamin A antibody (Abcam, ab26300) was validated by the manufacturer using mouse and human cells by WB. We validated Ercc1 antibody (Santacruz/sc-17809) by measuring the number of Ercc1 foci after siErcc1 in figure S7c. GAPDH (Millipore/MAB374) antibody was validated by the manufacturer using human (Hela) and mouse (NIH3T3) cells by IF and WB. H3 (Abcam/ab1791) was validated by the manufacturer using human (Hela) and mouse (NIH3T3) cells by IF and WB. H4 (Abcam/ab7311) and GFP (Santacruz/sc-9996) were validated by the manufacturer. Top2α (Santacruz/sc-365916) and Top2β (ThermoFisher/ PA5-54984) antibodies were validated in our study with immunofluorescence experiments after siRNA transfections.

Eukaryotic cell lines

Policy information about cell lines and Sex and Gender in Research

Cell line source(s)

NIH3T3 (ATCC), U2OS (ATCC), RPE1 (ATCC), Ku80 knock-out MEFs (Gift from Dr. Andre Nussenzweig), HCT116 cells expressing OsTIR1 and Top2α-mAID (Gift from Dr. Christian Thomas Friberg Nielsen), SLX4 WT and knock-out MEFs (Gift from Dr. John Rouse), Top2βKO MEFs (GDSC cell bank), RPE1 Top2βKO cells (From Will Gittens/Matt Neale)

Authentication

All cell lines from ATCC were authenticated by the vendor using short tandem repeat (STR) profiling. For the HCT116 cells expressing OsTIR1 and Top2α-mAID (Gift from Dr. Christian Thomas Friberg Nielsen), we performed WB to validate the downregulation of Top2α after 2-3h treatment with IAA. For the SLX4 WT and knock-out MEFs (Gift from Dr. John Rouse) we did not authenticate them ourselves in the lab.

Mycoplasma contamination

All cell lines were tested and were mycoplasma free.

Commonly misidentified lines
(See ICLAC register)

No commonly misidentified cell lines were used for this study

Plants

Seed stocks: N/A

Novel plant genotypes: N/A

Authentication: N/A

ChIP-seq

Data deposition

☒ Confirm that both raw and final processed data have been deposited in a public database such as GEO.

☒ Confirm that you have deposited or provided access to graph files (e.g. BED files) for the called peaks.

Data access links
May remain private before publication.

https://www.ncbi.nlm.nih.gov/geo/query/acc.cgi?acc=GSE256090

Files in database submission

GSM8085504 Input DMSO rep1
GSM8085505 Input ICRF 0h rls rep1
GSM8085506 Input ICRF 3h rls rep1
GSM8085507 γH2AX ChIP-Seq DMSO rep1
GSM8085508 γH2AX ChIP-Seq ICRF 0h rls rep1
GSM8085509 γH2AX ChIP-Seq ICRF 3h rls rep1
GSM8085510 Input DMSO rep2
GSM8085511 Input ICRF 0h rls rep2
GSM8085512 Input ICRF 3h rls rep2
GSM8085513 γH2AX ChIP-Seq DMSO rep2
GSM8085514 γH2AX ChIP-Seq ICRF 0h rls rep2
GSM8085515 γH2AX ChIP-Seq ICRF 3h rls rep2

Genome browser session
(e.g. UCSC)

no longer applicable

Methodology

Replicates: 2 biological replicates were performed, both consistent with the results

Sequencing depth:
Total number of reads: 39-70 million
Paired-end 100 bases
Multiple mapped reads were taken into consideration to analyze the effects on heterochromatin

Antibodies: γH2AX - Rabbit, Abcam: ab2893, Concentration: 5µg/IP

Peak calling parameters: Reads were mapped to the mm10 mouse genome assembly using Bowtie262 with the parameters: -q -N 1 -X 1000. Since we were interested in repetitive sequences, multiple mapped reads were taken into consideration for the analysis. The peak calling was performed by MACS2 analysis and peak annotation by HOMER, using the default settings. Bigwig files were generated using bamCoverage from deepTools software63 with RPKM normalization.

Data quality: *Describe the methods used to ensure data quality in full detail, including how many peaks are at FDR 5% and above 5-fold enrichment.*

Software: annotatePeaks.pl from Homer software
